# Salt tolerance in wheat is associated with the maintenance of shoot biomass, stomatal conductance, and sucrose in the phloem

**DOI:** 10.1002/pei3.70008

**Published:** 2024-09-11

**Authors:** Van Lam Nguyen, James Stangoulis

**Affiliations:** ^1^ College of Science and Engineering, Flinders University Bedford Park South Australia Australia

**Keywords:** phloem sucrose, photosynthesis, salinity tolerance, shoot traits, wheat

## Abstract

Wheat (*Triticum aestivum* L.) is a mega‐staple for millions of the world's populations and its yield potential is impacted by soil salinization. This study investigated genotypic variation in salt tolerance among six wheat genotypes, Gladius, Drysdale, GD0014, GD0120, GD0180, and GD0185. The study also characterized shoot traits, photosynthetic traits, leaf Na and K concentrations, and phloem sucrose. The plants were grown under controlled growth room conditions at 0 mM NaCl (Control) and 100 mM NaCl. The results showed that the salt tolerance index (STI_SFW_, SFW: shoot fresh weight) varied from 0.52 for GD0120 to 0.69 for GD0180. Based on the STI_SFW_, salt tolerance for the wheat genotypes was in the order, GD0180 > Gladius > GD0185 > Drysdale > GD0014 > GD0120. Projected shoot area (PSA) at all growth stages, 14, 20, 27, 34, and 40 DAS were strongly correlated with SFW at 45 DAS. Salt treatment significantly increased phloem sucrose level in the salt intolerant, Drysdale, while having no effect on this parameter in Gladius. Gladius showed greater maintenance of stomatal conductance than Drysdale. The relative ratio of K/Na between treatment and control was strongly correlated with the relative ratio of SFW (*r* = .85). The correlation between PSA at 14 DAS and SFW at 45 DAS and the correlation between the relative ratio of K/Na between treatment and control with STI_SFW_ identify these parameters to be potential traits for screening salt tolerance in wheat. Higher salt tolerance in Gladius would be associated with higher maintenance of stomatal conductance and enhanced phloem sucrose transport.

## 1 INTRODUCTION

Wheat (*Triticum aestivum* L.) is a staple food to many of the world's population and is the second most important crop worldwide, accounting for nearly 20% of staple food produced (Shiferaw et al., 2013). Not only being a major source of starch and energy, wheat also contributes to adequate protein, vitamin, and dietary fiber consumption (Shewry & Hey, 2015). Although the worldwide cropping area has not increased for wheat, the yield has significantly increased from 2.5 mt/ha during 1992–1994 to 3.5 mt/ha during 2016–2021 and resulting in an increase of around 36% in production (Erenstein et al., 2022). Considering a growing worldwide population and arable land affected by climate change, wheat production will need to increase. Unfortunately, abiotic stress will continue to negatively impact on yield gains, with salt stress a major concern with 20% of the arable land in the world affected by salinity (Li et al., 2022). Therefore, the investigation of salt tolerance mechanisms in wheat as well as the development of a rapid method to screen salt‐tolerant wheat is important to increase wheat yield.

Salinity tolerance is centered around three main mechanisms; osmotic tolerance, ion exclusion, and tissue tolerance (Roy et al., 2014), and plants respond to salinity in two distinct phases, the osmotic phase and an ionic phase (Soni et al., 2021). In the osmotic phase, plants use osmotic tolerance to mitigate the stress, and when Na and Cl enter the plant tissues, the plants can use ion exclusion and tissue tolerance to reduce the effect of these ions (Munns & Tester, 2008). Plants adapt to osmotic stress by enhanced accumulation of proline, glycine, and sugar metabolites (Darko et al., 2019) and a decreased stomatal aperture (Tavakol et al., 2021), leading to a reduction in stomatal conductance and transpiration rate (Elhakem, 2020; Zahra et al., 2022). Higher salt concentrations entering the plant can be excluded from leaves (James et al., 2023) or are sequestrated into vacuoles to reduce Na^+^ in the cytosol and thereby also inhibiting K^+^ loss, thereby assisting in maintaining cytosolic K^+^ homeostasis (Tang et al., 2020). Alternation of phloem metabolites such as sugars can be an approach to adapt to salt stress (Ma et al., 2020). For example, salinity induces the accumulation of carbohydrates and sugar‐regulated starch biosynthetic genes (Kumar et al., 2018; Yin et al., 2009).

Ernst Munch hypothesized that the transport of sugars and other molecules through the phloem from source tissues such as leaves to sink tissues such as roots and fruits, is based on pressure differences (Knoblauch et al., 2016). The molecules loading into phloem sieve elements in the source tissues make the fluid inside the phloem more concentrated so that the water from the xylem vessels is drawn into the phloem. This creates pressure and pushes the solution along the phloem vessel towards the sink tissues. In the sink tissues, active unloading of the sugars results in a reduction in the phloem sugar levels and a reduction in pressure. Salinity is known to reduce sugar synthesis and water uptake in plants (Hannachi et al., 2022; Lu & Fricke, 2023). Therefore, salinity lowers the xylem–phloem transfer of water in the source tissue (Chattha et al., 2024), and thereby sugar transport in the phloem. It is hypothesized that the salt‐sensitive wheat has a reduced capacity in acquiring water from the xylem, which results in an increased accumulation of sucrose in the phloem.

Screening wheat genotypes for salt tolerance is challenging due to the lack of effective methods for evaluation (Quan et al., 2021). Furthermore, mechanisms of salt tolerance vary with the stage of plant development and this makes selection more complex (Hussain et al., 2021). Thus, identification of what parameters are suitable for screening and understanding the mechanism of salt tolerance is vital. A single parameter (i.e., yield or height) can be used to evaluate salt tolerance in wheat, but a set of attributes such as morphological, physiological, and biochemical factors are often considered (Irshad et al., 2022; Mansour et al., 2020). Regarding morphological traits, the ratio of yield between the salt treatment and the control is a common parameter for salt tolerance screening (Quan et al., 2021; Tao et al., 2021). However, this method can only be used after plants are harvested, and a more rapid screening technique is needed. Imaging techniques can be used to eliminate this drawback, by analyzing changes in the reflectance spectrum of plants after treatment (Moghimi et al., 2018). Imaging techniques have also been used to measure projected shoot area (Armoniené et al., 2018; Shamaya, 2014); however, how this value varies at different growth stages when screening salt tolerance in wheat is still limited. Variations in photosynthesis and osmotic adjustment compounds have been used for screening salt‐tolerant plants (Hussain et al., 2021), but analysis can often be costly and time consuming.

The response of wheat to salinity varies at different growth stages (Armoniené et al., 2018) and therefore, this study aimed to investigate how shoot parameters measured by imaging analysis (i.e., projected shoot area, convex hull area) of six selected wheat genotypes respond to salt treatment during their growth stage. The study also examined if shoot parameters at early stages can discriminate between wheat genotypes, challenged with salt toxicity. Physiological mechanisms of salt tolerance were also investigated through the measurement of phloem sucrose level, gas exchange, chlorophyll fluorescence (OJIP), leaf water content and leaf Na^+^, K^+^, and other mineral concentrations.

## 2 MATERIALS AND METHODS

### 2.1 Plant growth

Six wheat (*Triticum aestivum* L.) genotypes including varieties Gladius and Drysdale, and four recombinant inbred lines, GD014, GD120, GD180, and GD185 derived from a Gladius × Drysdale cross, in which Gladius was shown to have greater salt tolerance index than Drysdale (Shamaya, 2014). Prior to planting, the seed was washed in high‐purity water (>18.2 MΩ cm^−1^ resistivity) and pre‐germinated for 3 days in petridishes lined with wet filter paper. Seedlings were planted in Scotts Osmocote Premium Plus Superior Potting Mix® (a commercial product enriched with fertilizers, https://www.bunnings.com.au/scotts‐osmocote‐50l‐premium‐plus‐superior‐potting‐mix_p2962101). Plants were grown in square‐shaped 18.0 cm high × 8.5 cm top wide × 6.5 cm bottom wide pots filled with 0.4 kg of the potting mix. One seedling was planted into each pot with four biological replicates and grown into a Conviron CMP6050 growth room. Growth room conditions were 13/11 h light/dark at 20/10°C with the light intensity of 500 μmol m^−2^ s^−1^ photosynthetic photon flux density at the leaf surface. Plants were watered three times a week to 25% of soil weight.

Salt treatments commenced after 2 weeks of growth and at the rate of 100 mM. The treatment was implemented by watering the salt solution and the control was watered with RO water, three times every week up to 25% of the soil weight. The plants were watered from the surface of the soil and the amount of water was added based on the water loss using a balance.

### 2.2 Gas exchange and chlorophyll fluorescence measurement

Gas exchange was measured using a portable photosynthesis system (LI‐6800, LI‐COR, United States). Gas exchange was measured at 35 and 42 DAS and the measurements were taken from 10:00 to 14:30 h (the daylight hours were from 6 am to 7 pm). The measurements were made at the middle of the youngest fully expanded leaves. The light source was the Multiphase Flash™ Fluorometer (6800‐01A, LI‐COR, United States) and light intensity was set at 700 μmol m^−2^ s^−1^ (90% red and 10% blue) with an aperture of 2 cm^2^. All photosynthetic measurements were taken at a constant airflow rate of 500 μmol s^−1^. The CO_2_ concentration supplied was 400 μmol mol^−1^ and the temperature (Tleaf) was 22°C. The chamber humidity was controlled by setting the leaf vapor pressure deficit (VDP_leaf_) at 1. The measured leaf area was adjusted by the method developed by Savvides and Fotopoulos (2018).

Chlorophyll fluorescence was measured by using a hand‐held meter (FluorPen FP 100, Photon System Instruments, Drásov, Czech Republic). Two methods were used to measure chlorophyll fluorescence, OJIP at 41 DAS and NPQ (NPQ1) at 44 DAS. The measurements were taken on dark‐adapted leaves. F_pulse, f_pulse, and A_pulse were set at 50%, 30%, and 10%, respectively. Leaves were dark adapted using aluminum foil for 1 h before measurement. Measurements were taken in the middle of the youngest fully expanded leaf in a dark room.

### 2.3 Greenness measurement

The measurements for Normalized Difference Vegetation Index (NDVI) were measured on the middle of the youngest fully expanded leaf using a hand‐held meter (PlantPen NDVI 300, Photon System Instruments, Drásov, Czech Republic). The measurement was taken at a growth stage of 36 DAS.

### 2.4 Leaf length, leaf with, shoot projected area, convex hull area, shoot mass, and leaf water content

Leaf length and leaf width were measured on the youngest fully expanded leaf using a ruler. The width was measured in the middle of the leaf. Leaf water content was measured based on the water loss by drying the samples in an oven at 85°C for 3 days.

Shoot projected area (pixels of shoot images converted into cm^2^), convex hull area (the smallest convex set that encloses the plant), shoot width, and shoot height were measured using Plantcv at 14, 20, 27, 34, and 40 DAS (Gehan et al., 2017). Plant images were taken using an iPhone 6 installed on a tripod (Vivitar 7‐in‐1 Tripod) (Figure S1). At 14 DAS, the plant images were taken side view for one side and at the rest of the growth stages, the image of plants was captured for two side views and top view. The images included round markers (1.2 cm) for conversion from pixels to cm after analysis. Shoot parameters were analyzed using Plantcv in JupyterLab of Anaconda 3 (version 3.8.5) using codes developed by Gehan et al. (2017) with adjustment (Figure S2). The side view parameters were calculated as the mean of two sides.

Plants were harvested at 45 DAS and before harvest, two of the youngest fully expanded leaves were collected. Leaves were put in 50‐mL tubes and plunged into liquid nitrogen before being stored at −80°C. At harvest, shoots were detached just above soil level. The shoot fresh weight was measured and then dried in an oven at 85°C for 3 days. The shoot mass was measured using a Mettler Toledo balance.

The salt tolerance index (STI) was evaluated based on the shoot fresh mass and projected shoot area (PSA) and calculated as below:

$${\mathrm{STI}}_{\mathrm{PSA}-\mathrm{SV}}=\frac{\mathrm{PSA}-\text{side view of salt treated plants}}{\mathrm{PSA}-\text{side view of control plants}}$$

$${\mathrm{STI}}_{\mathrm{PSA}-\mathrm{TV}}=\frac{\mathrm{PSA}-\mathrm{top}\;\text{view of salt treated plants}}{\mathrm{PSA}-\mathrm{top}\;\text{view of control plants}}$$

$${\mathrm{STI}}_{\mathrm{SFW}}=\frac{\text{Shoot fresh weight of salt treated plants}}{\text{Shoot fresh weight of control plants}}$$

### 2.5 Mineral analysis

The minerals of the youngest fully expanded leaves collected at 45 DAS were analyzed at a commercial analytical facility, APAL. Leaves were dried in an oven at 85°C for 2 days and then milled using a Mixer mill (Retsch MM400, Germany) in 2‐mL Eppendorf tubes with a stainless‐steel ball for 90 s. The fine leaf power was then digested by microwave in nitric acid/hydrogen peroxide and analyzed by ICP‐MS at APAL (Eurofins APAL Pty Ltd, South Australia, Australia). The mineral concentration was represented as g/kg or mg/kg of dried weight.

### 2.6 Phloem extraction and sucrose analysis

Seeds of two wheat genotypes, Drysdale and Gladius, were germinated on tissue paper soaked with MilliQ water in petri‐dishes for 4 days before transplanting. Petri dishes were flooded with MilliQ water prior to transplant to allow the release of roots from the tissue without damage. Seedlings were propagated into 70 mm diameter pots filled with an average 215 g of Debco™ Green Wizard potting mix. Pots were placed into a Conviron CMP6050 growth room with conditions set to a 13 h light, 11 hr dark cycle with temperatures at 20°C/10°C, respectively. A minimum of 400 μmol m^−2^ s^−1^ light at the leaf surface was generated by a fluorescent and candescent globe a combination.

Plants were well watered two to three times with MilliQ water a week depending on the requirements of plants. The salt treatment was commenced at 21 days after sowing (DAS) by watering with 100 mM NaCl solution and the control was watered with MilliQ water. The water regime was two to three times with MilliQ water a week depending on the requirements of plants.

The phloem extract was collected using aphid a stylectomy method (Palmer et al., 2013; Palmer & Stangoulis, 2018). An anholocyclic population of *Rhopalosiphum padi* (Oatmeal Aphid) was maintained on cereal plants within enclosures at the Flinders University campus. Apterous specimens taken from this population were secured by caging a minimum of 18 h prior to stylectomy. For initial samples, cages were attached to the plant stem. Placement of aphids to the mid peduncle. Plants were watered with 100 mL MilliQ and 100 mM NaCl solution for the control and treatment, respectively, immediately prior to caging to increase hydrostatic pressure in the phloem.

Samples were collected via the air collection method, using borosilicate glass micro‐capillaries (30–0017, Harvard Apparatus) backfilled with water‐saturated paraffin oil (Downing, 1978). Sample volumes were calculated based on four‐time sequence images taken 2 s apart, with 15‐min intervals between images captured. Flow rates were calculated based on the difference in droplet diameter in each sequence. The collected volume was calculated based on the method developed by Palmer et al. (2013).

The initial sequence was taken immediately after a flowing stylet had been cut and the final sequence was taken immediately prior to termination of the collection PE capture. Where PE flow rates allowed the sample time was 45 min. If this timeframe was not achieved, then the actual collection was recorded and calculations adjusted to reflect the length of sample capture.

Once collected, samples were stored in a 250 μL vial insert (5181‐1270, Agilent) housed within a 2 mL short thread vial (THC11 09500, Thermo Scientific). Each vial had 10 μL of MilliQ water placed into the insert. PE was released into MQ water which was used to rinse the capillary three times prior to storage. All samples were stored at −80°C until preparation for analysis was done. The samples were then freeze‐dried and were reconstituted using 50 μL of internal standarsd solution containing 100 μM Rhamnose.

The samples were diluted 10 times prior to being analyzed using high‐performance anion exchange chromatography coupled to pulsed amperometric detection (HPAEC‐PAD) (Dionex ICS3000, Thermo Fisher Scientific) described by Nguyen et al. (2011). The injection volume was 10 μL and sucrose concentration was calculated based on sucrose standards of 10, 25, 50, 100, 250, and 500 μM.

### 2.7 Statistical analysis

R (version 4.3.0) was used to perform statistical analysis. Two‐way ANOVA analysis was used to analyze the effect of genotype (G) and salt treatment (T) on fresh shoot weight, leaf water content, OJIP parameters, leaf Na, K, other minerals concentration, K/Na concentration, and sucrose level in phloem. Simple effect and interaction analysis were performed to compare differences between genotypes at each P supply for these traits using the Phia package developed by De Rosario Martinez (2015) with Bonferroni correction. Main effect and simple effect contrasts were analyzed using the Tukey's HSD test (*p* < .05) for parameters with the equality of variance and the Games Howell test (*p* < .05) for parameters without the equality of variance (r‐project, n.d.). The effect of genotype, P supply, and growth stage on shoot parameters, leaf length, leaf width, and gas exchange traits were analyzed using three‐way ANOVAs. Simple interactions and simple effects were performed to analyze the interactions and effects for two variables at a specific level of the third variable (Kirk, 2014; UCLA, n.d.). Simple effects were also performed to analyze the effects for one variable at a specific level of the other two variables. The error term for simple interaction and simple effect analysis was corrected with the error term of the entire data. The *F* statistic for a significant effect was corrected using two methods, the family error rate and Dun's method (Kirk, 2014). The correlation matrix analyses between shoot traits, between gas exchange, shoot weight and water content, and between leaf Na, K and K/Na, and fresh shoot weight were performed using the Hmisc package and Pearson's method.

## 3 RESULTS

### 3.1 Salt treatment and genotype effect on shoot parameters

**FIGURE 1:**
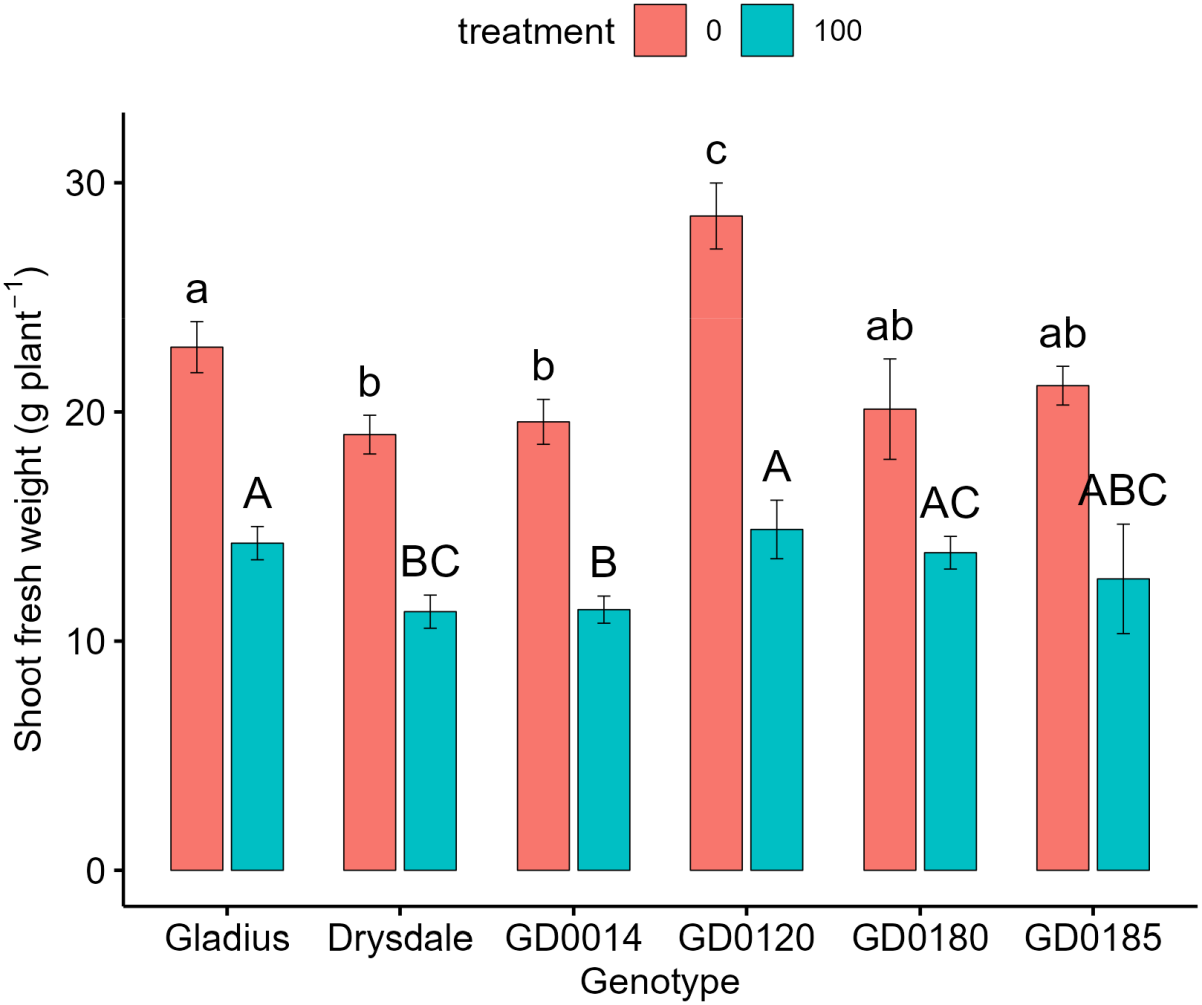
Effect of salt treatment on shoot fresh weight of six wheat genotypes at 45 days after sowing (DAS). Data represent the mean and standard deviation of four replicates. Treatment: 0 mM and 100 mM NaCl. Different letters showed significant differences among genotypes at each salt treatment.

As shown in Figure 1 and Table S1, genotype and salt treatment had significant effects (*p* < .001) on fresh shoot weight. A significant interaction (*p* < .001) between genotype (G) and treatment (T) was observed for shoot fresh weight (Figure 4; Table S1). When treatments were presented as graphs, the control, and shoot fresh weight were higher than under treatment in all genotypes (Figure S7). Therefore, this was an ordinal interaction and the main effect contrast analysis showed the salt treatment significantly (*p* < .001) reduced shoot fresh weight by 40.1% compared to the control (21.99 vs. 13.11 g plant^−1^).

When genotypes were shown as graphs (Figure S7), the interaction between genotype and treatment was disordinal since there was a cross‐over between lines. Thus, the simple effect analysis of genotype at each salt treatment was conducted. Fresh shoot weight significantly varied between the wheat genotypes at each salt treatment (Table S4a,b). In the control, the shoot fresh weight of GD0120 was significantly higher than that of all the other genotypes (Figure 4). Under this condition, fresh shoot weight in GD0120 was 50% greater than in the lowest genotype, Drysdale (19.01 ± 0.85 g plant^−1^). Gladius also had significantly higher fresh shoot weight than Drysdale and GD0014 under control conditions where the fresh shoot weight of Gladius was 20% higher than that in Drysdale (Table S1).

Under salt treatment, GD0120 (14.87 ± 1.28 g plant^−1^) showed significantly higher fresh shoot weight compared to Drysdale (11.28 ± 0.72 g plant^−1^) and GD0014 (11.37 ± 0.59 g plant^−1^) (Figure 1). Drysdale had the lowest fresh shoot weight under this condition, and it was 24% and 20% lower than in GD0120 and Gladius, respectively (Figure 1).

STI_SFW_ varied widely from 0.52 for GD0120 to 0.69 for GD0180. Gladius had a medium ratio of 0.63 (Table 1). This indicates that GD0120 was more salt sensitive, while GD0180 was more salt tolerant. Gladius appears to be moderately tolerant.

**TABLE 1:** The ratio of fresh shoot weight between salt treatment (100 mM) and without treatment of six wheat genotypes at 45 days after sowing (DAS).

Genotype	Ratio
Gladius	0.63
Drysdale	0.59
GD0014	0.58
GD0120	0.52
GD0180	0.69
GD0185	0.60

**FIGURE 2:**
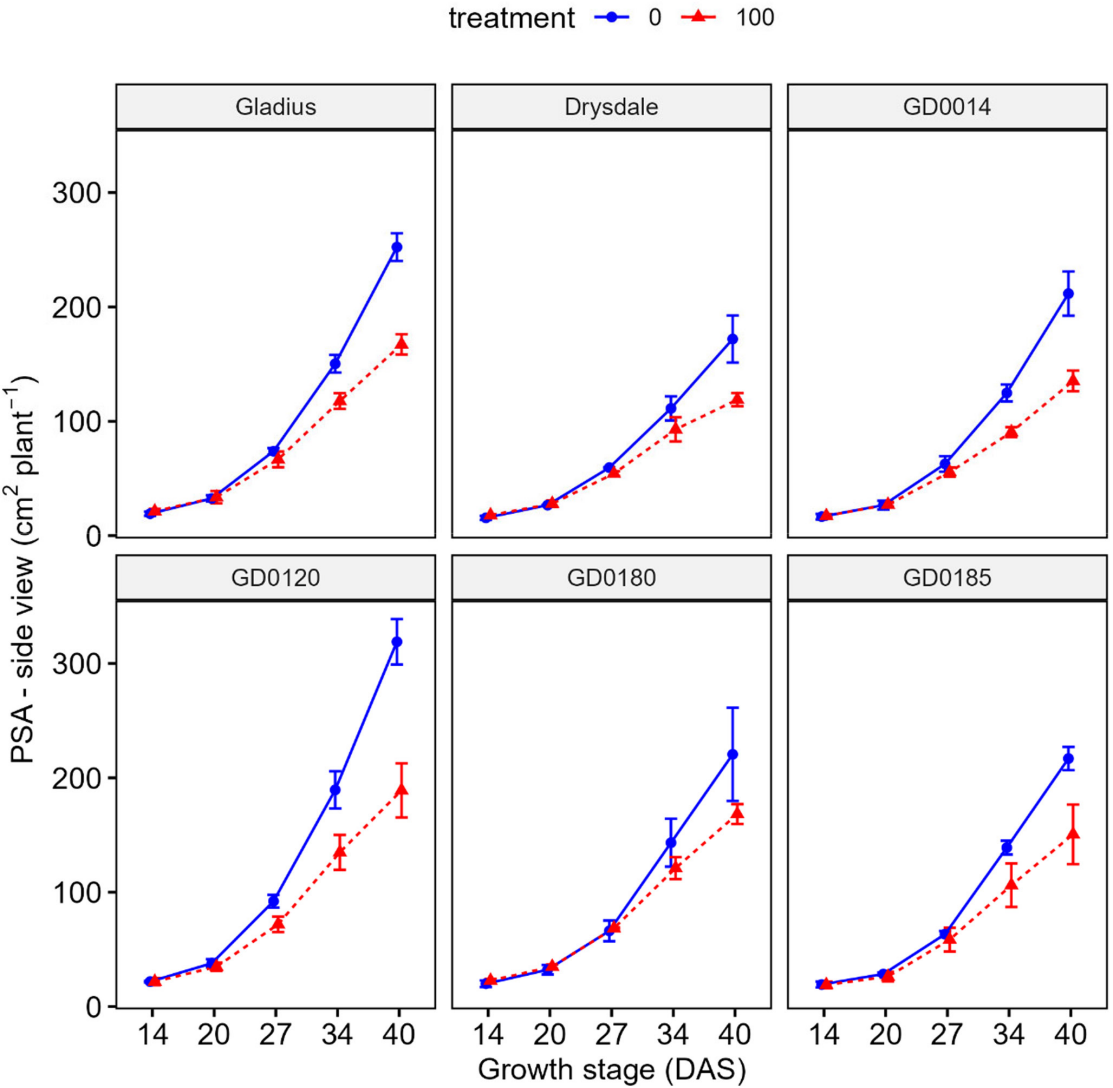
Effect of salt treatment on projected shoot area (PSA)—side view of six wheat genotypes at different growth stages. Data represent the mean and standard deviation of four replicates. Treatment: 0 mM and 100 mM NaCl. DAS, days after sowing.

**FIGURE 3:**
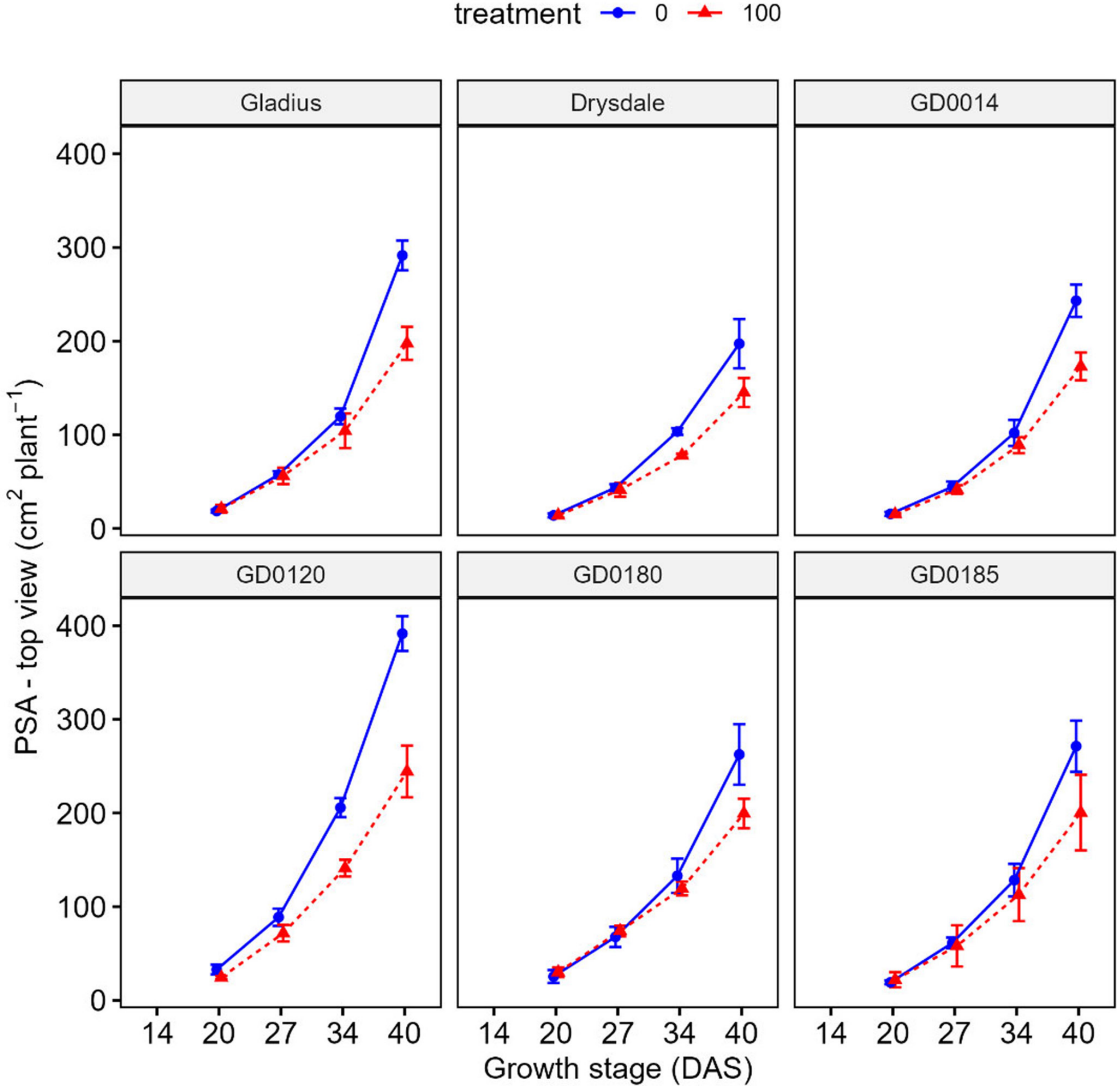
Effect of salt treatment on projected shoot area (PSA)—top view of six wheat genotypes at different growth stages. Data represent the mean and standard deviation of four replicates. Treatment: 0 and 100 mM NaCl. DAS, days after sowing.

**FIGURE 4:**
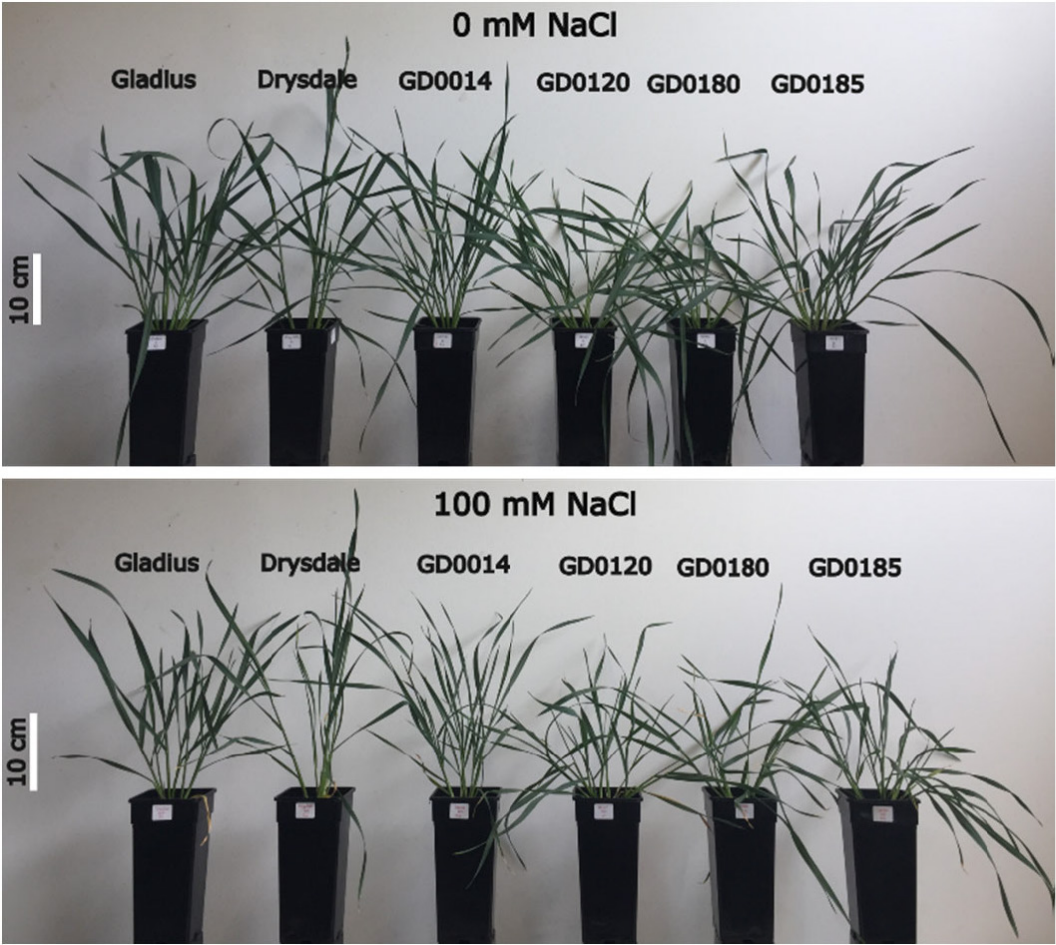
Effect of salt treatment on six wheat genotypes at 40 days after sowing (DAS).

After 20 days of salt treatment (34 DAS), there was a significant (*p* < .05) effect on the projected shoot area (PSA) (Figures 2 and 3; Figures S8 and S9; Table S3). The effect of salt treatment can also be seen in the visualized plant images at 40 DAS (Figure 4). The effect was greater when the time of treatment was longer. For example, STI_PSA‐SV_ reduced gradually from 14 DAS (1.06) to 27 DAS (0.89) then significantly decreased to 0.66 at 40 DAS (Table S4). STI_PSA‐TV_ has a similar trend.

When using the projected shoot area to evaluate salt tolerance, salt tolerance varied among the wheat genotypes. GD0120 relatively showed response earlier than other genotypes. As in Table S2, at 27 DAS STI_PSA‐SV_ and STI_PSA‐TV_ were the lowest, 0.78 and 0.81, while these values of Gladius were 0.90 and 0.98 (Table S4).

Salt treatment showed an effect on convex hull area (CHA); however, the degree in which it was affected, varied between the genotypes (Figures S3, S4, S10, S11; Table S3). Simple contrast analysis between treatments showed that salt treatment had no significant effect on CHA – side view of Drysdale, GD0180 and GD0185 at all growth stages, while a significant effect of salt treatment occurred on GD0120 at 34 and 40 DAS, on Gladius and GD0014 at 40 DAS. Salt treatment had no significant effect on CHA‐top view of GD0014, GD0180, and GD0185 at all growth stages, while the treatment showed a significant effect on this parameter of Gladius, Drysdale, and GD120 at 40 DAS.

### 3.2 Salt treatment effects on leaf length, leaf width, leaf water content, and leaf greenness

Salt treatment significantly reduced leaf length at 34 and 40 DAS in Gladius and Drysdale, and at 40 DAS in GD0014, GD0120, and GD0180 (Figure S5). In contrast, salt treatment had no significant effect on leaf length of GD0185 at all growth stages (Figure S5). GD0180 showed a longer leaf than other genotypes (Figure S12). Regarding leaf width, the salt treatment decreased the leaf width of GD0120 at three growth stages, 27, 34, and 40 DAS (Figure S6). GD0014 and GD0185 had small leaf width (Figure S13).

Both genotype and salt treatment had a significant effect on leaf water content (*p* < .01). No significant G × T interaction for this parameter was observed (Table S1). The main effect contrast analysis showed that salt treatment significantly reduced leaf water content to 80.07 ± 1.53 (%) when compared to the control (82.85% ± 1.96%). Drysdale had the lowest leaf water content (79.17% ± 2.54%) and it was significantly lower than in other genotypes, except for GD0120 (Table S1).

Salt treatment did not affect greenness, while genotype had a significant (*p* < .01) effect on this parameter. GD0014 and GD0185 showed the lowest greenness, 0.76 ± 0.01 and 0.76 ± 0.02, respectively, while Drysdale had the highest greenness, 0.79 ± 0.01 (Table S1).

### 3.3 Correlation between shoot parameters, leaf length, leaf width, leaf water content, and greenness

**FIGURE 5:**
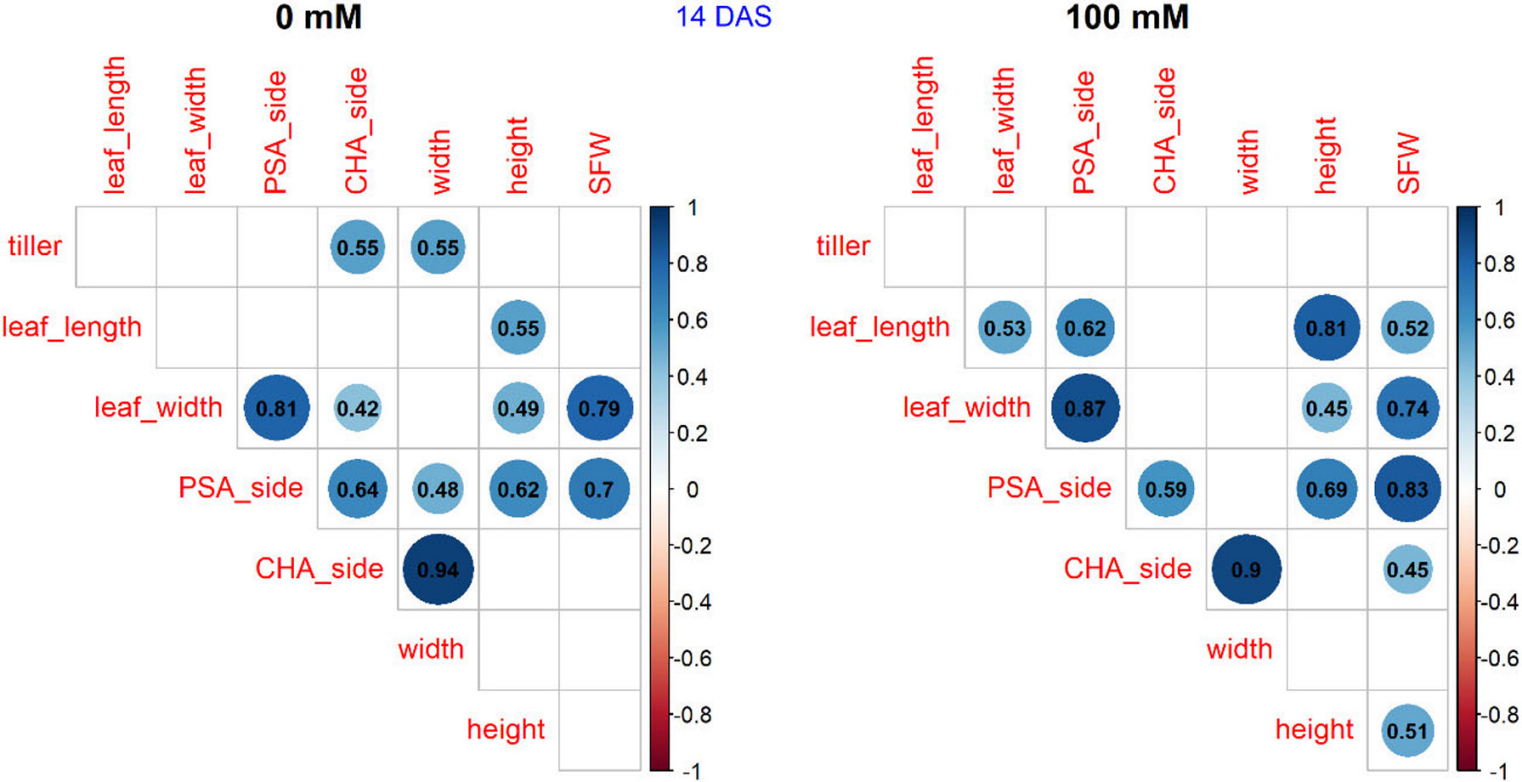
Correlation between projected shoot area (PSA), convex hull area (CHA), width, and height at 14 days after sowing (DAS) and shoot fresh weight (SFW) at 45 DAS under control (left) and under salt treatment (right). Color is significant (*p* < .05); blank is not significant (*p* < .05).

The projected shoot area (PSA) at 14 DAS was strongly correlated to shoot fresh weight (SFW) at 45 DAS under both control and salt treatment, *r* = .70 and .83, respectively (Figure 5). Under salt stress, the convex hull area (CHA) and height were also significantly associated with SFW, while no significant correlations occurred under control conditions.

At 20, 27, 34, and 40 DAS, PSA – the side view and PSA —top view were strongly correlated with fresh shoot weight under both treatments (Figures S14–S17). CHA—side view and CHA—top view were also positively associated with fresh shoot weight under salt treatment at all growth stages. In the control, CHA—top view was positively correlated with fresh shoot weight at all growth stages, while CHA—side view was not positively correlated with fresh shoot weight at 27 and 34 DAS.

At 14 DAS, tiller number was not correlated with fresh shoot weight under both conditions (Figure 5), while a positive correlation occurred at all other growth stages under both treatments (Figures S14–S17). Leaf width was positively correlated with fresh shoot weight at 14, 20, 27 DAS under both treatments (Figure 5; Figures S14 and S15). There was no significant correlation between leaf width and fresh shoot weight at 34 DAS, in the control and at 40 DAS under salt treatment (Figures S16 and S17). At all growth stages, leaf length was not correlated with fresh shoot weight in the control (Figure 5; Figures S14–S17). With salt treatment, fresh shoot weight was positively correlated with leaf length at 14 DAS (Figure 5) but negatively correlated at 34 DAS (Figure S16). There was no significant correlation between leaf length and fresh shoot weight under salt treatment at 20, 27, and 40 DAS (Figures S14, S15 and S17).

In both the control and salt treatment, plant height was not correlated with fresh shoot weight at 20, 27, 34, and 40 DAS (Figures S14–S17). Only at 14 DAS do we see a positive correlation between plant height and fresh shoot weight under salt treatment, while no correlation was observed in the control (Figure 5). Plant width was positively correlated with shoot fresh weight at 20, 34, and 40 DAS under both control and salt treatment (Figures S14, S16 and S17), while no correlation between these parameters was observed at 14 DAS, in both control and salt treatment (Figure 5).

### 3.4 Effects of salt treatment on gas exchange and fluorescence

A three‐way ANOVA showed that genotype and salt treatment significantly (*p* < .001) impacted on the photosynthetic rate (Pn), stomatal conductance (Cond), intracellular CO_2_ concentration (Ci), and transpiration rate (E), while growth stage had no significant effect on the photosynthetic parameters (Table 2). No significant G × T, G × GS, and T × GS interactions but significant (*p* < .05) G × T × GS (genotype × treatment × growth stage) interaction was found for photosynthetic rate (Table 2). Further simple interaction analysis found significant G x T interactions at 42 DAS but not at 35 DAS. Simple effect of genotype for each treatment at 42 DAS was analyzed and genotype significantly affected the photosynthetic rate in both control and treatment (Table S5).

**TABLE 2:** Effect of genotype, salt treatment, and growth stage on photosynthetic rate (Pn), stomatal conductance (Cond), intercellular CO_2_ concentration (Ci), and transpiration rate (E). Two salt levels (0 and 100 mM NaCl) were applied for plants at 14 days after sowing (DAS). Gas exchange measurements were taken at 35 and 42 DAS.

Source	Pn		Cond		Ci		*E*	
	df	*F* ratio	df	*F* ratio	df	*F* ratio	df	*F* ratio
Source								
Genotype (G)	5	16.44***	5	18.33***	5	8.70***	5	18.87***
Treatment (T)	1	77.65***	1	182.12***	1	191.36***	1	199.67***
Growth stage (GS)	1	0.41 ns	1	0.00 ns	1	1.14 ns	1	0.00 ns
G × T	5	1.58 ns	5	6.16***	5	1.72 ns	5	5.49***
G × GS	5	1.42 ns	5	0.71 ns	5	0.44 ns	5	0.68 ns
T × GS	1	0.05 ns	1	0.03 ns	1	0.34 ns	1	0.05 ns
G × T × GS	5	3.31*	5	1.15 ns	5	0.58 ns	5	1.18 ns
Error	1	68	1		1		1	

*Note*: The *F* ratio is from a three‐way ANOVA analysis. * and *** significant at *p* < .05 and *p* < .001, respectively.

Abbreviation: ns, not significant.

**FIGURE 6:**
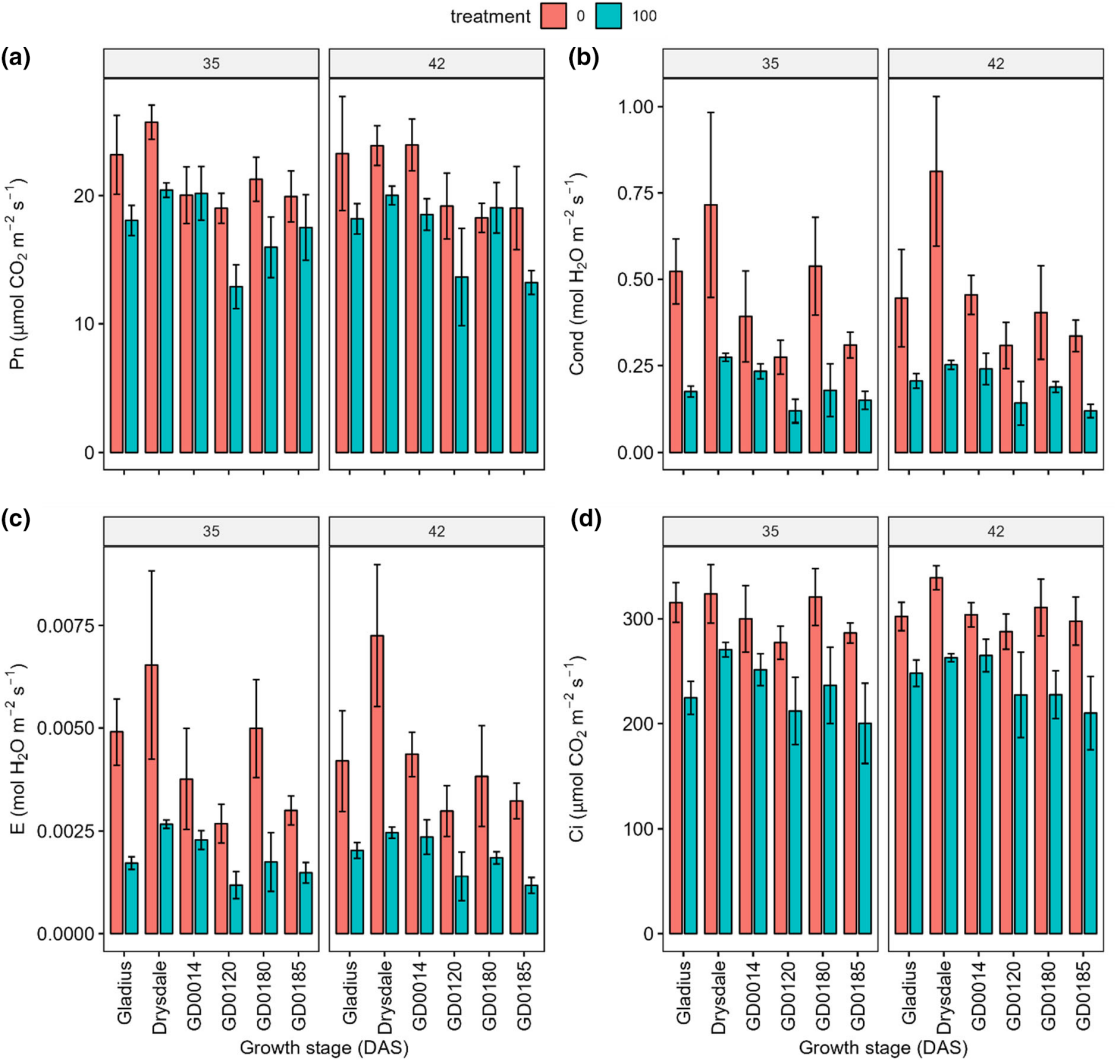
Effect of salt treatment on photosynthetic rate (Pn) (a), stomatal conductance (Cond) (b), transpiration rate (e) (c), and intercellular CO_2_ concentration (ci) (d) of six wheat genotypes at 35 and 42 days after sowing (DAS). Data represent the mean and standard deviation of four replicates. Treatment: 0 mM and 100 mM NaCl.

Although a G × T × GS interaction occurred, the interaction analysis chart showed all genotypes had greater photosynthetic rate in the control when compared to the salt treatment (Figure S18). The main contrast analysis of salt treatment indicated that salt treatment was reduced by 19.2% in photosynthetic rate when compared to the control (21.25 ± 3.16 μmol CO_2_ m^−2^ s^−1^) (Figure 6a; Tables S5 and S9). Simple contrast analysis between genotypes at 35 DAS showed that Drysdale had greatest photosynthetic rate (23.08 ± 3.04 μmol CO_2_ m^−2^ s^−1^) and this was significantly higher than in GD0120 (15.95 ± 3.54 μmol CO_2_ m^−2^ s^−1^), GD0180 (18.62 ± 3.42 μmol CO_2_ m^−2^ s^−1^), and GD0185 (18.72 ± 2.49 μmol CO_2_ m^−2^ s^−1^). Gladius (20.62 ± 3.49 μmol CO_2_ m^−2^ s^−1^) also had a high photosynthetic rate and was significantly greater than in GD0120 (Tables S5 and S9).

A significant G × T interaction was found for the photosynthetic rate at 42 DAS and genotype had a significant effect on this parameter at each salt treatment, therefore simple contrasts between genotypes were analyzed. The results showed that in the control, photosynthetic rate in Gladius (23.28 ± 4.44 μmol CO_2_ m^−2^ s^−1^), Drysdale (23.9 ± 1.55 μmol CO_2_ m^−2^ s^−1^), and GD0014 (23.95 ± 2.01 μmol CO_2_ m^−2^ s^−1^) was significantly higher than in GD0180 (18.26 ± 1.13 μmol CO_2_ m^−2^ s^−1^) (Figure 6a; Tables S5 and S9). GD0140 also showed significantly higher photosynthetic rate when compared to GD0120 (19.18 ± 2.57 μmol CO_2_ m^−2^ s^−1^) and GD0185 (19.02 ± 3.25 μmol CO_2_ m^−2^ s^−1^). Under salt treatment, at 42 DAS, GD0185 (13.21 ± 0.93 μmol CO_2_ m^−2^ s^−1^) had the lowest photosynthetic rate and significantly lower than in Gladius (18.19 ± 1.18 μmol CO_2_ m^−2^ s^−1^) and GD0014 (18.52 ± 1.23 μmol CO_2_ m^−2^ s^−1^) (Figure 6a; Tables S5 and S9).

Genotype and salt treatment significantly (*p* < .001) affected stomatal conductance (Cond), while growth stage had no significant effect on this index. No significant G x GS, T x GS and G x T x GS interactions were observed for stomatal conductance, but a significant G x T interaction occurred (Table 2; Figure S19). The main effect contrast analysis between treatments showed that salt treatment reduced stomatal conductance by 57.9% when compared to the control (0.45 ± 0.19 mol H_2_O m^−2^ s^−1^) (Table S9). Simple effect analysis for genotype at each salt treatment found genotype had no significant effect on stomatal conductance under salt treatment, while having a significant impact in the control (Table S9). Further simple contrast analysis showed that in the control, Drysdale had the highest stomatal conductance (0.76 ± 0.22 mol H_2_O m^−2^ s^−1^), significantly higher than in all other genotypes. In the control, GD0120 (0.29 ± 0.06 mol H_2_O m^−2^ s^−1^) and GD0185 (0.32 ± 0.04 mol H_2_O m^−2^ s^−1^) showed the lowest stomatal conductance and significantly lower than that in Gladius (0.48 ± 0.12 mol H_2_O m^−2^ s^−1^) (Figure 6b; Tables S6 and S9). Similar to stomatal conductance, the effect of genotype and salt treatment on transpiration rate (E) had a similar pattern (Figure 6c; Table 2; Table S8; Figure S20). Genotype and salt treatment also significantly affected the transpiration rate. A significant G × T interaction occurred for this parameter but no G × GS, T × GS, and G × T × GS interactions were found. Further simple analysis and simple effect contrasts also showed a similar pattern to stomatal conductance.

Intercellular CO_2_ concentration was also significantly (*p* < .001) affected by genotype and salt treatment, while it was not significantly impacted by growth stage. No significant G × T, G × GS, T × GS, and G × T × GS interactions were found for this parameter (Table 2; Figure S21). Main contrast analysis showed that salt treatment significantly decreased intercellular CO_2_ concentration by 22.9%, when compared to the control (304.39 ± 24.44 μmol CO_2_ m^−2^ s^−1^) (Figure 6d; Table S9). Drysdale had the highest intercellular CO_2_ concentration (299.14 ± 36.89 μmol CO_2_ m^−2^ s^−1^) and this was significantly greater than in GD0120 (251.16 ± 41.8 μmol CO_2_ m^−2^ s^−1^) and GD0185 (248.67 ± 52.08 μmol CO_2_ m^−2^ s^−1^) (Figure 6d; Tables S7 and S9). GD0185 showed the lowest intercellular CO_2_ concentration and significantly lower than in GD014 (280.1 ± 29.37 μmol CO_2_ m^−2^ s^−1^) and GD180 (273.94 ± 50.64 μmol CO_2_ m^−2^ s^−1^).

OJIP measurements showed that genotype and salt treatment had no significant effects on Fv/Fm, Sm, N, Phi_Po, Phi_Eo, Phi_Do, Phi_Pav, and ABS/RC (Table S10). However, salt treatment significantly impacted on Mo, Psi_o, Pi_Abs, and genotype had significant effects on Ss, TRo/RC, ETo/RC, and DIo/RC (Table S10). No significant interactions were observed for all OJIP parameters (Table S10).

Further post hoc analysis indicated that salt treatment reduced Mo (0.65 ± 0.044) compared to the control (0.68 ± 0.056). However, the treatment increased Phi_Po and Pi_Abs, 0.64 ± 0.013 and 4.05 ± 0.456, respectively in comparison with the control, 0.63 ± 0.019, 3.73 ± 0.521 (Table S10).

Gladius (0.57 ± 0.029) showed significantly higher Ss than GD0014 (0.54 ± 0.02), while TRo/RC was significantly lower in Gladius (1.74 ± 0.085) than in GD0014 (1.86 ± 0.076). Similar to TRo/RC, Gladius had significantly lower ETo/RC (1.09 ± 0.045) than GD0014 (1.16 ± 0.061) and GD0185 (1.15 ± 0.032). DIo/RC in Gladius (0.35 ± 0.019) was also significantly lower than in GD0014 (0.4 ± 0.026) (Table S10).

### 3.5 Correlation between photosynthetic traits and shoot fresh weight, leaf water content, and greenness

**FIGURE 7:**
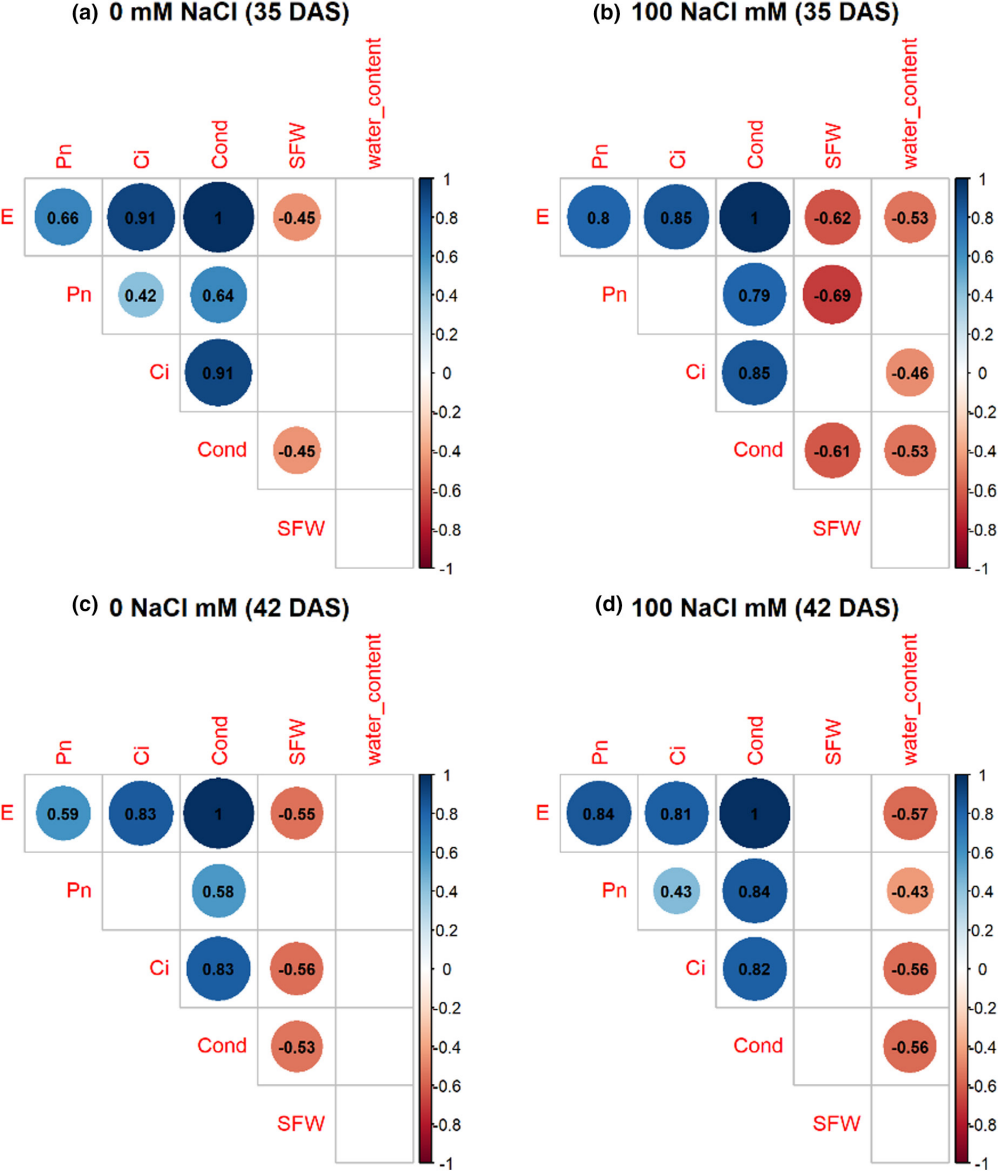
Correlation between photosynthetic rate (Pn), stomatal conductance (Cond), transpiration rate (E), and intercellular CO_2_ concentration (Ci) at 35 and 42 days after sowing (DAS) with shoot fresh weight (SFW) and leaf water content at 45 DAS under control and under salt treatment. Color is significant (*p* < .05); blank is not significant (*p* < .05). (a) 0 mM NaCl at 35 DAS, (b) 100 mM NaCl at 35 DAS, (c) 0 mM NaCl at 42 DAS, (d) 100 mM NaCl at 42 DAS.

With the application of salt, the photosynthetic rate, stomatal conductance, and transpiration rate at 35 DAS were negatively correlated with fresh shoot weight at 45 DAS, while no significant correlation occurred at 42 DAS (Figure 7b,d). Gas exchange parameters had no significant correlation with leaf water content under the control at both growth stages (Figure 7a,c), while under salt treatment, stomatal conductance, intercellular CO_2_ concentration, and transpiration rate were negatively correlated with leaf water content at both growth stages (Figure 7b,d). There was no correlation between shoot fresh weight and leaf water content under both control and treatment (Figure 7a–d).

There were no significant correlations between OJIP parameters and shoot fresh weight and greenness under salt treatment (Figure S22). Similar results were found under the control, except for Vi where this parameter was positively correlated with shoot fresh weight (Figure S22).

### 3.6 Salt treatment effect and genotypic variation in Na, K, and other minerals concentration in leaves

**FIGURE 8:**
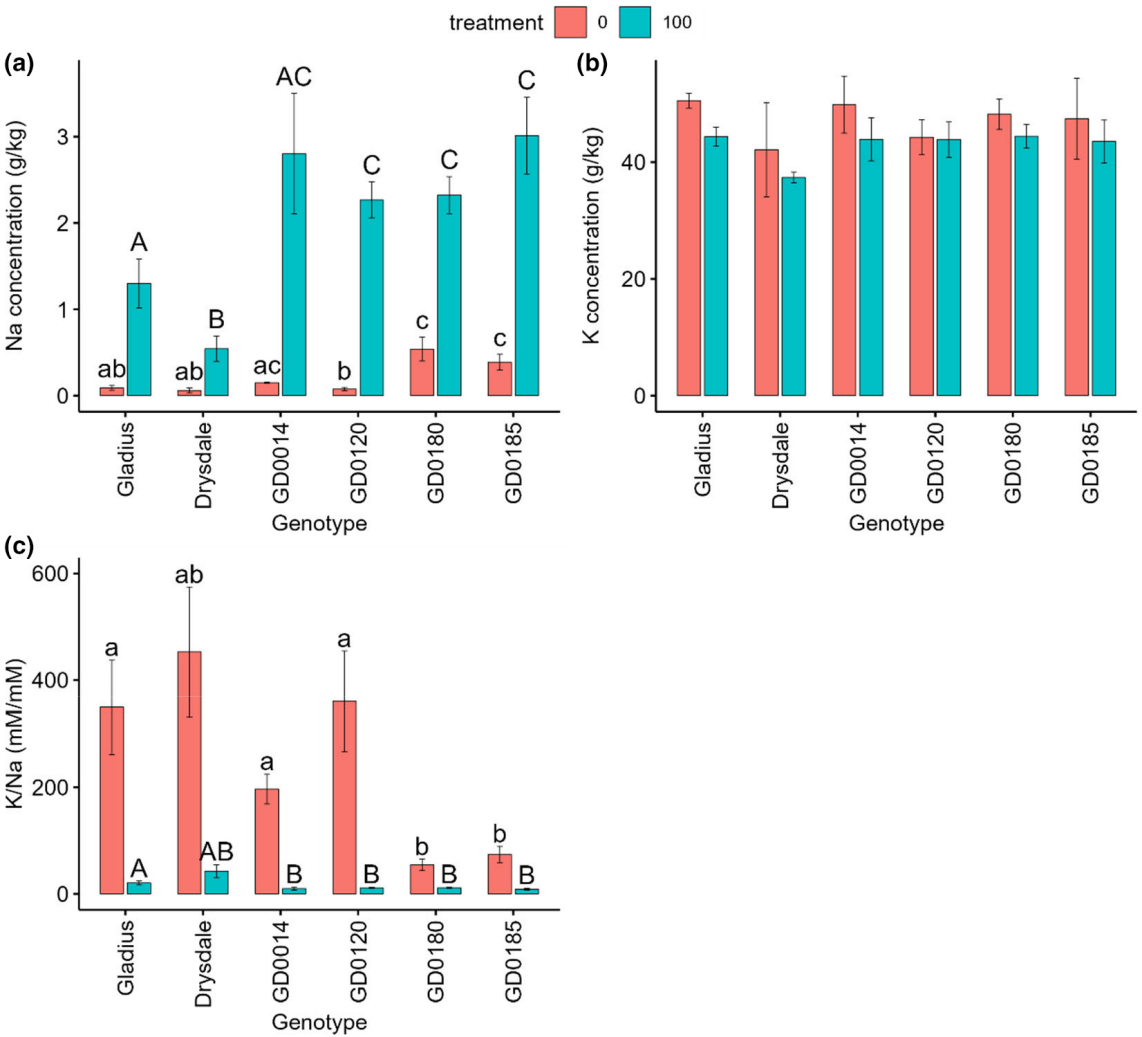
Effect of salt treatment on leaf Na (a), K concentration (b) and K/Na ratio (c) in six wheat genotypes at 45 days after sowing (DAS). Data represent the mean and standard deviation of four replicates. Treatment: 0 mM and 100 mM NaCl. Different letters showed significant differences between genotypes at each salt treatment.

Two‐way ANOVA analysis showed that genotype and salt treatment had a significant (*p* < .001) effect on Na concentration. A significant (*p* < .001) G × T interaction for this parameter was also observed (Table S11; Figure S23). Simple effect analysis indicated that genotype significantly affected Na concentration under both salt treatment and control. Simple contrast analysis between genotypes under salt treatment showed that GD185 had the highest leaf Na concentration (3.01 ± 0.44 g/kg) and significantly higher than in Gladius (1.3 ± 0.28 g/kg) and Drysdale (0.54 ± 0.15 g/kg) (Figure 8a; Table S11). GD0120 and GD0180 also showed high leaf Na concentrations and were significantly greater than in Gladius and Drysdale. Although a significant G x T interaction occurred for leaf Na concentration, this concentration under treatment was higher than under control for all other genotypes (Figure 8a). Thus, the main contrast analysis between treatments indicated that salt treatment increased 9.5 times compared to control (Table S11).

Both genotype and salt treatment significantly affected leaf K concentration and no significant G x T interaction occurred for this parameter (Table S11). The main effect analysis of salt treatment showed that salt treatment (47.3 ± 5.2 g/kg) significantly decreased leaf K concentration when compared to the control (43.1 ± 3.4 g/kg) (Figure 8b, Table S11).

Both genotype and salt treatment significantly affected the K/Na ratio in leaf and a significant G x T interaction for this parameter was also observed (Tables S11 and S24). Simple effect analysis showed that genotype had a significant effect on K‐to‐Na ratio under both treatments (Figure 8c).

Regarding other minerals or elements, genotype had no effect on leaf P concentration, while salt treatment had a significant effect on this parameter (Table S11). No significant G x T interaction occurred for leaf P concentration. The main effect analysis showed that salt treatment (6.51 ± 0.74 g/kg) increased leaf P concentration compared to the control (6.04 ± 0.73 g/kg) (Table S11).

Genotype and salt treatment significantly affected the leaf B, Mg, and S concentration and no significant G x T interactions for these concentrations were found (Table S11). Salt treatment reduced leaf B concentration 1.3 folds, while salt treatment increased leaf Mg concentration 1.3 folds (Table S11). Salt treatment also significantly increased leaf Mn and Zn concentrations (Table S11).

### 3.7 Salt treatment effect on the phloem sucrose level of two wheat genotypes, gladius, and Drysdale

**FIGURE 9:**
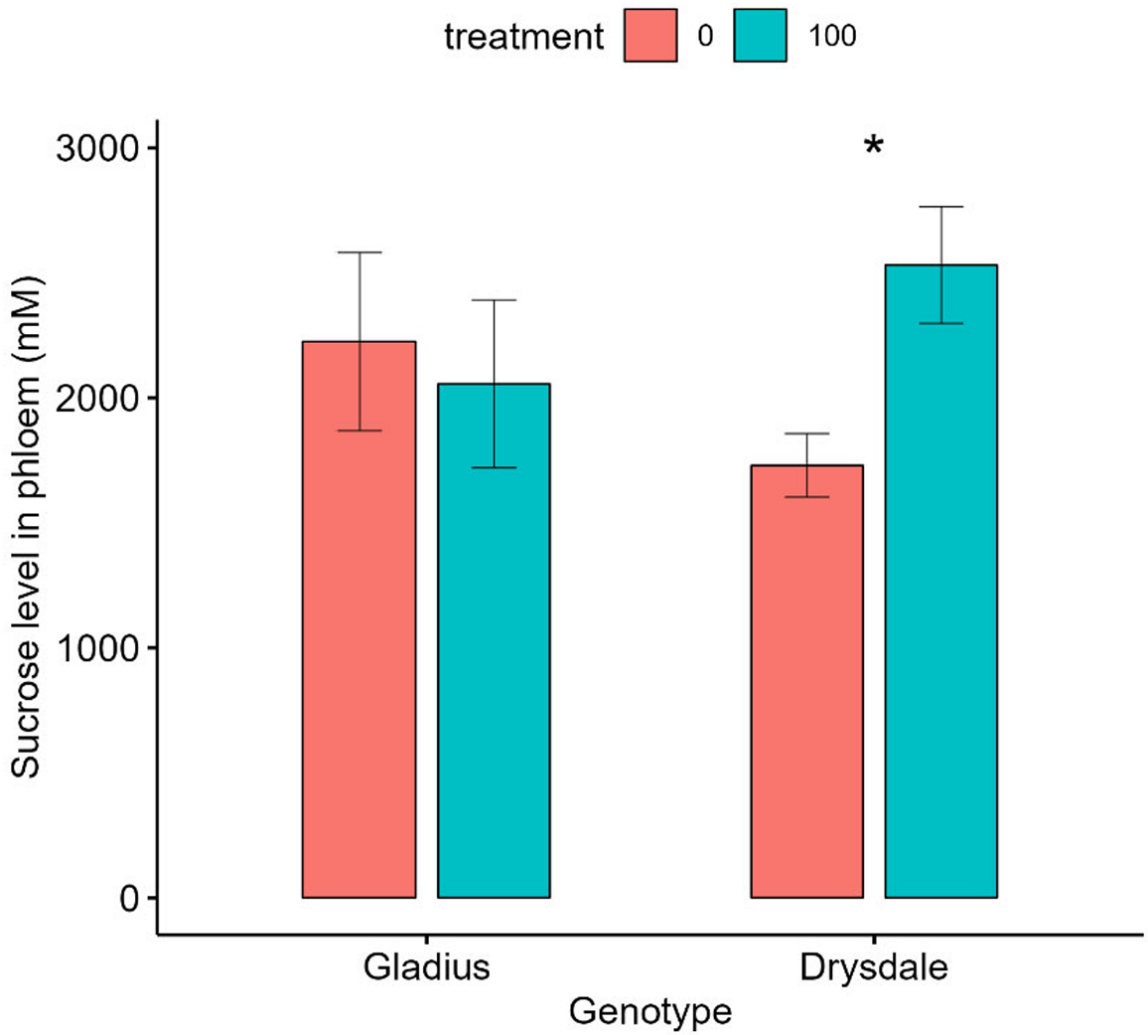
Sucrose concentration in the phloem of two wheat genotypes under different salt treatments. Data represent the mean and standard deviation of three replicates. Treatment: 0 mM and 100 mM NaCl. Star (*) showed significant differences between treatments within each genotype.

The phloem collection showed that the phloem exudate in Drysdale under salt treatment was more viscous than that in the Gladius. Two‐way ANOVA analysis showed a significant (*p* < .05) interaction between genotype and salt treatment for phloem sucrose level. Further contrast analysis between treatments for each genotype indicated salt treatment had no significant effect on the sucrose level of the phloem for Gladius, while salt treatment significantly increased 46.3% phloem sucrose level for Drysdale compared to control (1730 ± 127 mM) (Figure 9).

## 4 DISCUSSION

### 4.1 Projected shoot area at early growth stages can be a potential indicator for salt tolerance screening

Phenotyping by imaging is a useful tool since it is a non‐destructive method and can be used to analyze shoot parameters at any stage of plant growth (Hairmansis et al., 2014; Moghimi et al., 2018). This is important because the response of plants to stress varies between growth stages (Armoniené et al., 2018). Our study also showed that the six wheat genotypes responded differently at different growth stages. Indeed, GD0120 indicated an earlier response to salt treatment, when compared to other genotypes (Figures 2 and 3). The investigation of shoot parameters responding to salt treatment at different growth stages can provide useful information to elucidate mechanisms of salt tolerance.

Shoot parameters were analyzed along the time course of growth to evaluate if these parameters can be used to identify tolerant genotypes. Plantcv, a Python‐based method was used for the analysis of shoot parameters. This is a fast and accurate method for analysis (Armoniené et al., 2018; Gehan et al., 2017). Shoot projected area (PSA) of both side view and top view at 20, 27, 34, and 40 DAS were strongly (*r* = .79–.96) correlated with fresh shoot weight at 45 DAS under both conditions (control and treatment). Shoot projected area – side view at 14 DAS was also strongly correlated with fresh shoot weight under control (*r* = .7) and under salt treatment (*r* = .83) (Figure 5). Convex hull area (CHA)—top view at 20, 27, 34, and 40 DAS were also positively correlated with fresh shoot weight at 45 DAS under both control (*r* = .49–.82) and salt treatment (*r* = .52–.78) (Figure 5). Thus, the shoot projected area and convex hull area at the early stages can be used to screen salt tolerance wheat genotypes.

Gladius and GD0120 showed higher fresh shoot weight at 45 DAS under salt treatment, while Drysdale showed the lowest fresh shoot weight (Figure 1). These results agree with the study by Shamaya (2014) where Drysdale showed lower shoot mass than Gladius under salt stress. Gladius (0.63) also showed a greater fresh shoot weight ratio between treatment and control than in Drysdale (0.59) (Table 1). Shamaya (2014) found this ratio in Gladius was higher in Drysdale in most of the soil types used to test the response of wheat to salt treatment.

### 4.2 Photosynthesis and salt tolerance

Salinity not only negatively affects plant cell functioning by reducing cell division and inducing cell swelling (van Zelm et al., 2020), it also alters the osmotic pressure of the soil solution (Dhansu et al., 2022; Munns & Tester, 2008; Preet et al., 2024). Thus, salinity has a negative impact on photosynthetic traits since plants respond to salt by reducing the stomatal aperture to adapt to the osmotic effect of the salt outside the roots (Munns & Tester, 2008). The narrower stomatal aperture limits the CO_2_ diffusion and reduces the photosynthetic rate (Chaves et al., 2008). This study found salt treatment significantly reduced photosynthetic rate by 19.2%, stomatal conductance by 57.9%, and intercellular CO_2_ concentration by 22.9% (Table S9). The effect of salt treatment on transpiration rate was similar to stomatal conductance. Salinity lowers leaf water potential and plants tend to close their stomata to reduce water loss (Masarmi et al., 2023; Orzechowska et al., 2021), and this resulted in a significant drop in stomatal conductance and transpiration rate. A substantial decrease in photosynthetic rate, stomatal conductance, and transpiration rate under salt treatment was also observed in previous studies in wheat (Elhakem, 2020; Saddiq et al., 2021; Zahra et al., 2022). Similar results were also found in barley (Mahlooji et al., 2018), *Reaumuria soongorica* (Yan et al., 2022) and *Avicennia marina* (Dittmann et al., 2022). High salinity reduced intercellular CO_2_ concentration in wheat (Pastuszak et al., 2022) and in other plants such as *Phaseolus vulgaris* L. (Seemann & Critchley, 1985), *Solanum photeinocarpum* (Liao & Xu, 2021).

The high salt concentration increases the osmotic pressure of the soil solution and results in less water being available to plants. This lowers transpiration rate and stomatal conductance. The closure of stomata also reduces the flow of CO_2_ into the leaf and decreases the intercellular CO_2_ concentration. A lower transpiration rate leads to a decrease in leaf water content and therefore drops the rate of photosynthesis. The accumulation of Na^+^ in the cells at high salt concentrations, change the K‐to‐Na ratio, which may affect the photosynthesis process (Sudhir & Murthy, 2004). The reduction in the activity of photosynthetic enzymes such as Rubisco could also reduce the capacity of CO_2_ fixation (Kamal et al., 2012; Ziska et al., 1990).

Drysdale showed the highest intercellular CO_2_ concentration and also had a higher stomatal conductance and transpiration rate (Figure 6b–d; Table S9). However, this genotype had low fresh shoot biomass (Figure 1). Correlation analysis revealed, under control conditions, that the stomatal conductance and transpiration rate at both 35 and 42 DAS were negatively correlated with fresh shoot weight at 45 DAS (Figure 7). The same correlation also occurred under salt treatment at 35 DAS but not at 42 DAS. This indicates that the response of wheat genotypes to salt treatment changed after a longer treatment period. Interestingly, stomatal conductance and transpiration rate were not correlated with leaf water content within the control, while these parameters were negatively correlated with leaf water content under salt treatment at both growth stages. This correlation may link to the closure of stomata. Genotypes with a more efficient mechanism of stomatal closure under salt stress resulted in higher leaf water content while also having a lower transpiration rate and stomatal conductance.

OJIP is the chlorophyll fluorescence kinetic analysis and O stands for “Origin” (minimal fluorescence), P for “peak”, and J and I for inflection point between O and P (Khan et al., 2021; Küpper et al., 2018). OJIP measures changes in chlorophyll fluorescence, quantum yield along the time course of measurement and therefore it can reveal insights into subtle changes of the photosynthesis system (Küpper et al., 2018). This study showed that salt treatment had no significant effect on the O and P stages of the curve. Masarmi et al. (2023) reported that salt treatment had a negative, significant effect on OJIP parameters in a sensitive wheat genotype, while salinity showed no significant effect on these parameters in the tolerant wheat genotypes. Salt treatment also had a more negative effect on the OJIP parameters of the salt‐sensitive genotypes (Ibrahimova et al., 2021; Oyiga et al., 2016). Salt treatment showed negative effects on OJIP such as *F*_v_/*F*_m_ in both salt‐sensitive and salt‐tolerant wheat cultivars (Zw et al., 2016). Variation between studies, including our results would be due to genetic variation or the differences in treatment levels. The higher salt treatment level would have a more negative effect on OJIP parameters. No significant correlation between OJIP parameters with shoot fresh weight was observed under both conditions, except for Vi which was positively correlated with fresh shoot weight in the control (Figure S22). Thus, OJIP appears to not be a good indicator to identify salt‐tolerant wheat under this treatment and growth condition.

### 4.3 Na, K, and K‐to‐Na ratio

Higher levels of salinity in soil cause high leaf Na concentration and this negatively affects the normal growth of plants. Our results showed that salt treatment significantly increased leaf Na concentration, 9.5 times more than the control (Figure 8a; Table S11). In contrast, salt treatment significantly reduced leaf K concentration to 43.1 ± 3.4 g/kg when compared with 47.3 ± 5.2 g/kg of control. Salt treatment also significantly reduced the K‐to‐Na ratio in the leaf about 14.2 times compared to the control (Figure 8c; Table S11). The significant increase of leaf Na concentration under salt treatment would result in this dramatic drop in the K‐to‐Na ratio. A significant increase in leaf Na concentration and a significant decrease in leaf K‐to‐Na ratio under salt treatment were also reported in wheat (El‐Hendawy et al., 2005; Ibrahimova et al., 2021) and in quinoa (Cai & Gao, 2020). Similar to our results, previous studies in wheat also showed salt treatment reduced this leaf K concentration (El‐Hendawy et al., 2005; Ibrahimova et al., 2021). However, salinity increased leaf K concentration in quinoa (Cai & Gao, 2020). This difference could be due to variation between plants.

**FIGURE 10:**
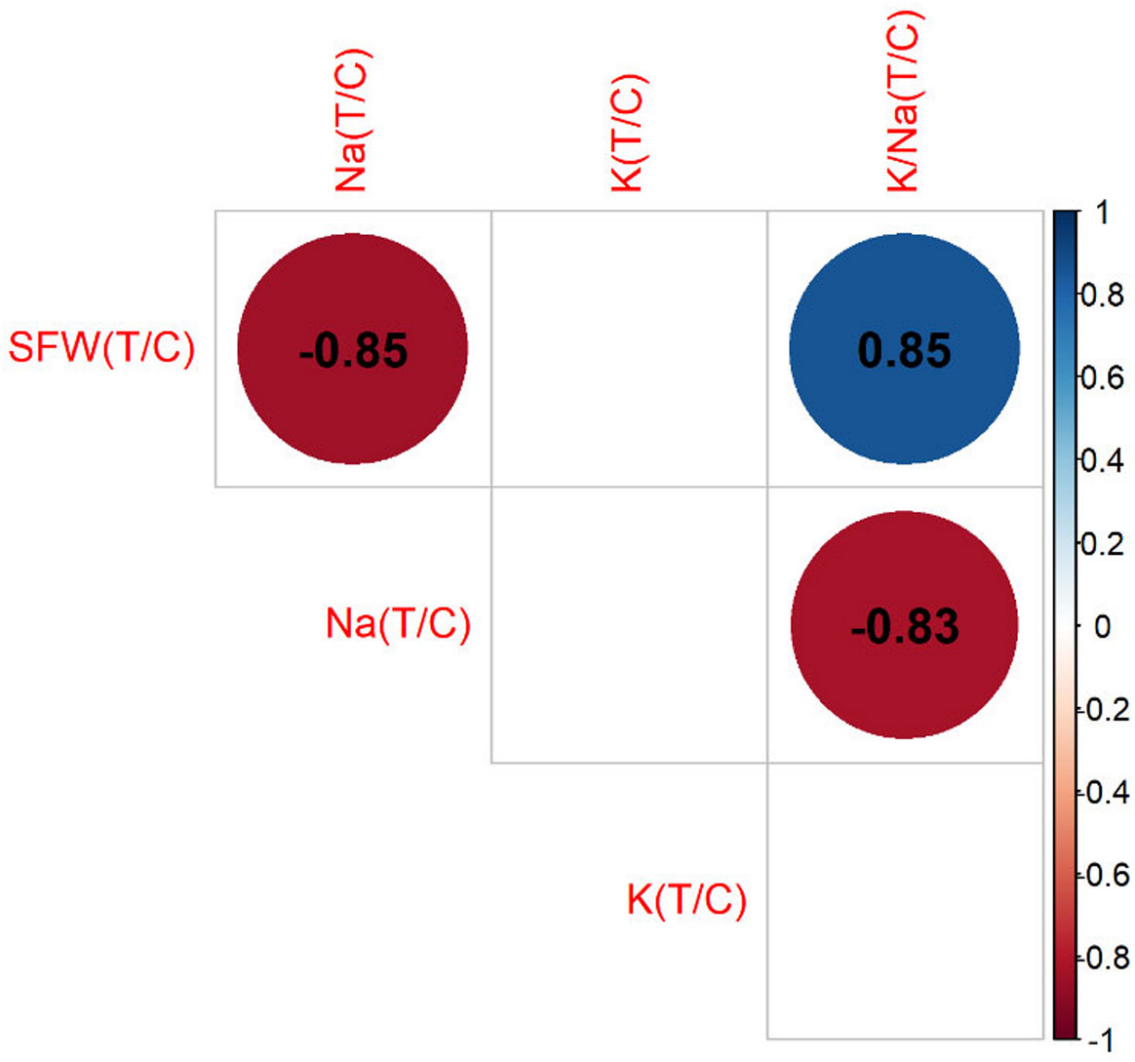
Correlation of relative ratio (treatment/control) between leaf Na, K, and K/Na and fresh shoot weight. T/C represents for treatment/control. Color is significant (*p* < .05); blank is not significant (*p* < .05); SFW, shoot fresh weight.

Correlation analysis showed that the ratio of leaf K between treatment and control was not significantly correlated with the ratio of fresh shoot weight, while a positive correlation occurred in this ratio between the K/Na ratio and fresh shoot weight (*r* = .85) (Figure 10). In contrast, a negative correlation was found between this ratio of Na and the ratio of fresh shoot weight (*r* = −.85) (Figure 10). This indicates that the wheat genotype with a higher salt tolerance index has an elevated ratio of K/Na between the treatment and control. Previous studies showed that the K/Na ratio is considered an indicator for salt tolerance in plants (Chhipa & Lal, 1995; El‐Hendawy et al., 2009; Hussain et al., 2021; Reddy et al., 2017). Enhanced K accumulation was reported to be associated with salt tolerance (Cheng et al., 2015; Shirazi et al., 2005). Thus, the ratio of K/Na between treatment and control can be a potential indicator for screening tolerant wheat genotypes.

Salt stress causes dehydration (Hannachi et al., 2022) and our study showed salt treatment reduced stomatal conductance and transpiration rate (Figure 6b,c). At 42 DAS, Gladius showed greater relative stomatal conductance (0.47) than Drysdale (0.31). This could be a reason why the phloem exudate of Drysdale was more viscous than that of Gladius. However, both Drysdale and Gladius had high photosynthetic rates and Drysdale had a higher relative photosynthetic rate (0.84) than Gladius (0.78). This may indicate that Drysdale is able to naturally produce and load more sucrose into the phloem, but with a lower water potential in the xylem, this results in a concentrating of sucrose in the Drysdale phloem, and subsequent reduced transport of sucrose from source to sink.

The findings presented in this study provide useful approaches to screen tolerant wheat and further investigation of salt treatment mechanisms. As a strong positive correlation exists between projected shoot areas at the early growth stage, with fresh shoot weight present under salt treatment, this parameter can be used to screen tolerant wheat. Under salt treatment, stomatal conductance and transpiration rate at both growth stages were negatively correlated with leaf water content. This reveals that salt tolerance might be related to the mechanism of stomata closure. The ratio of K/Na between treatment and control was strongly correlated with the ratio of fresh shoot weight between treatment and control, thus this ratio could be an indicator for salt tolerance in wheat. The study found that the higher salt tolerant genotype, Gladius is associated with greater maintenance of stomatal conductance and lower sucrose level in the phloem when compared to Drysdale.

## CONFLICT OF INTEREST STATEMENT

The authors declare that the research was conducted in the absence of any commercial or financial relationships that could be construed as a potential conflict of interest.

## Supporting information


Data S1.
