# Supplementary material for: Salt tolerance in wheat is associated with the maintenance of shoot biomass, stomatal conductance, and sucrose in the phloem
Source: Plant Environ Interact. 2024 Sep 11;5(5):e70008. doi: 10.1002/pei3.70008 (PMC11389530; doi:10.1002/pei3.70008)
Supplement: Supplementary file 1 — Data S1. [file PEI3-5-e70008-s001.docx]

**Salt tolerance in wheat is associated with maintenance of shoot biomass, stomatal conductance and sucrose in the phloem**

**Supplementary figures**

***Plant imaging and analysis***


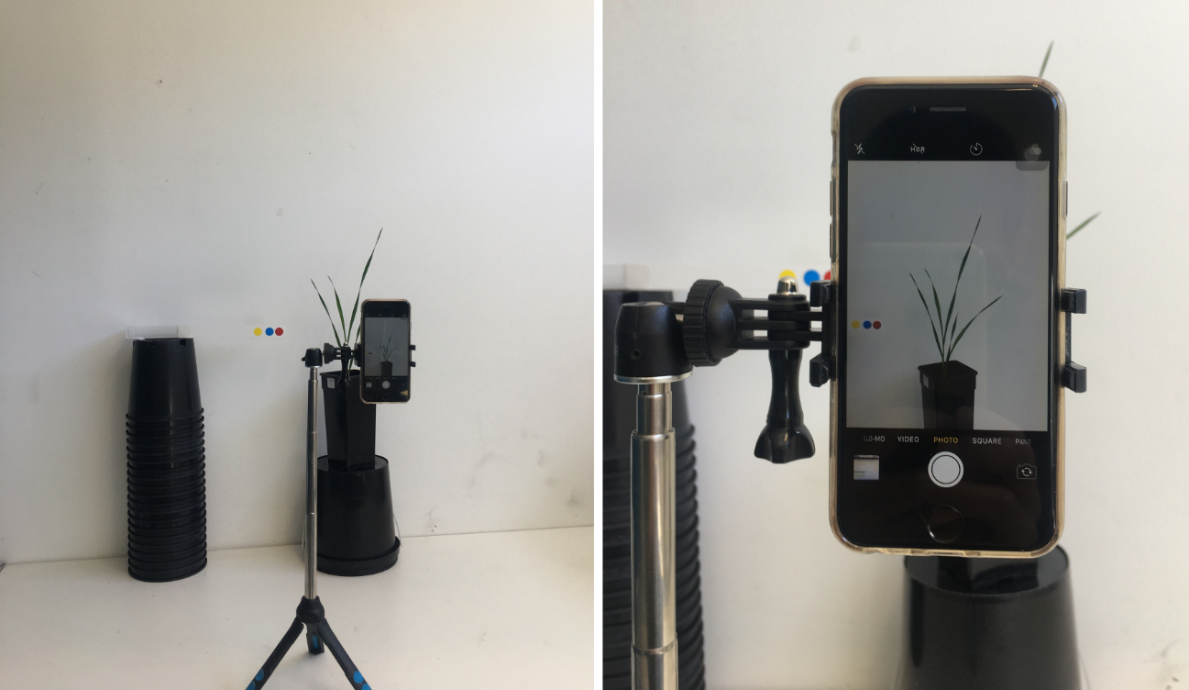


Figure S1. Plant imaging


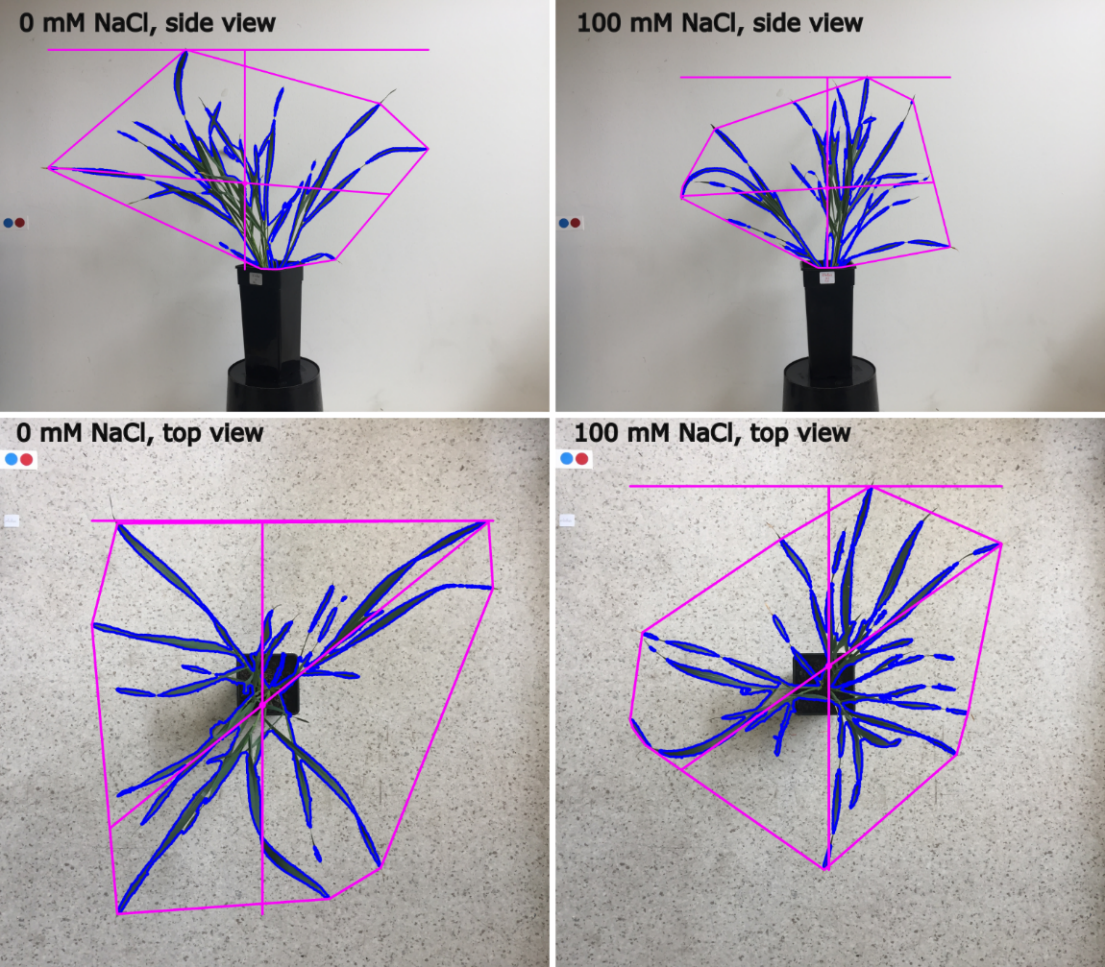


Figure S2. Shoot analysis using Plantcv at 40 days after sowning (DAS). Wheat genotype in these images is Gladius.

***Shoot parameters***


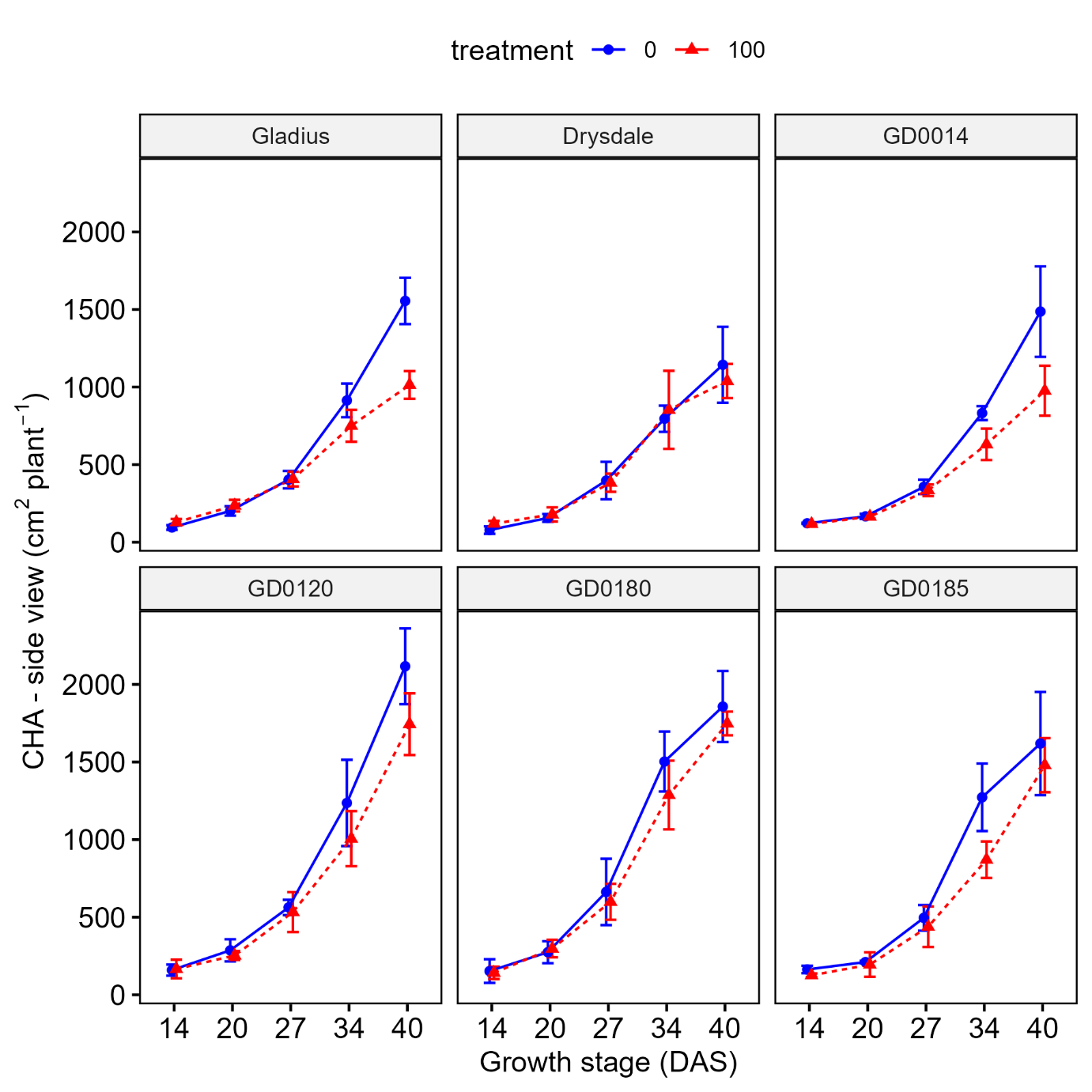


Figure S3. Effect of salt treatment on convex hull area (CHA) - side view of six wheat genotypes at different growth stages. Data represents the mean and standard deviation of four replicates. Treatment: 0 mM and 100 mM NaCl; DAS: days after sowing.


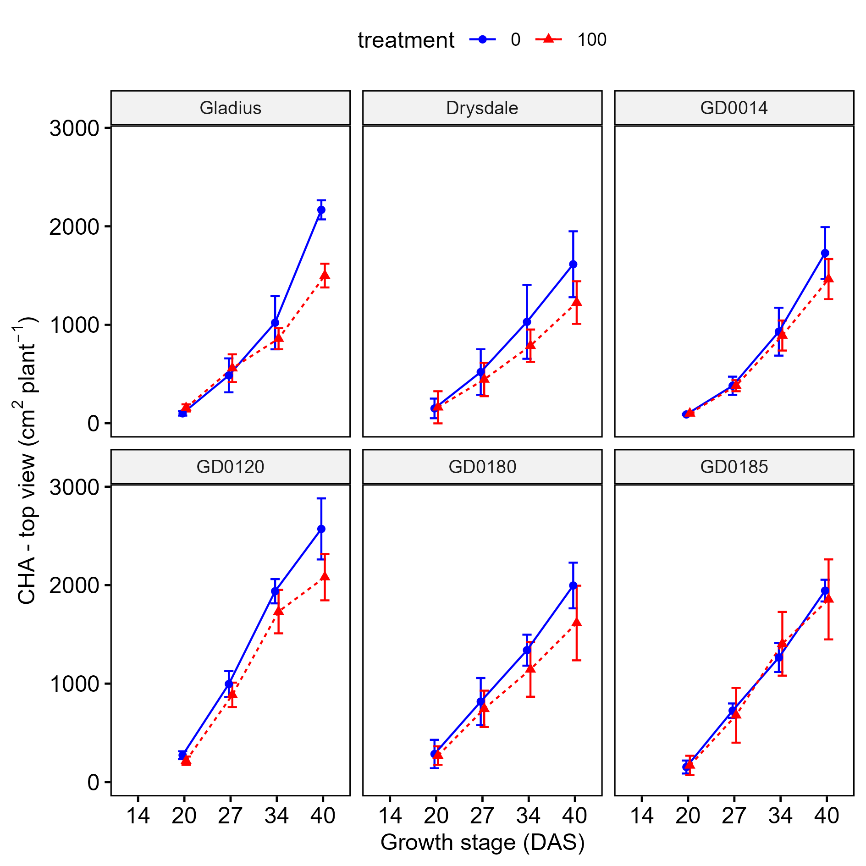


Figure S4. Effect of salt treatment on convex hull area (CHA) - top view of six wheat genotypes at different growth stages. Data represents the mean and standard deviation of four replicates. Treatment: 0 mM and 100 mM NaCl; DAS: days after sowing.


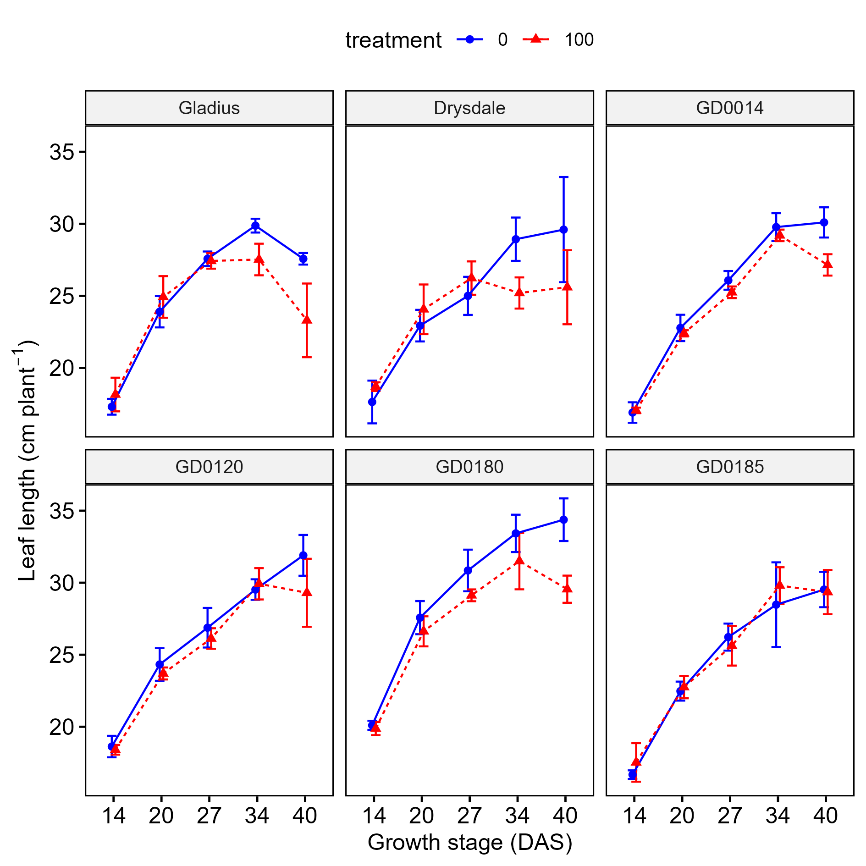


Figure S5. Effect of salt treatment on leaf length of six wheat genotypes at different growth stages. Data represents the mean and standard deviation of four replicates. Treatment: 0 mM and 100 mM NaCl; DAS: days after sowing.


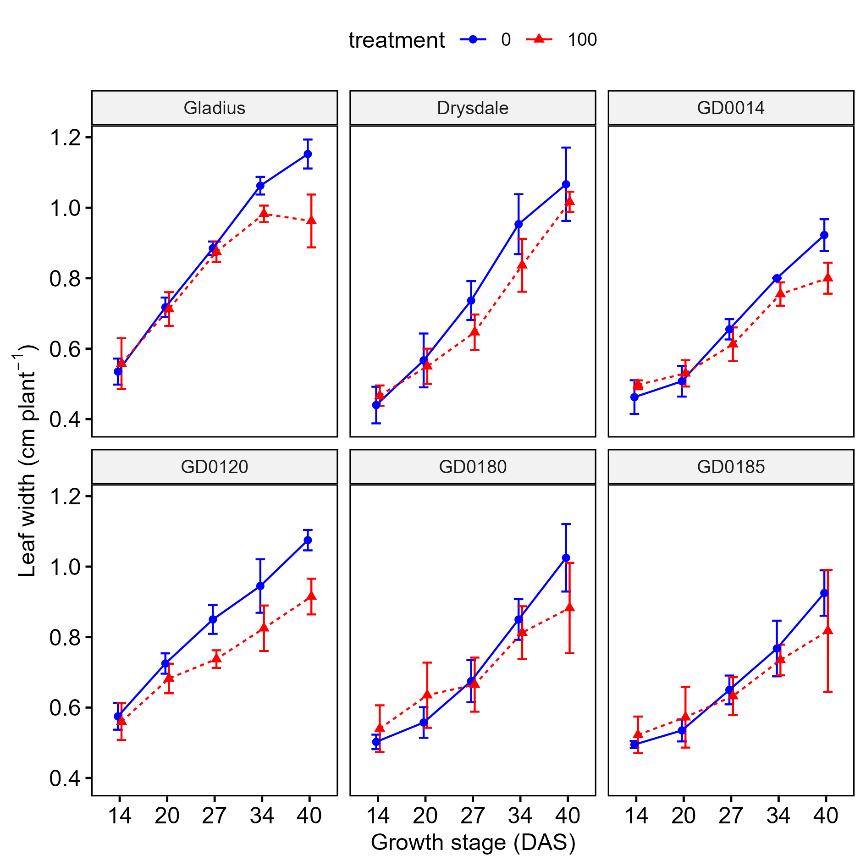


Figure S6. Effect of salt treatment on leaf width of six wheat genotypes at different growth stages. Data represents the mean and standard deviation of four biological replicates. Treatment: 0 mM and 100 mM NaCl; DAS: days after sowing.


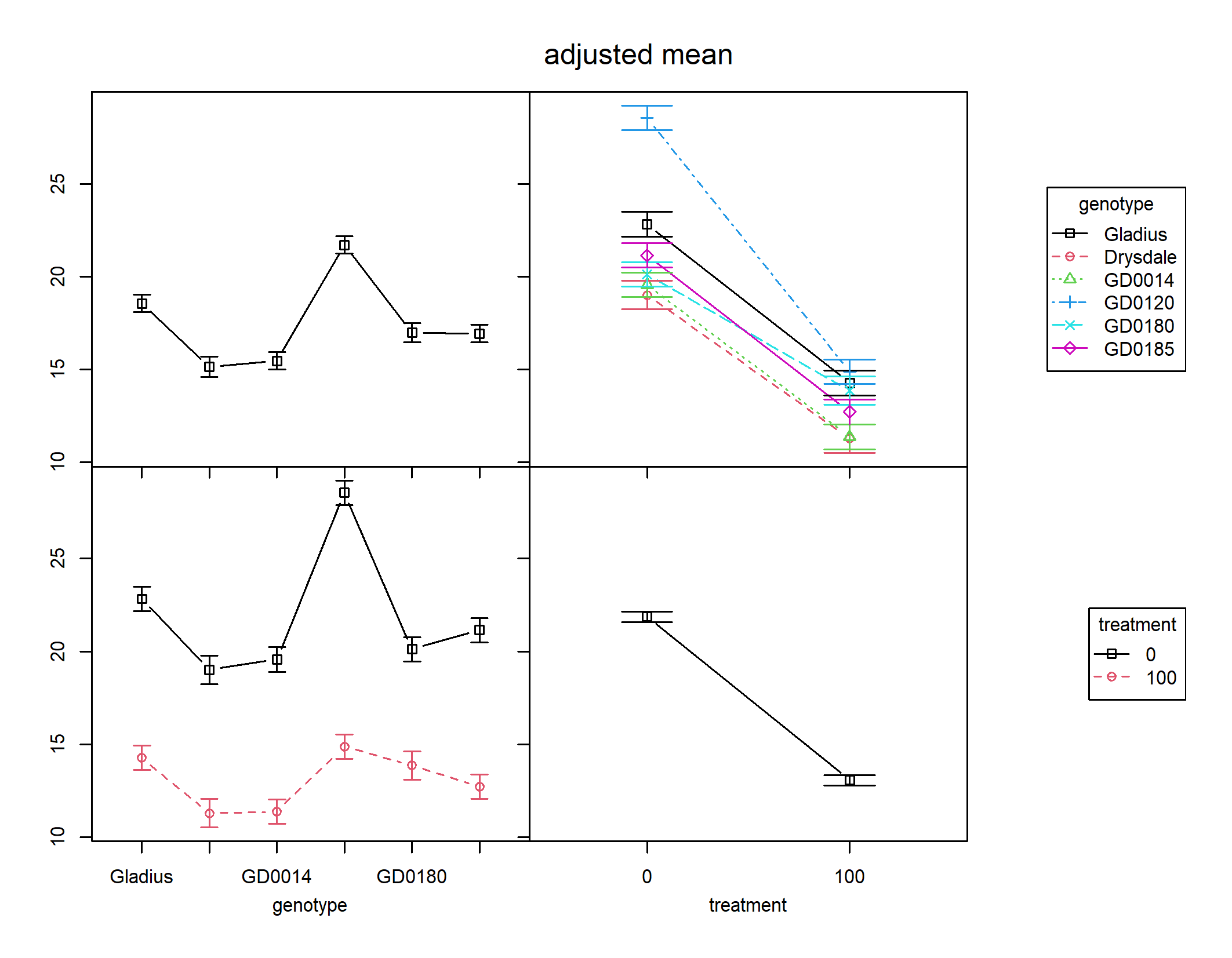


Figure S7. Analysis of genotype, salt treatment and growth stage effects on shoot fresh weight (SFW) using Phia package.


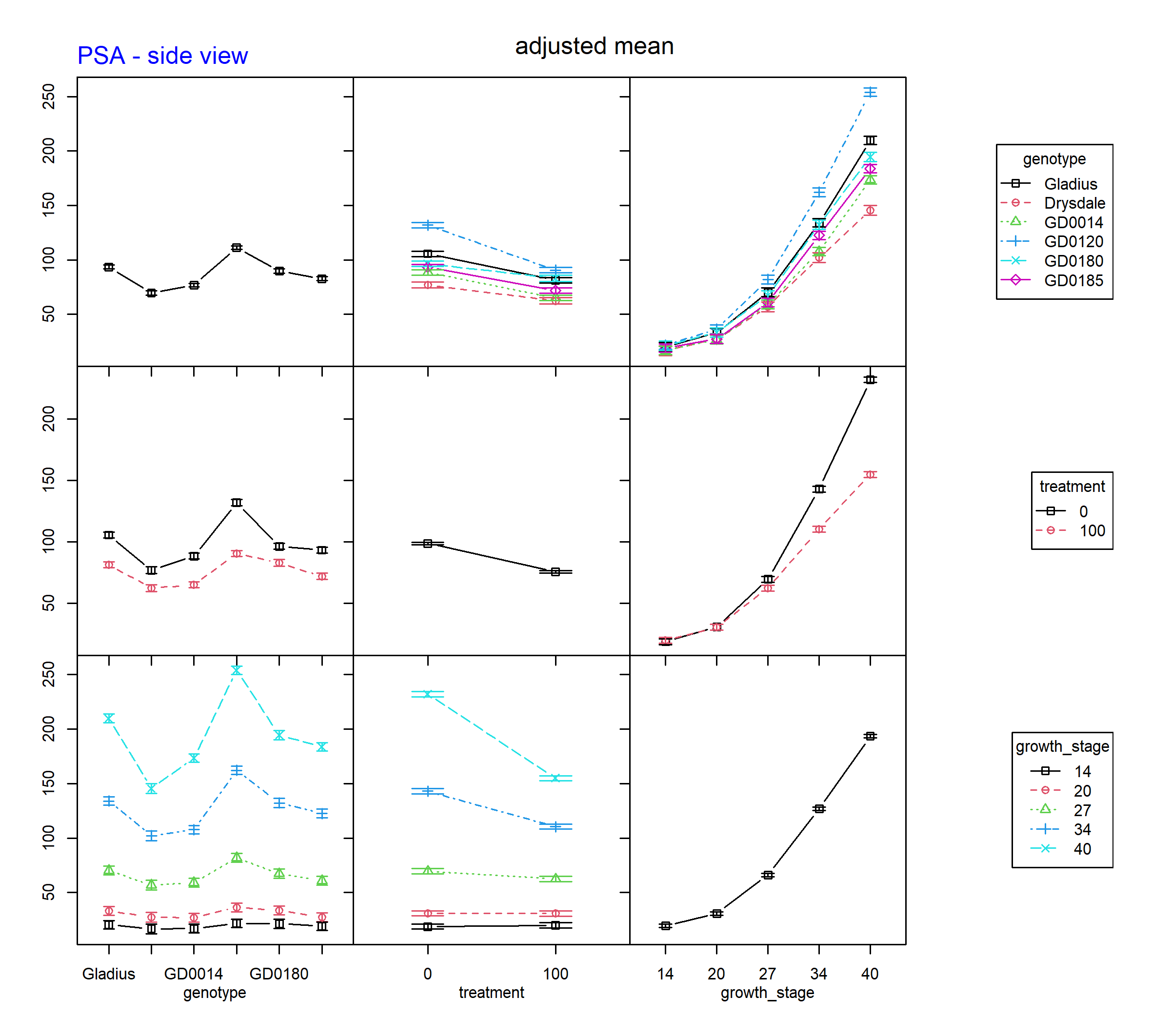


Figure S8. Analysis of genotype, salt treatment and growth stage effects on shoot projected area – side view (PSA – side view) using Phia package.


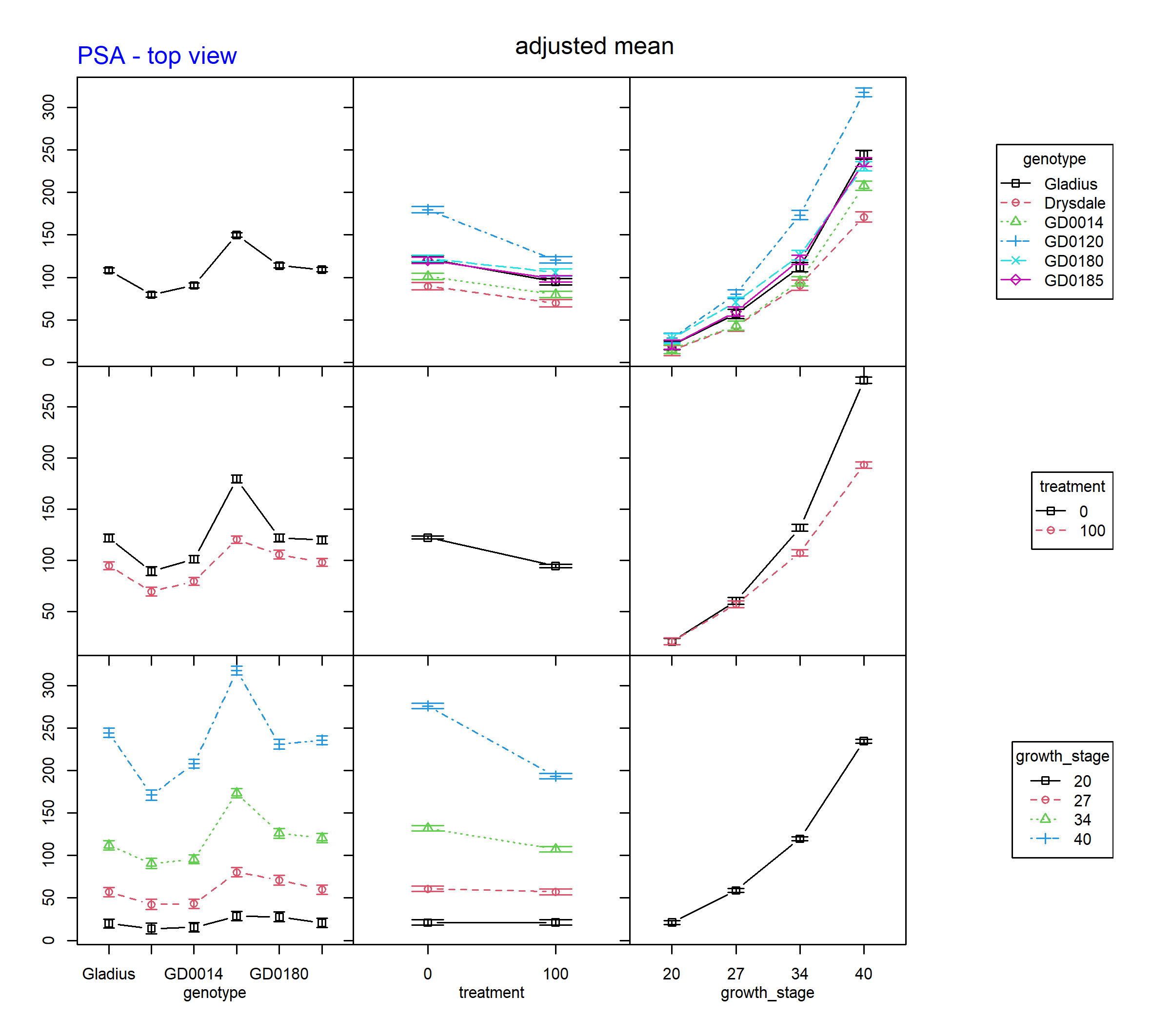


Figure S9. Analysis of genotype, salt treatment and growth stage effects on shoot projected area – top view (PSA – top view) using Phia package.


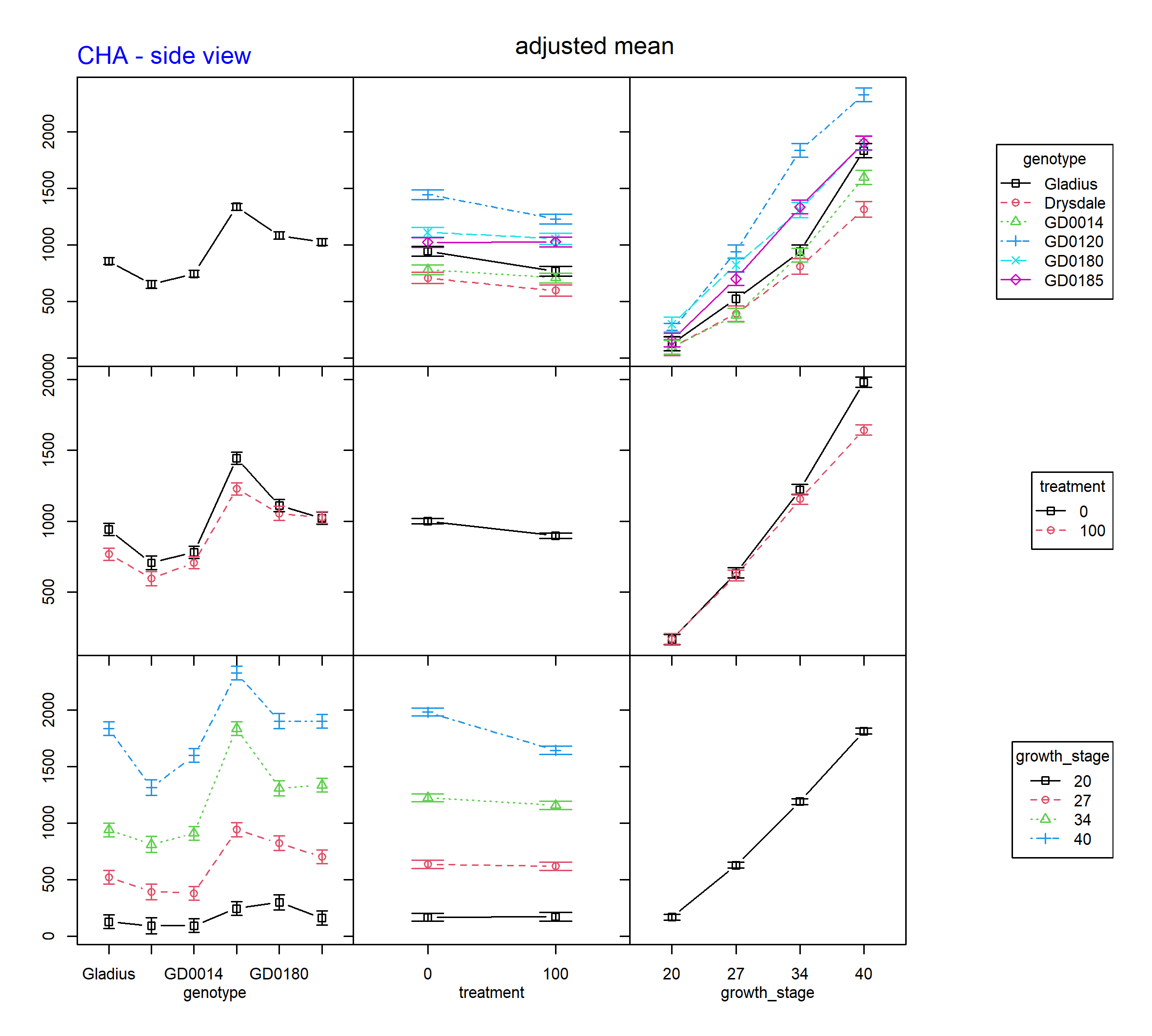


Figure S10. Analysis of genotype, salt treatment and growth stage effects on convex hull area – side view (CHA – side view) using Phia package.


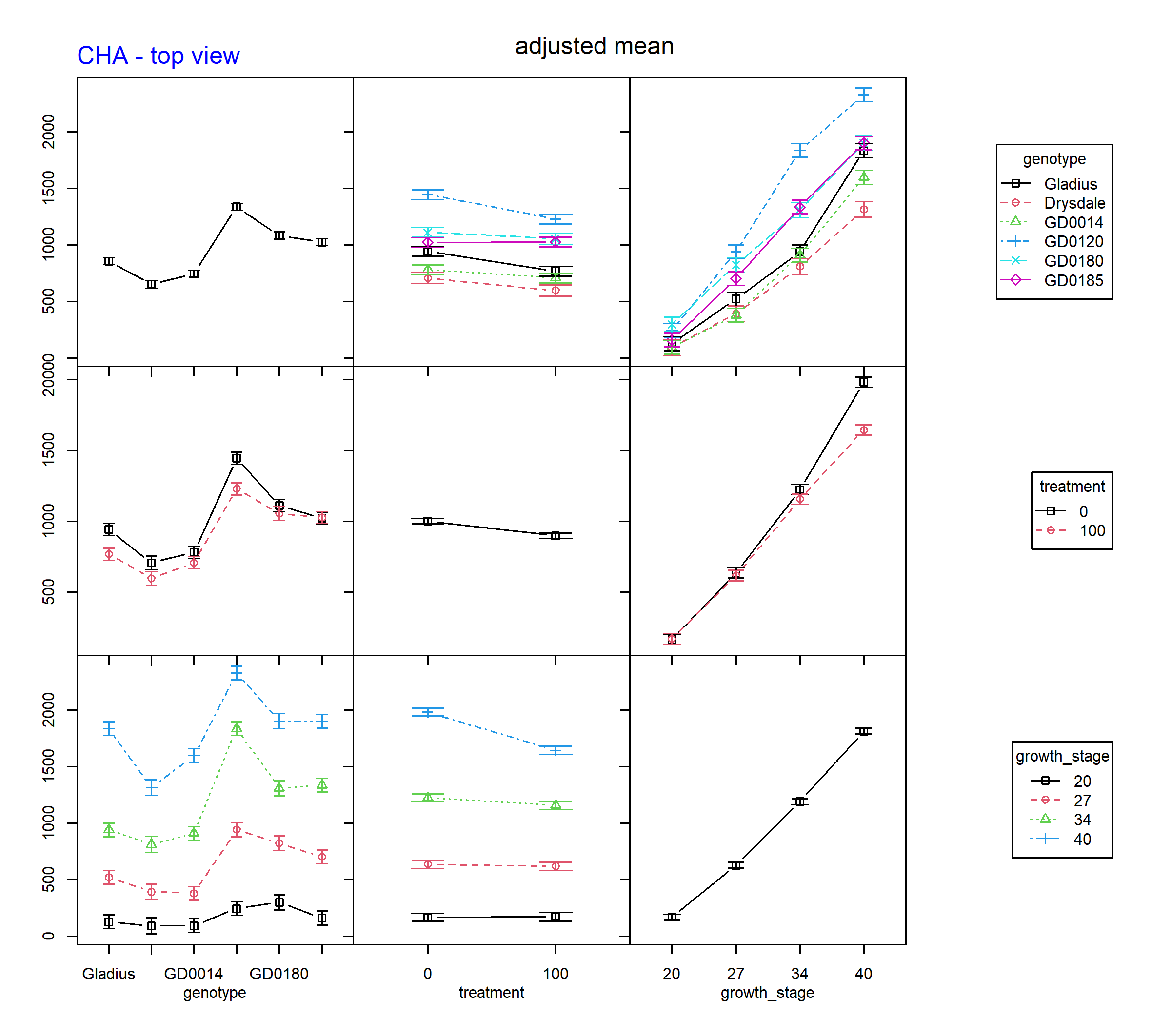


Figure S11. Analysis of genotype, salt treatment and growth stage effects on convex hull area – top view (CHA – top view) using Phia package.


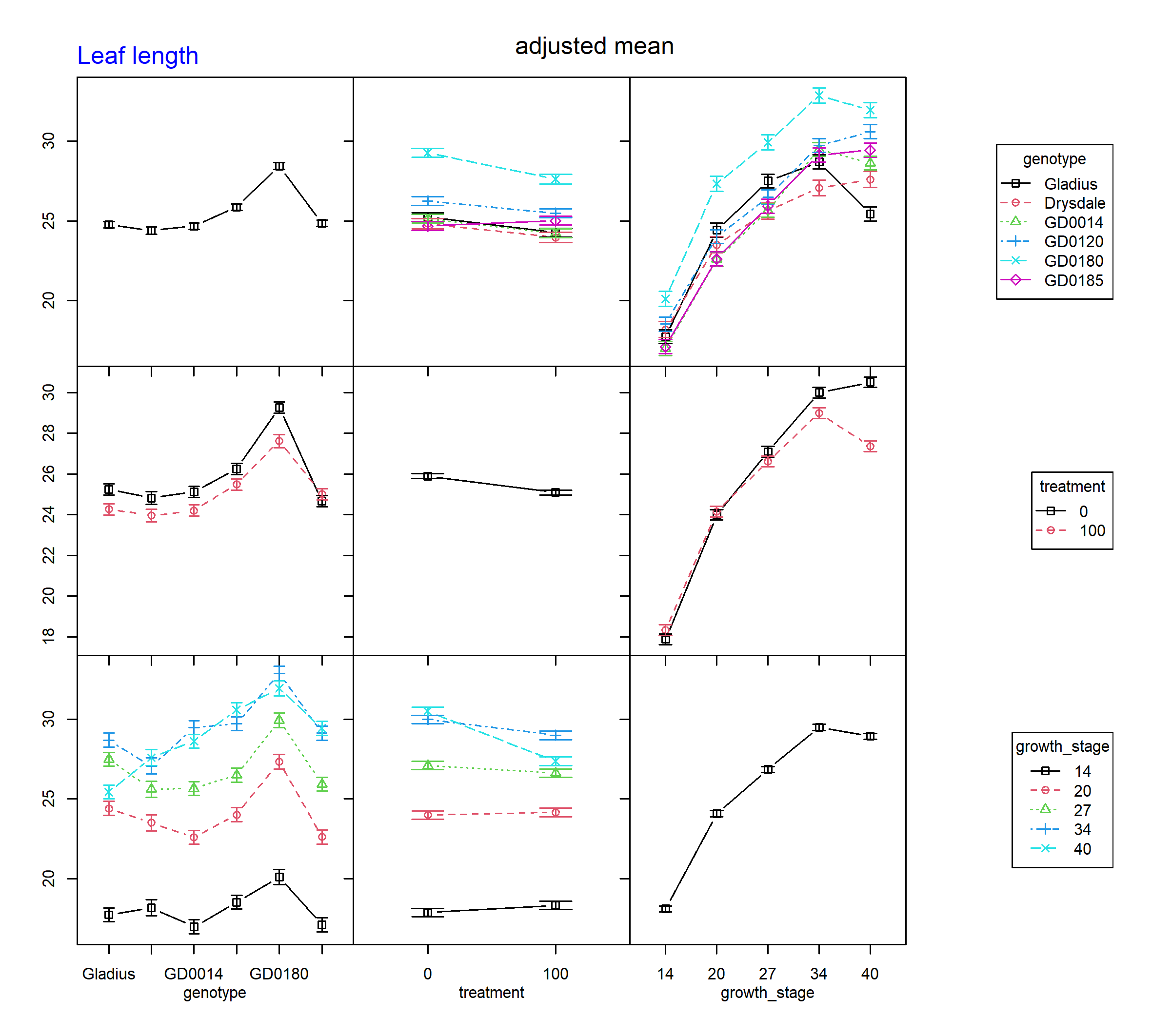


Figure S12. Analysis of genotype, salt treatment and growth stage effects on leaf length using Phia package.


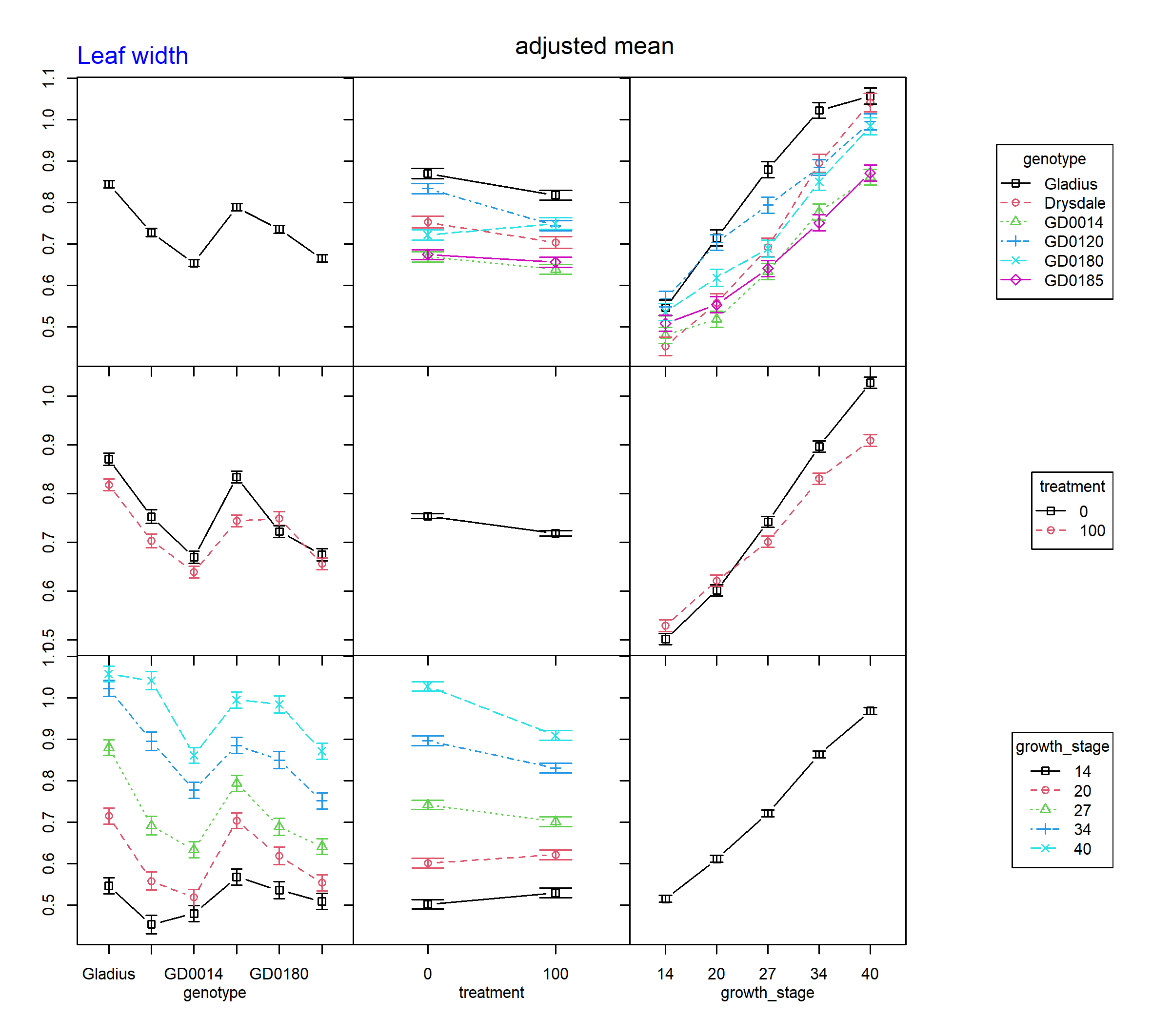


Figure S13. Analysis of genotype, salt treatment and growth stage effects on leaf width using Phia package.


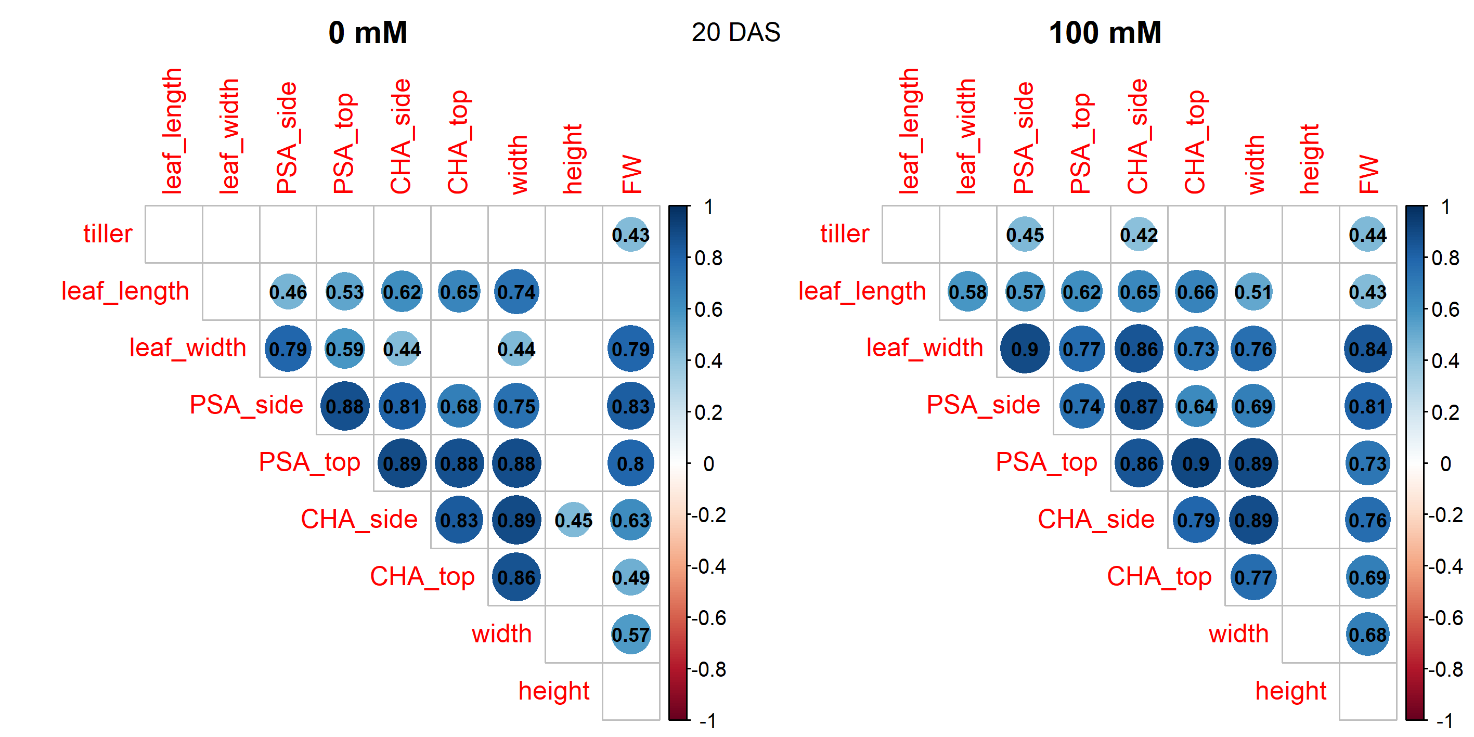


Figure S14. Correlation matrix between tiller number, leaf length, leaf width, projected shoot area (PSA) – side view (PSA_side), PSA – top view (PSA_top), convex hull area (CHA) – side view (CHA_side), CHA – top view, plant width, plant height at 20 DAS and shoot fresh weight (FW) at 45 DAS under control (0 mM NaCl) and under salt treatment (100 mM NaCl). Colour is significant (P < 0.05); blank is not significant (P < 0.05); DAS: days after sowing.


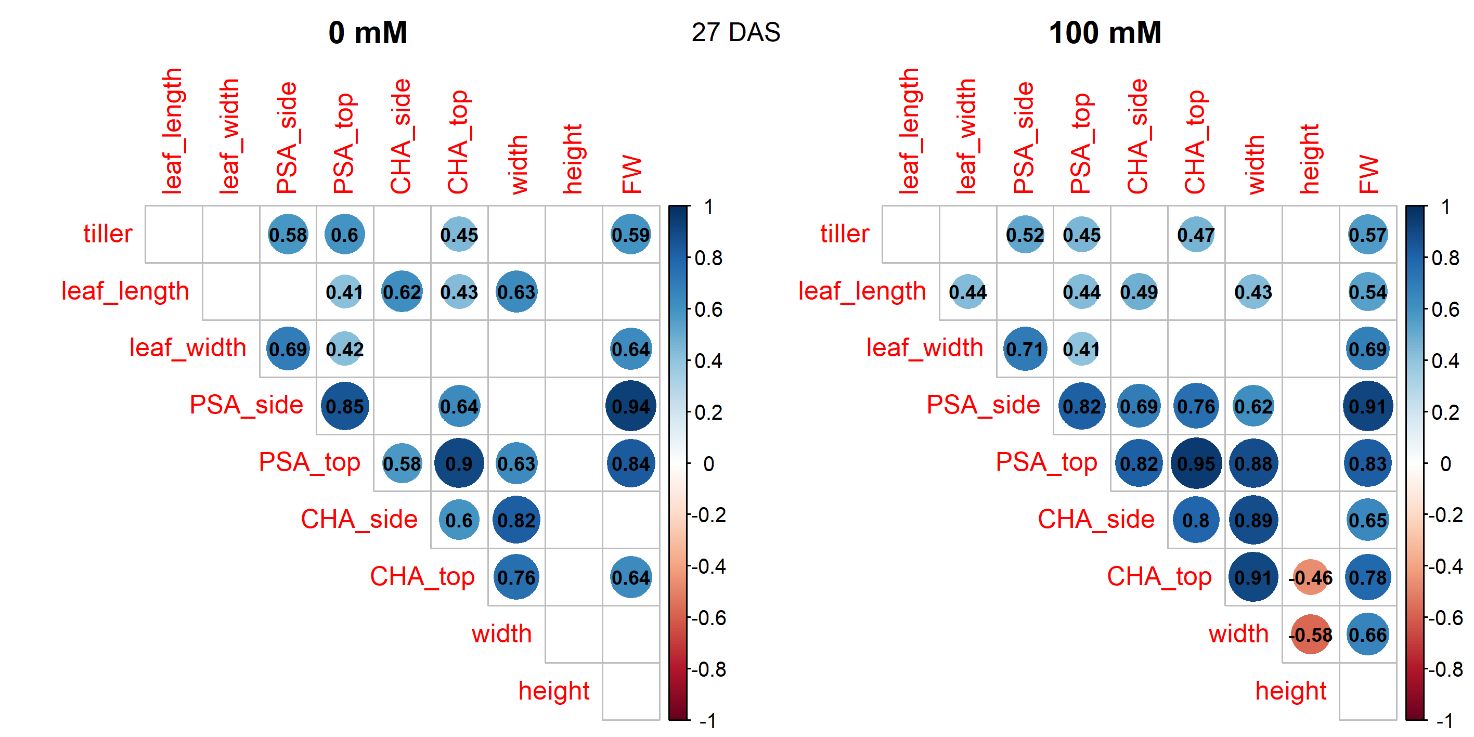


Figure S15. Correlation matrix between tiller number, leaf length, leaf width, projected shoot area (PSA) – side view (PSA_side), PSA – top view (PSA_top), convex hull area (CHA) – side view (CHA_side), CHA – top view, plant width, plant height at 27 DAS and shoot fresh weight (FW) at 45 DAS under control (0 mM NaCl) and under salt treatment (100 mM NaCl). Color is significant (P < 0.05); blank is not significant (P < 0.05); DAS: days after sowing.


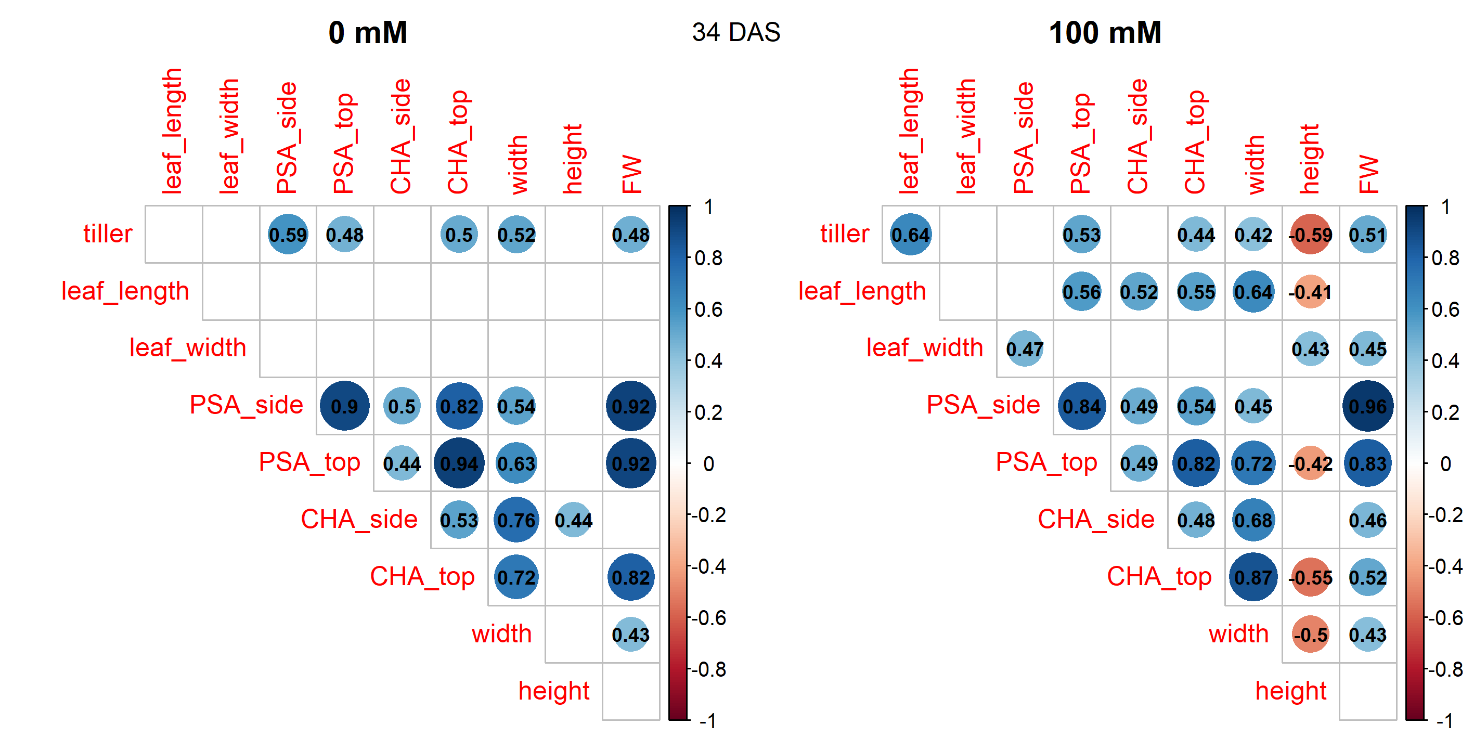


Figure S16. Correlation matrix between tiller number, leaf length, leaf width, projected shoot area (PSA) – side view (PSA_side), PSA – top view (PSA_top), convex hull area (CHA) – side view (CHA_side), CHA – top view, plant width, plant height at 34 DAS and shoot fresh weight (FW) at 45 DAS under control (0 mM NaCl) and under salt treatment (100 mM NaCl). Color is significant (P < 0.05); blank is not significant (P < 0.05); DAS: days after sowing.


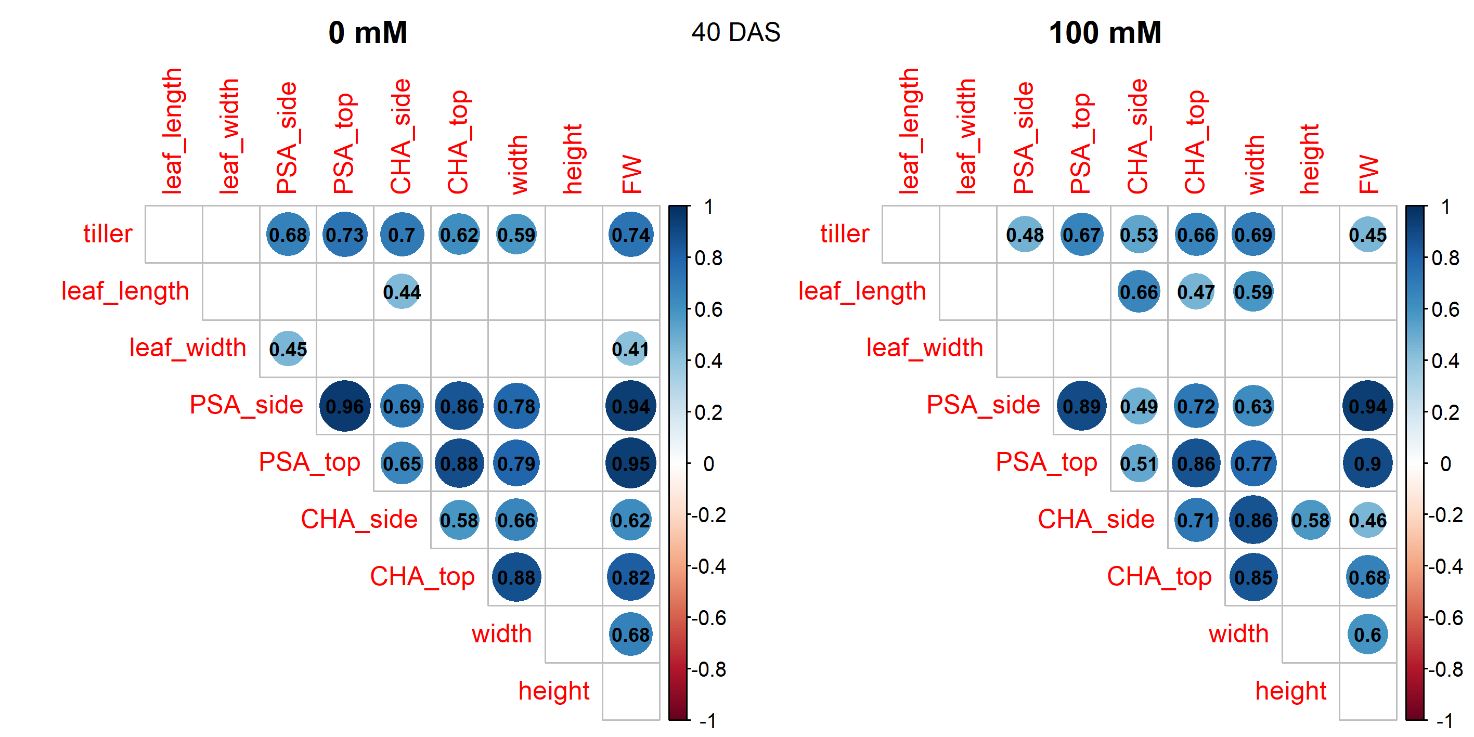


Figure S17. Correlation matrix between tiller number, leaf length, leaf width, projected shoot area (PSA) – side view (PSA_side), PSA – top view (PSA_top), convex hull area (CHA) – side view (CHA_side), CHA – top view, plant width, plant height at 40 DAS and shoot fresh weight (FW) at 45 DAS under control (0 mM NaCl) and under salt treatment (100 mM NaCl). Color is significant (P < 0.05); blank is not significant (P < 0.05); DAS: days after sowing.

***Photosynthesis parameters***


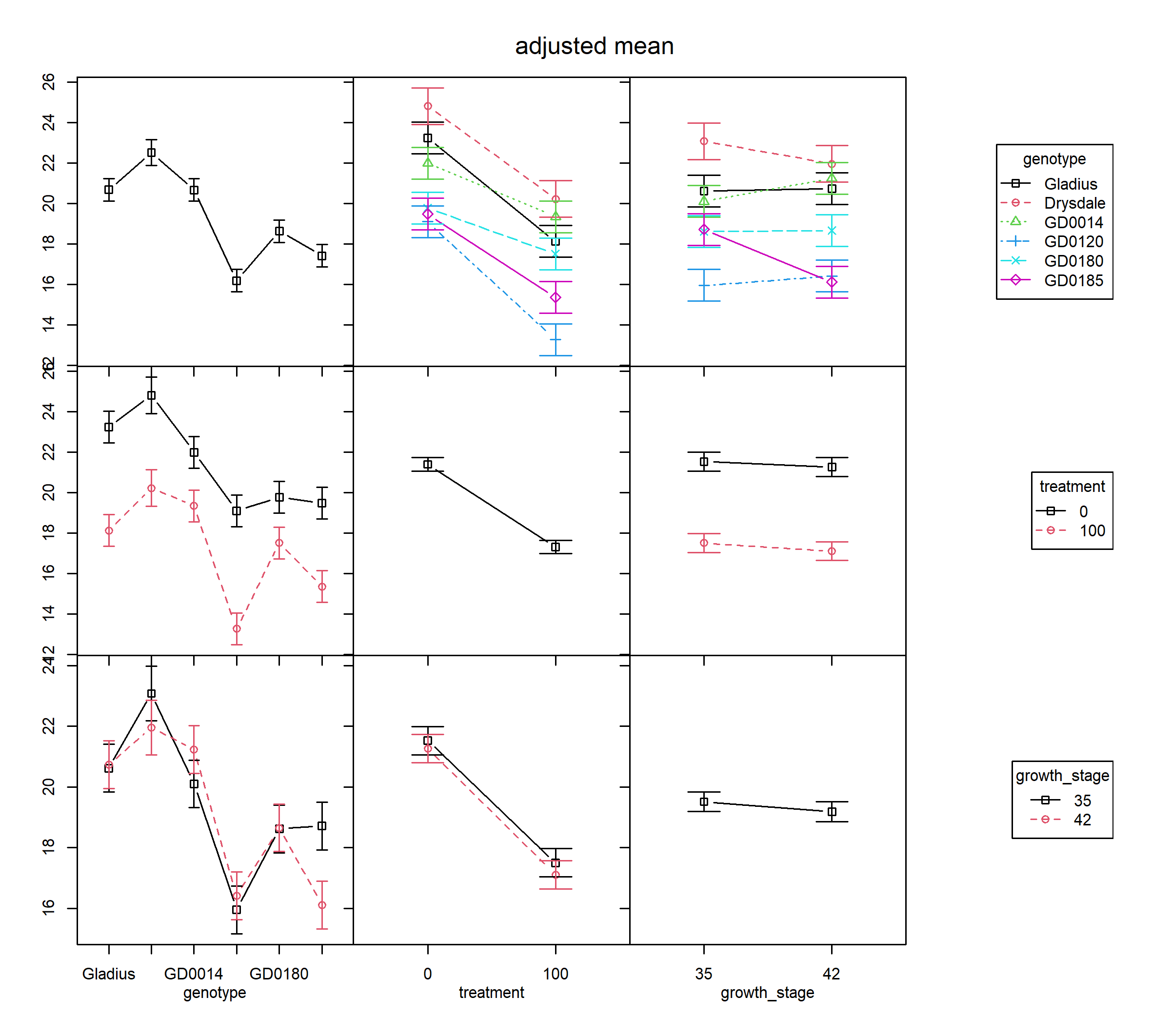


Figure S18. Analysis of genotype, salt treatment and growth stage effects on photosynthetic rate (Pn) using Phia package.


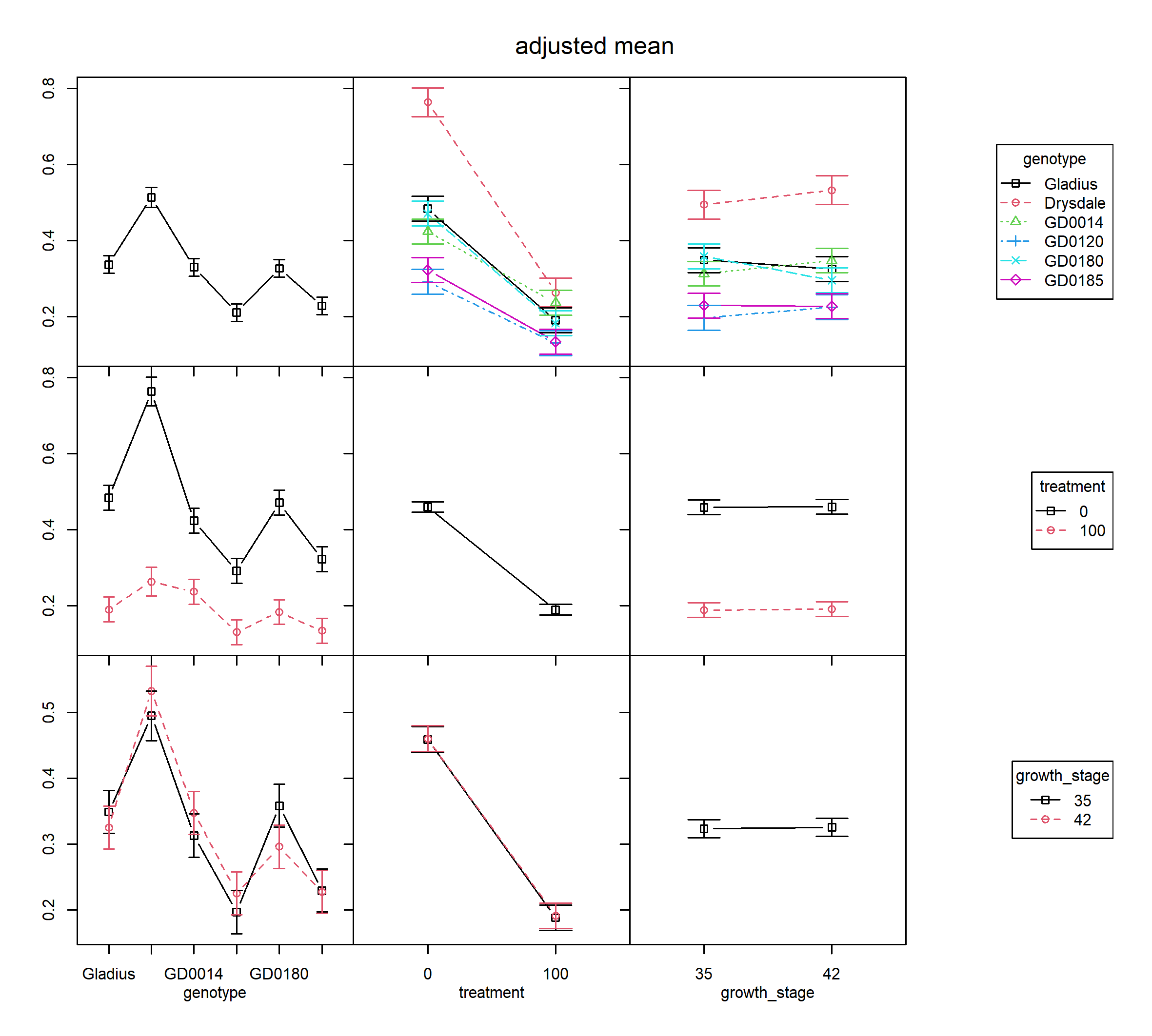


Figure 19. Analysis of genotype, salt treatment and growth stage effects on stomatal conductance (Cond) using Phia package.

**
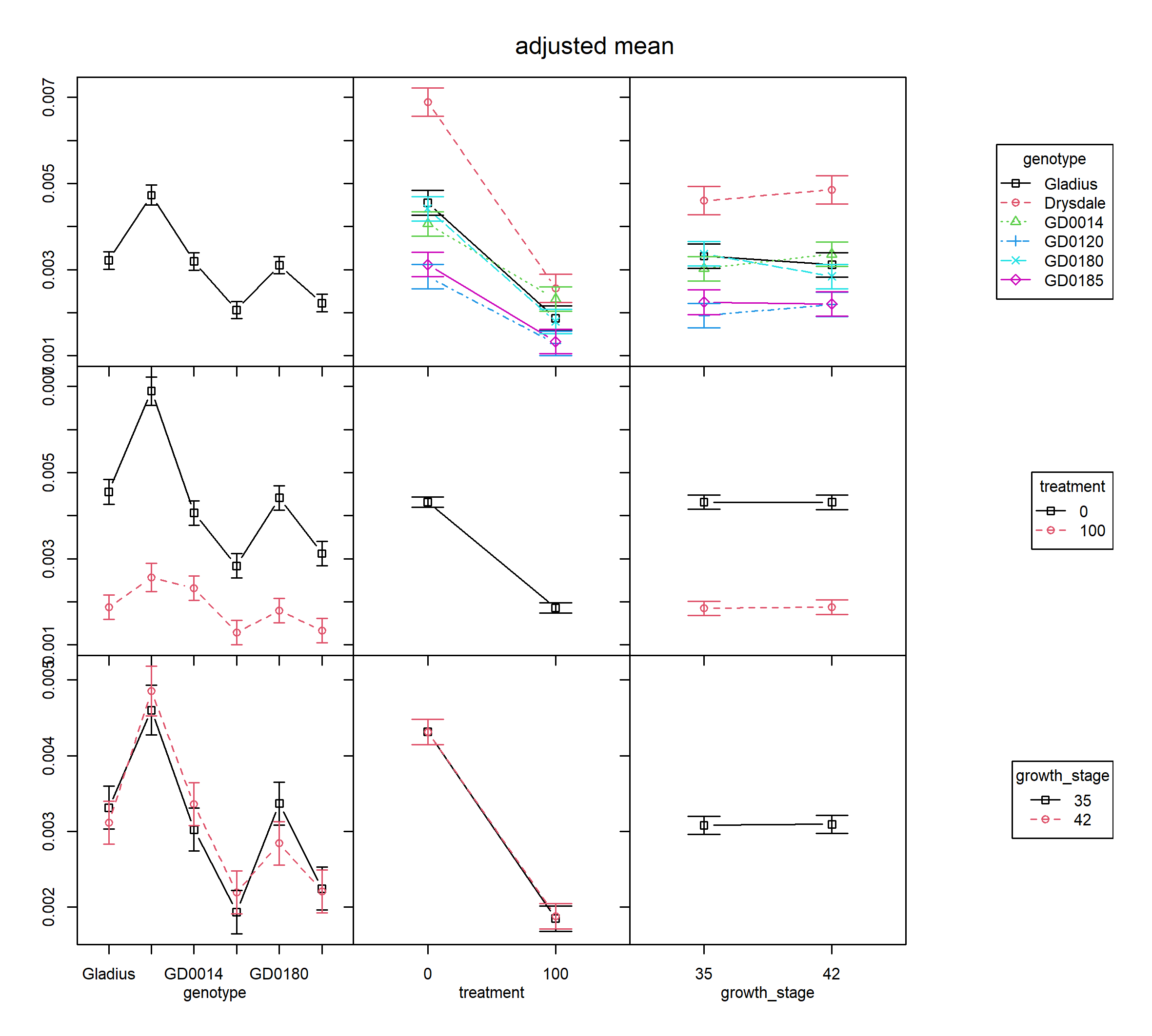
**

Figure 20. Analysis of genotype, salt treatment and growth stage effects on transpiration rate (E) using Phia package.


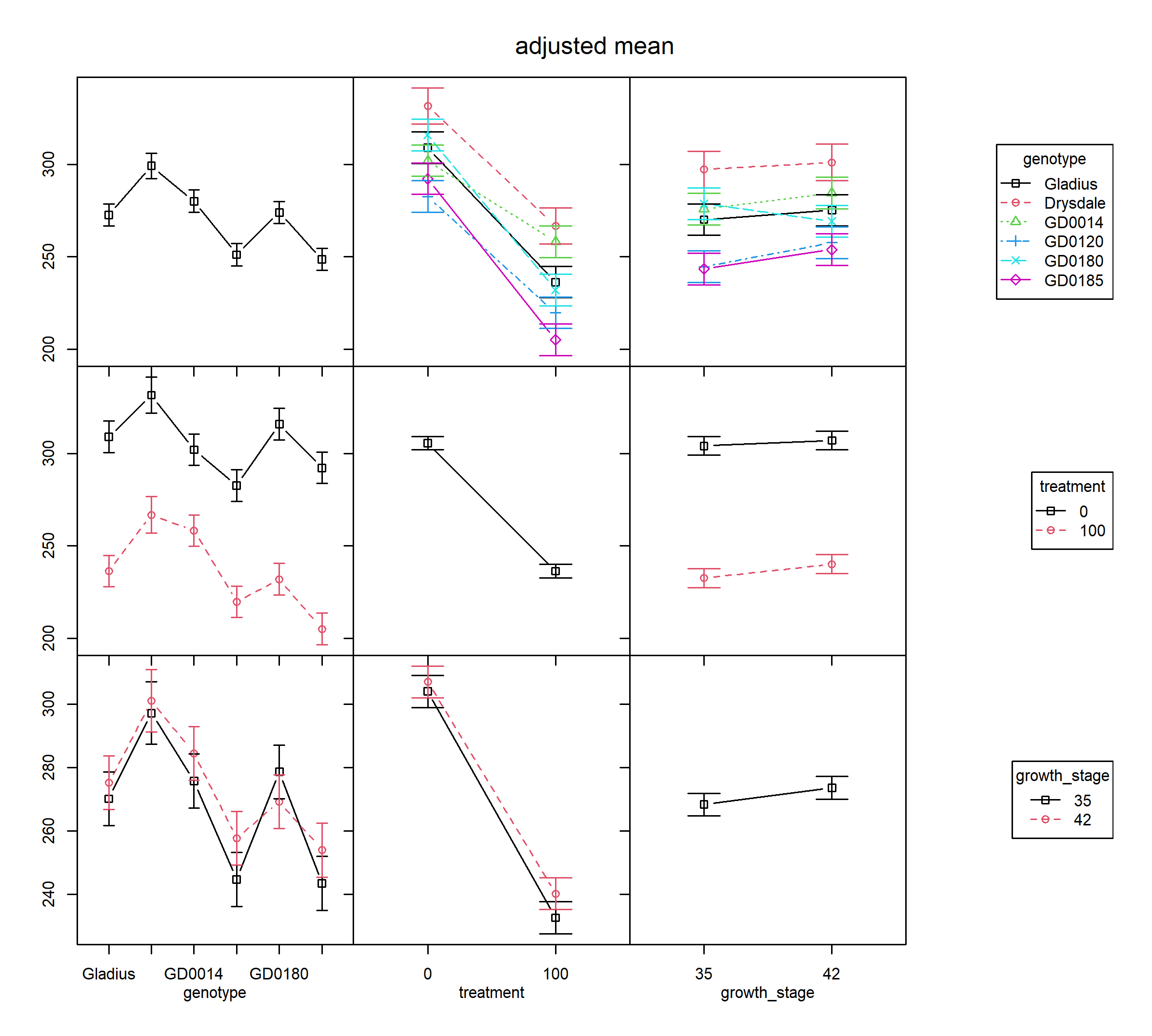


Figure S21. Analysis of genotype, salt treatment and growth stage effects on intercellular CO_2_ concentration (Ci) using Phia package.

.


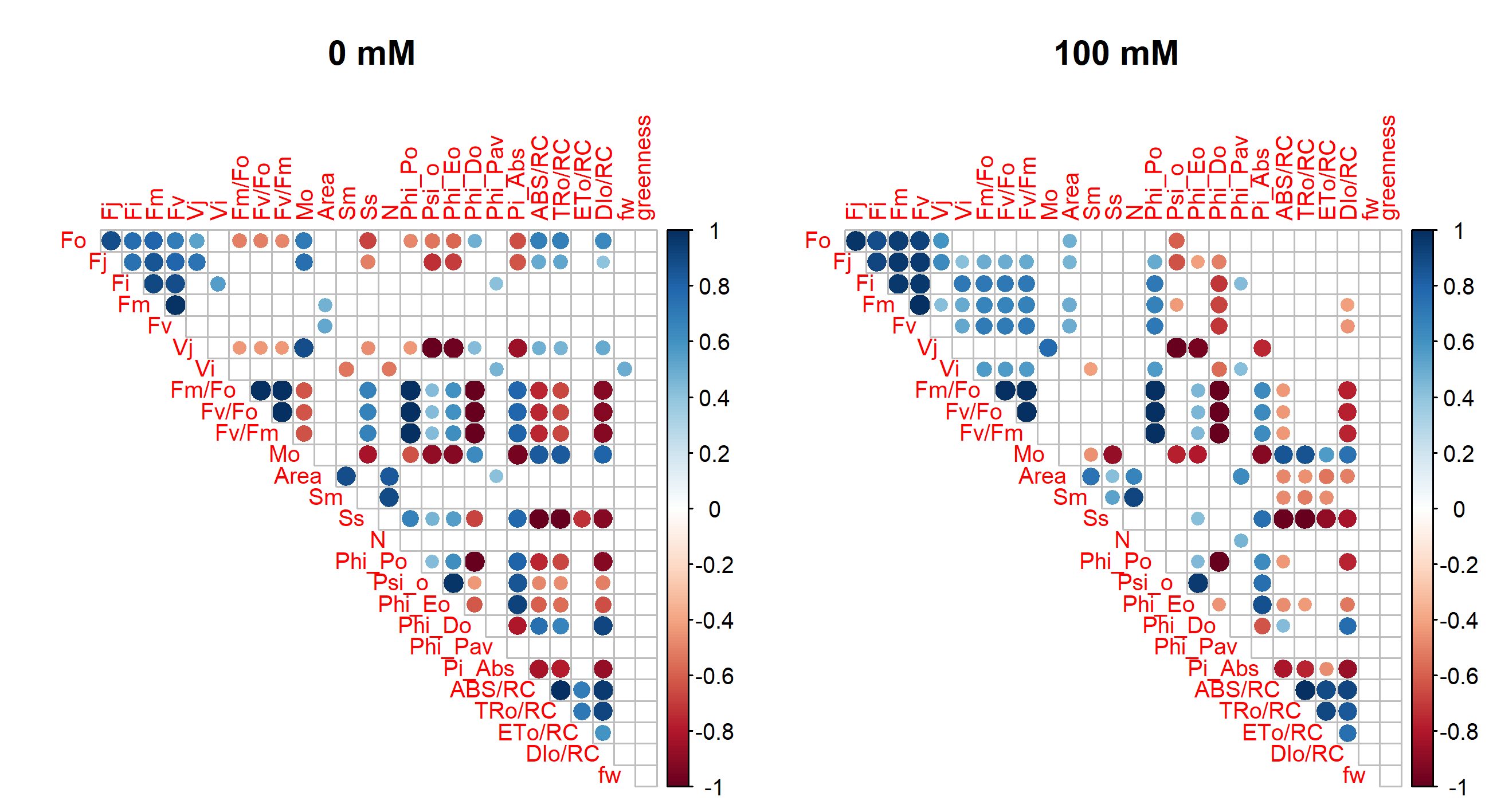


Figure S22. Correlation between OJIP parameters at 41 days after sowing (DAS) and fresh weight and greenness under control and treatment (100 mM NaCl) at 45 DAS. Color is significant (P < 0.05); blank is not significant (P < 0.05).

***Leaf mineral concentration***


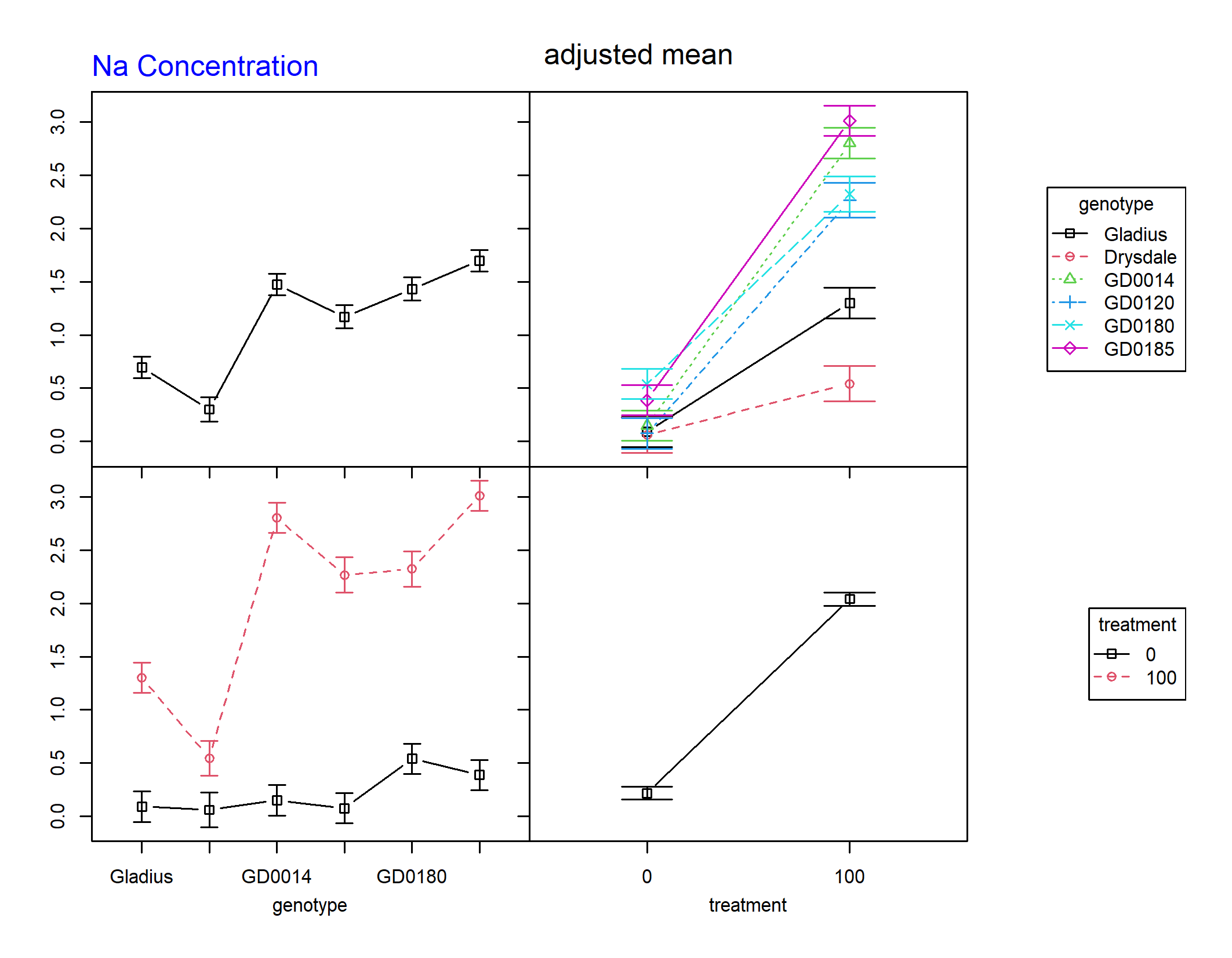


Figure S23. Analysis of genotype, salt treatment and growth stage effects on leaf Na concentration using Phia package.


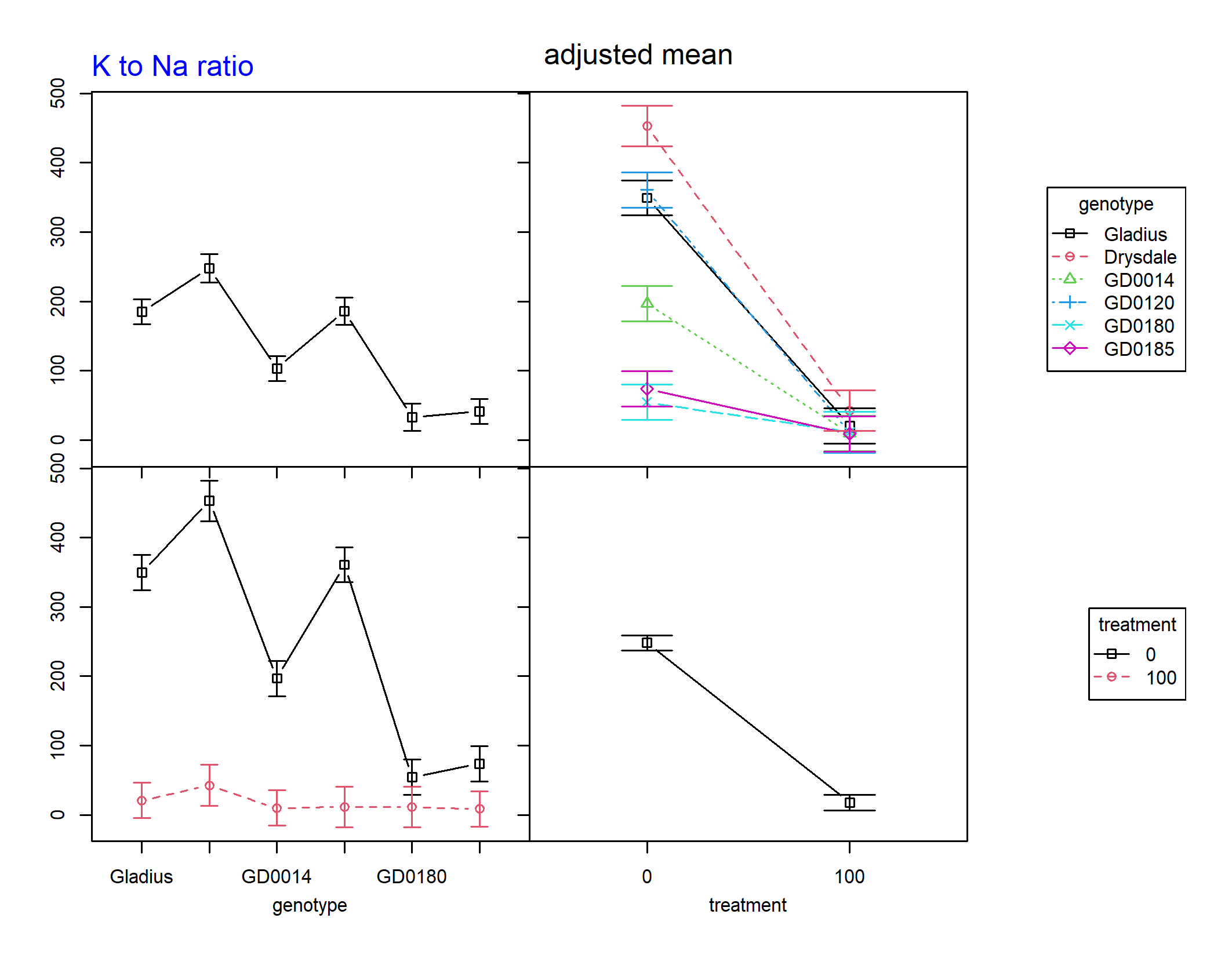


Figure S24. Analysis of genotype, salt treatment and growth stage effects on K to Na ratio using Phia package.

**Supplementary tables**

***Shoot parameters***

Table S1. Effect of genotype and treatment on fresh shoot weight, leaf water content at 45 days after sowing (DAS) and greenness at 36 DAS. The results were the mean ± standard deviation (n=4). Different letters show significantly between genotype within a column (P < 0.05); the same letter shows no significant difference (P < 0.05).

| **genotype** | **treatment** | **fresh shoot weight (g/plant)** | **water content (%)** | **greenness** |
| --- | --- | --- | --- | --- |
| ***Effect of treatment (main effect)*** | |  |  |  |
|  | 0 | 21.99 ± 3.52^a^ | 82.85 ± 1.96^a^ | 0.77 ± 0.01 |
|  | 100 | 13.11 ± 1.81^b^ | 80.07 ± 1.53^b^ | 0.77 ± 0.01 |
| ***Effect of genotype (main effect)*** | |  |  |  |
| Gladius |  | 18.55 ± 4.65 | 81.03 ± 1.76^a^ | 0.77 ± 0.01^ab^ |
| Drysdale |  | 15.15 ± 4.29 | 79.17 ± 2.54^b^ | 0.79 ± 0.01^a^ |
| GD0014 |  | 15.47 ± 4.44 | 81.9 ± 2.09^a^ | 0.76 ± 0.01^b^ |
| GD0120 |  | 21.71 ± 7.42 | 81.61 ± 2.01^ab^ | 0.77 ± 0.01^ab^ |
| GD0180 |  | 17.44 ± 3.71 | 82.68 ± 2.39^a^ | 0.77 ± 0.01^ab^ |
| GD0185 |  | 16.93 ± 4.8 | 82.13 ± 1.91^a^ | 0.76 ± 0.02^b^ |
| ***Genotype x salt treatment*** | |  |  |  |
| Gladius | 0 | 22.83 ± 1.11 | 82.45 ± 1.29 | 0.77 ± 0.01 |
| Drysdale | 0 | 19.01 ± 0.85 | 81.01 ± 2.29 | 0.79 ± 0.01 |
| GD0014 | 0 | 19.57 ± 0.98 | 83.57 ± 1.35 | 0.76 ± 0.01 |
| GD0120 | 0 | 28.55 ± 1.44 | 82.51 ± 2.54 | 0.77 ± 0 |
| GD0180 | 0 | 20.12 ± 2.19 | 84.45 ± 1.28 | 0.77 ± 0.01 |
| GD0185 | 0 | 21.15 ± 0.85 | 82.67 ± 2.3 | 0.77 ± 0.01 |
| Gladius | 100 | 14.27 ± 0.73 | 79.6 ± 0.42 | 0.77 ± 0.01 |
| Drysdale | 100 | 11.28 ± 0.72 | 77.33 ± 0.83 | 0.78 ± 0 |
| GD0014 | 100 | 11.37 ± 0.59 | 80.22 ± 0.96 | 0.76 ± 0.02 |
| GD0120 | 100 | 14.87 ± 1.28 | 80.72 ± 0.93 | 0.77 ± 0.01 |
| GD0180 | 100 | 13.86 ± 0.71 | 80.32 ± 0.2 | 0.77 ± 0 |
| GD0185 | 100 | 12.72 ± 2.39 | 81.58 ± 1.56 | 0.76 ± 0.02 |
| ***Anova analysis (P value)*** | |  |  |  |
| Genotype (G) |  | <0.001 | 0.004 | 0.008 |
| Treatment (T) |  | <0.001 | <0.001 | 0.316 |
| G x T |  | <0.001 | 0.375 | 0.923 |

Main effect contrasts were performed by using TukeyHSD test.

Table S2. Analysis of variance, interaction, simple effect (A), main effect contrasts and simple effect contrasts (B) for effects of genotype and salt treatment shoot on *fresh shoot weight*. 0 and 100: salt treatment at 0 and 100 mM NaCl, respectively.

A. Analysis of variance, interaction, simple effect

| **Source** | **df** | **SS** | **MS** | **F** | **Sig** |
| --- | --- | --- | --- | --- | --- |
| Genotype (G) | 5 | 218.41 | 43.68 | 25.07 | <0.001 |
| Treatment (T) | 1 | 892.46 | 892.46 | 512.24 | <0.001 |
| G x T | 5 | 61.28 | 12.26 | 7.03 | <0.001 |
| ***Simple effect (G at each T)*** |  |  |  |  |  |
| G at T = 0 | 5 | 241.79 | 48.36 | 27.76 | sig († ‡) |
| G at T = 100 | 5 | 42.19 | 8.44 | 4.84 | sig († ‡) |
| Error | 33 | 57.49 | 1.74 |  |  |

^†^F_(0.05/2, 5, 33)_ = 2.98 (Family error rate critical value)

^‡^F_(0.15/8, 5, 33)_ = 3.18 (Dun’s critical value)

B. Main effect contrasts and simple effect contrasts

| *Pairs* | *Treatment* | *Difference* | *conf.low* | *conf.high* | *sig* |
| --- | --- | --- | --- | --- | --- |
| ***Main effect contrasts*** |  |  |  |  |  |
| 0_vs_100 |  | 8.89 | -10.57 | -7.2 | **** |
| ***Simple effect contrasts (Pairwise between genotypes at each treatment)*** | | | |  |  |
| Gladius_vs_Drysdale | 0 | 3.82 | -6.98 | -0.65 | * |
| Gladius_vs_GD0014 | 0 | 3.26 | -6.23 | -0.29 | * |
| Gladius_vs_GD0120 | 0 | -5.72 | 2.02 | 9.43 | ** |
| Gladius_vs_GD0180 | 0 | 2.70 | -8.22 | 2.82 | ns |
| Gladius_vs_GD0185 | 0 | 1.68 | -4.53 | 1.17 | ns |
| Drysdale_vs_GD0014 | 0 | -0.56 | -2.44 | 3.56 | ns |
| Drysdale_vs_GD0120 | 0 | -9.54 | 5.78 | 13.30 | *** |
| Drysdale_vs_GD0180 | 0 | -1.11 | -4.52 | 6.75 | ns |
| Drysdale_vs_GD0185 | 0 | -2.14 | -0.77 | 5.04 | ns |
| GD0014_vs_GD0120 | 0 | -8.98 | 5.35 | 12.61 | *** |
| GD0014_vs_GD0180 | 0 | -0.55 | -5.02 | 6.13 | ns |
| GD0014_vs_GD0185 | 0 | -1.58 | -1.02 | 4.17 | ns |
| GD0120_vs_GD0180 | 0 | 8.43 | -13.94 | -2.92 | ** |
| GD0120_vs_GD0185 | 0 | 7.40 | -11.01 | -3.79 | ** |
| GD0180_vs_GD0185 | 0 | -1.02 | -4.64 | 6.69 | ns |
| Gladius_vs_Drysdale | 100 | 2.99 | -5.47 | -0.51 | * |
| Gladius_vs_GD0014 | 100 | 2.90 | -4.79 | -1.01 | ** |
| Gladius_vs_GD0120 | 100 | -0.60 | -2.60 | 3.80 | ns |
| Gladius_vs_GD0180 | 100 | 0.42 | -2.87 | 2.03 | ns |
| Gladius_vs_GD0185 | 100 | 1.56 | -7.90 | 4.78 | ns |
| Drysdale_vs_GD0014 | 100 | -0.09 | -2.38 | 2.56 | ns |
| Drysdale_vs_GD0120 | 100 | -3.59 | 0.27 | 6.90 | * |
| Drysdale_vs_GD0180 | 100 | -2.57 | -0.21 | 5.35 | ns |
| Drysdale_vs_GD0185 | 100 | -1.43 | -4.84 | 7.70 | ns |
| GD0014_vs_GD0120 | 100 | -3.50 | 0.26 | 6.74 | * |
| GD0014_vs_GD0180 | 100 | -2.48 | 0.05 | 4.92 | * |
| GD0014_vs_GD0185 | 100 | -1.34 | -5.11 | 7.80 | ns |
| GD0120_vs_GD0180 | 100 | 1.01 | -4.32 | 2.29 | ns |
| GD0120_vs_GD0185 | 100 | 2.16 | -8.16 | 3.84 | ns |
| GD0180_vs_GD0185 | 100 | 1.14 | -7.42 | 5.14 | ns |

Simple contrasts were performed by using Games Howell test.

*, ** and ***: significant at P < 0.05, P < 0.01 and P < 0.001, respectively.

ns: not significant.

Table S3. Three-way ANOVA analysis on the effect of genotype, salt treatment and growth stage on tiller number, leaf length, leaf width, plant height, plant width, projected shoot area (PSA) - side view, PSA - top view, convex hull area (CHA) – side view, CHA – top view. Two salt levels (0 and 100 mM NaCl) were applied for plants at 14 days after sowing (DAS). Leaf length and leaf width were measured on the youngest fully expanded leaf.

| Source |  | Tiller number |  | Leaf length |  | Leaf width |  | Height |  | Width |
| --- | --- | --- | --- | --- | --- | --- | --- | --- | --- | --- |
|  | df | *F* ratio | df | *F* ratio | df | *F* ratio | df | *F* ratio | df | *F* ratio |
| Genotype (G) | 5 | 60.85^***^ | 5 | 57.53^***^ | 5 | 70.97^***^ | 5 | 6.62*** | 5 | 77.68^***^ |
| Treatment (T) | 1 | 39.63^***^ | 1 | 22.45^***^ | 1 | 25.33^***^ | 1 | 9.89^**^ | 1 | 2.78 ns |
| Growth stage (GS) | 4 | 910.68^***^ | 4 | 651.54^***^ | 4 | 502.6^***^ | 4 | 152.56^***^ | 4 | 832.16^***^ |
| G x T | 5 | 2.88^*^ | 5 | 2.56^*^ | 5 | 4.79^***^ | 5 | 0.97 ns | 5 | 1.35 |
| G x GS | 20 | 14.07^***^ | 20 | 5.51^***^ | 20 | 4.97^***^ | 20 | 2.77^***^ | 20 | 9.72^***^ |
| T x GS | 4 | 22.05^***^ | 4 | 13.96^***^ | 4 | 14.44^***^ | 4 | 1.79 ns | 4 | 7.61^***^ |
| G x T x GS | 20 | 0.89 ns | 20 | 1.75^*^ | 20 | 0.58 ns | 20 | 0.94 ns | 20 | 1.5 ns |
| Error | 165 |  | 165 |  | 165 |  | 165 |  | 165 |  |

F Ratio are from three-way ANOVA analysis. *, ** and *** significant at P < 0.05, P < 0.01 and P < 0.001, respectively; ns: not significant.

Table S3 (continuing). Three-way ANOVA analysis on the effect of genotype, salt treatment and growth stage on tiller number, leaf length, leaf width, plant height, plant width, projected shoot area (PSA) - side view, PSA - top view, convex hull area (CHA) – side view, CHA – top view. Two salt levels (0 and 100 mM NaCl) were applied for plants at 14 days after sowing (DAS). Leaf length and leaf width were measured on the youngest fully expanded leaf.

| **Source** |  | PSA - side view |  | PSA - top view |  | CHA - side view |  | CHA - top view |
| --- | --- | --- | --- | --- | --- | --- | --- | --- |
|  | df | *F* ratio | df | *F* ratio | df | *F* ratio | df | *F* ratio |
| Genotype (G) | 5 | 64.24^***^ | 5 | 76.76^***^ | 5 | 45.72^***^ | 5 | 62.59^***^ |
| Treatment (T) | 1 | 257.57^***^ | 1 | 159.35^***^ | 1 | 36.79^***^ | 1 | 16.84^***^ |
| Growth stage (GS) | 4 | 2001.36^***^ | 3 | 1800.4^***^ | 4 | 847.47^***^ | 3 | 791.57^***^ |
| G x T | 5 | 7.83^***^ | 5 | 8.69^***^ | 5 | 1.46 ns | 5 | 1.77 ns |
| G x GS | 20 | 12.81^***^ | 15 | 12.44^***^ | 20 | 8.55^***^ | 15 | 6.15^***^ |
| T x GS | 4 | 104.99^***^ | 3 | 76.32^***^ | 4 | 12.91^***^ | 3 | 9.86^***^ |
| G x T x GS | 20 | 2.08^**^ | 15 | 1.82^*^ | 20 | 1.33 ns | 15 | 0.84 ns |
| Error | 165 |  | 132 |  | 165 |  | 132 |  |

F Ratio are from three-way ANOVA analysis. * and *** significant at P < 0.05 and P < 0.001, respectively; ns: not significant.

Table S4. Ratio of shoot parameters between salt treatment (100 mM) and without treatment of six wheat genotype at different growth stages.

| Growth stage (DAS) | genotype | tiller | Leaf length | Leaf width | Width | Height | PSA – side view | CHA – side view | PSA – top view | CHA – top view |
| --- | --- | --- | --- | --- | --- | --- | --- | --- | --- | --- |
| Growth stage | | | | | | | | | | |
| 14 |  | 1.1 | 1.02 | 1.05 | 1.04 | 1.01 | 1.06 | 1.02 | NA | NA |
| 20 |  | 1.0 | 1.00 | 1.03 | 1.02 | 0.98 | 0.99 | 1.00 | 0.99 | 1.00 |
| 27 |  | 1.0 | 0.98 | 0.95 | 1.03 | 0.93 | 0.89 | 0.92 | 0.93 | 0.96 |
| 34 |  | 0.9 | 0.97 | 0.93 | 0.99 | 0.9 | 0.77 | 0.80 | 0.81 | 0.94 |
| 40 |  | 0.8 | 0.90 | 0.88 | 0.87 | 0.92 | 0.66 | 0.80 | 0.70 | 0.83 |
| Growth stage x genotype | | | | | | | | | | |
| 14 | Gladius | 1.40 | 1.05 | 1.04 | 1.35 | 0.98 | 1.11 | 1.36 | NA | NA |
| 14 | Drysdale | 0.80 | 1.06 | 1.06 | 1.32 | 1.15 | 1.15 | 1.53 | NA | NA |
| 14 | GD0014 | 1.00 | 1.01 | 1.08 | 0.99 | 0.99 | 1.04 | 0.96 | NA | NA |
| 14 | GD0120 | 1.00 | 0.99 | 0.97 | 1.03 | 1.02 | 1.00 | 1.04 | NA | NA |
| 14 | GD0180 | 1.30 | 1.00 | 1.13 | 0.90 | 1.00 | 1.14 | 0.93 | NA | NA |
| 14 | GD0185 | 1.00 | 1.05 | 1.06 | 0.91 | 1.01 | 0.98 | 0.77 | NA | NA |
| 20 | Gladius | 1.00 | 1.04 | 0.99 | 1.22 | 0.94 | 1.03 | 1.17 | 1.13 | 1.56 |
| 20 | Drysdale | 1.20 | 1.05 | 0.97 | 0.97 | 1.06 | 1.05 | 1.14 | 1.01 | 0.78 |
| 20 | GD0014 | 0.80 | 0.98 | 1.04 | 1.01 | 1.00 | 1.02 | 0.99 | 0.97 | 1.11 |
| 20 | GD0120 | 1.00 | 0.97 | 0.94 | 0.88 | 1.03 | 0.92 | 0.88 | 0.74 | 0.79 |
| 20 | GD0180 | 1.20 | 0.98 | 1.22 | 1.02 | 0.96 | 1.08 | 1.08 | 1.18 | 1.10 |
| 20 | GD0185 | 0.90 | 1.01 | 1.07 | 1.16 | 0.91 | 0.92 | 0.92 | 1.14 | 1.11 |
| 27 | Gladius | 1.10 | 0.99 | 0.99 | 1.06 | 0.93 | 0.90 | 1.01 | 0.98 | 1.15 |
| 27 | Drysdale | 1.00 | 1.05 | 0.88 | 0.88 | 0.98 | 0.91 | 0.97 | 0.95 | 0.92 |
| 27 | GD0014 | 1.00 | 0.97 | 0.94 | 1.09 | 0.90 | 0.88 | 0.94 | 0.94 | 1.00 |
| 27 | GD0120 | 0.90 | 0.97 | 0.87 | 1.10 | 0.92 | 0.78 | 0.95 | 0.81 | 0.89 |
| 27 | GD0180 | 1.00 | 0.94 | 1.04 | 1.03 | 0.94 | 1.03 | 0.90 | 1.09 | 1.01 |
| 27 | GD0185 | 1.00 | 0.98 | 0.97 | 1.08 | 0.88 | 0.92 | 0.88 | 0.95 | 0.93 |
| 34 | Gladius | 1.00 | 0.92 | 0.92 | 0.92 | 0.96 | 0.78 | 0.82 | 0.87 | 0.84 |
| 34 | Drysdale | 0.60 | 0.87 | 0.88 | 1.01 | 1.19 | 0.84 | 1.07 | 0.75 | 0.92 |
| 34 | GD0014 | 0.80 | 0.98 | 0.94 | 0.93 | 0.87 | 0.73 | 0.76 | 0.87 | 0.96 |
| 34 | GD0120 | 0.90 | 1.01 | 0.87 | 1.03 | 0.84 | 0.71 | 0.81 | 0.69 | 0.89 |
| 34 | GD0180 | 0.90 | 0.97 | 1.00 | 0.99 | 0.88 | 0.84 | 0.86 | 0.90 | 0.95 |
| 34 | GD0185 | 0.90 | 1.05 | 0.96 | 1.06 | 0.74 | 0.76 | 0.68 | 0.88 | 1.11 |
| 40 | Gladius | 0.80 | 0.84 | 0.84 | 0.71 | 0.96 | 0.66 | 0.65 | 0.68 | 0.69 |
| 40 | Drysdale | 0.60 | 0.86 | 0.95 | 1.04 | 0.88 | 0.69 | 0.91 | 0.74 | 0.78 |
| 40 | GD0014 | 0.80 | 0.90 | 0.87 | 0.77 | 0.80 | 0.64 | 0.66 | 0.71 | 0.85 |
| 40 | GD0120 | 0.80 | 0.92 | 0.85 | 0.93 | 0.92 | 0.59 | 0.82 | 0.62 | 0.81 |
| 40 | GD0180 | 0.90 | 0.86 | 0.92 | 0.90 | 0.97 | 0.76 | 0.94 | 0.76 | 0.90 |
| 40 | GD0185 | 0.80 | 0.99 | 0.88 | 0.98 | 1.05 | 0.69 | 0.91 | 0.74 | 0.95 |

DAS: days after sowing; PSA: projected shoot area; CHA: convex hull area.

***Photosynthesis parameters***

Table S5. Analysis of variance, simple interaction, simple effect, main effect contrasts, simple effect contrasts and simple simple effect contrasts for effects of genotype, salt treatment and growth stage on *photosynthetic rate* (Pn). 0 and 100: salt treatment at 0 and 100 mM NaCl, respectively; DAS: day after sowing.

| **Source** |  | **df** | **SS** | **MS** | **F** | **Sig** |
| --- | --- | --- | --- | --- | --- | --- |
| Genotype (G) |  | 5 | 402.92 | 80.58 | 16.44 | <0.001 |
| Treatment (T) |  | 1 | 380.67 | 380.67 | 77.65 | <0.001 |
| Growth stage (GS) |  | 1 | 2.01 | 2.01 | 0.41 | 0.525 |
| G x T |  | 5 | 38.77 | 7.75 | 1.58 | 0.177 |
| G x GS |  | 5 | 34.82 | 6.96 | 1.42 | 0.228 |
| T x GS |  | 1 | 0.26 | 0.26 | 0.05 | 0.819 |
| G x T x GS |  | 5 | 81.19 | 16.24 | 3.31 | 0.010 |
| ***G x T at each GS*** | | | | | | |
| G x T at 35 DAS |  | 5 | 56.71 | 11.34 | 2.31 | ns |
| G x T at 42 DAS |  | 5 | 63.25 | 12.65 | 2.58 | sig (‡) |
| ***G at T (GS = 42 DAS)*** | | | | | | |
| G at T = 0 and GS = 42 |  | 5 | 139.1 | 27.81 | 5.67 | sig († ‡) |
| G at T =100 and GS = 42 | | 5 | 161.38 | 32.28 | 6.58 | sig († ‡) |
| ***Main effect contrast between treatments*** | | | | | | |
| 0 vs 100 |  | 1 | 380.31 | 380.31 | 77.57 | <0.001 |
| ***Simple effect contrasts (Pairwise between genotypes at 35 DAS)*** | | | | | | |
| *Pairs* |  |  |  |  |  |  |
| Gladius_vs_Drysdale |  | 1 | 20.68 | 20.68 | 4.22 | ns |
| Gladius_vs_GD0014 |  | 1 | 1.07 | 1.07 | 0.22 | ns |
| Gladius_vs_GD0120 |  | 1 | 87.13 | 87.13 | 17.77 | sig (♣) |
| Gladius_vs_GD0180 |  | 1 | 16.03 | 16.03 | 3.27 | ns |
| Gladius_vs_GD0185 |  | 1 | 14.51 | 14.51 | 2.96 | ns |
| Drysdale_vs_GD0014 |  | 1 | 30.32 | 30.32 | 6.18 | ns |
| Drysdale_vs_GD0120 |  | 1 | 173.94 | 173.94 | 35.48 | sig (♣) |
| Drysdale_vs_GD0180 |  | 1 | 68.13 | 68.13 | 13.90 | sig (♣) |
| Drysdale_vs_GD0185 |  | 1 | 65.19 | 65.19 | 13.30 | sig (♣) |
| GD0014_vs_GD0120 |  | 1 | 68.86 | 68.86 | 14.04 | sig (♣) |
| GD0014_vs_GD0180 |  | 1 | 8.81 | 8.81 | 1.80 | ns |
| GD0014_vs_GD0185 |  | 1 | 7.69 | 7.69 | 1.57 | ns |
| GD0120_vs_GD0180 |  | 1 | 28.41 | 28.41 | 5.79 | ns |
| GD0120_vs_GD0185 |  | 1 | 30.52 | 30.52 | 6.23 | ns |
| GD0180_vs_GD0185 |  | 1 | 0.04 | 0.04 | 0.01 | ns |
| ***Simple simple effect contrasts (Pairwise between genotypes at each treatment and at 42 DAS)*** | | | | | | |
| *Pairs* | *Treatment* |  |  |  |  |  |
| Gladius_vs_Drysdale | 0 | 1 | 0.65 | 0.65 | 0.13 | ns |
| Gladius_vs_GD0014 | 0 | 1 | 0.91 | 0.91 | 0.18 | ns |
| Gladius_vs_GD0120 | 0 | 1 | 33.57 | 33.57 | 6.85 | ns |
| Gladius_vs_GD0180 | 0 | 1 | 50.32 | 50.32 | 10.26 | sig (♣) |
| Gladius_vs_GD0185 | 0 | 1 | 36.33 | 36.33 | 7.41 | ns |
| Drysdale_vs_GD0014 | 0 | 1 | 0.01 | 0.01 | 0.00 | ns |
| Drysdale_vs_GD0120 | 0 | 1 | 38.07 | 38.07 | 7.76 | ns |
| Drysdale_vs_GD0180 | 0 | 1 | 54.37 | 54.37 | 11.09 | ns |
| Drysdale_vs_GD0185 | 0 | 1 | 40.71 | 40.71 | 8.30 | ns |
| GD0014_vs_GD0120 | 0 | 1 | 45.49 | 45.49 | 9.28 | sig (♣) |
| GD0014_vs_GD0180 | 0 | 1 | 64.71 | 64.71 | 13.20 | sig (♣) |
| GD0014_vs_GD0185 | 0 | 1 | 48.62 | 48.62 | 9.92 | sig (♣) |
| GD0120_vs_GD0180 | 0 | 1 | 1.69 | 1.69 | 0.34 | ns |
| GD0120_vs_GD0185 | 0 | 1 | 0.05 | 0.05 | 0.01 | ns |
| GD0180_vs_GD0185 | 0 | 1 | 1.15 | 1.15 | 0.23 | ns |
| Gladius_vs_Drysdale | 100 | 1 | 5.75 | 5.75 | 1.17 | ns |
| Gladius_vs_GD0014 | 100 | 1 | 0.22 | 0.22 | 0.05 | ns |
| Gladius_vs_GD0120 | 100 | 1 | 41.25 | 41.25 | 8.41 | ns |
| Gladius_vs_GD0180 | 100 | 1 | 1.49 | 1.49 | 0.30 | ns |
| Gladius_vs_GD0185 | 100 | 1 | 49.48 | 49.48 | 10.09 | sig (♣) |
| Drysdale_vs_GD0014 | 100 | 1 | 3.85 | 3.85 | 0.79 | ns |
| Drysdale_vs_GD0120 | 100 | 1 | 69.62 | 69.62 | 14.20 | sig (♣) |
| Drysdale_vs_GD0180 | 100 | 1 | 0.97 | 0.97 | 0.20 | sig (♣) |
| Drysdale_vs_GD0185 | 100 | 1 | 79.40 | 79.40 | 16.19 | sig (♣) |
| GD0014_vs_GD0120 | 100 | 1 | 47.52 | 47.52 | 9.69 | sig (♣) |
| GD0014_vs_GD0180 | 100 | 1 | 0.56 | 0.56 | 0.11 | ns |
| GD0014_vs_GD0185 | 100 | 1 | 56.33 | 56.33 | 11.49 | sig (♣) |
| GD0120_vs_GD0180 | 100 | 1 | 58.41 | 58.41 | 11.91 | sig (♣) |
| GD0120_vs_GD0185 | 100 | 1 | 0.37 | 0.37 | 0.08 | ns |
| GD0180_vs_GD0185 | 100 | 1 | 68.14 | 68.14 | 13.90 | sig (♣) |
| Error |  | 68 | 333.38 | 4.90 |  |  |

^†^F_(0.05/2, 5, 68)_ = 2.76 (Family error rate critical value)

^‡^F_(0.35/10, 5, 68)_ = 2.56 (Dun’s critical value)

^♣^F_(0.5/15, 1, 33)_ = 10.01 (Family error rate critical value for simple effect contrasts and simple simple effect contrast between genotypes)

Table S6. Analysis of variance, simple interaction, simple effect, main effect contrasts and simple effect contrasts for effects of genotype, salt treatment and growth stage on *stomatal conductance* (Cond). 0 and 100: salt treatment at 0 and 100 mM NaCl, respectively.

| **Source** |  | **df** | **SS** | **MS** | **F** | **Sig** |
| --- | --- | --- | --- | --- | --- | --- |
| Genotype (G) |  | 5 | 0.78 | 0.16 | 18.33 | <0.001 |
| Treatment (T) |  | 1 | 1.55 | 1.55 | 182.12 | <0.001 |
| Growth stage (GS) |  | 1 | 7.21E-06 | 7.21E-06 | 0.00 | 0.977 |
| G x T |  | 5 | 0.26 | 0.05 | 6.16 | <0.001 |
| G x GS |  | 5 | 0.03 | 0.01 | 0.71 | 0.621 |
| T x GS |  | 1 | 2.66E-04 | 2.66E-04 | 0.03 | 0.860 |
| G x T x GS |  | 5 | 0.05 | 0.01 | 1.15 | 0.340 |
| ***Simple effect (G at each T)*** | | | | | |  |
| G at T = 0 |  | 5 | 0.94 | 1.88E-01 | 22.07 | sig († ‡) |
| G at T = 100 |  | 5 | 0.10 | 2.06E-02 | 2.42 | ns |
| ***Main effect contrasts*** |  |  |  |  |  |  |
| 0 vs 100 |  | 1 | 1.65 | 1.65 | 194.23 | sig († ‡) |
| ***Simple effect contrasts (Pairwise between genotypes at each treatment)*** | | | | | |  |
| *Pairs* | *Treatment* |  |  |  |  |  |
| Gladius_vs_Drysdale | 0 | 1 | 0.27 | 0.27 | 31.55 | sig (♣) |
| Gladius_vs_GD0014 | 0 | 1 | 0.01 | 0.01 | 1.71 | ns |
| Gladius_vs_GD0120 | 0 | 1 | 0.15 | 0.15 | 17.43 | sig (♣) |
| Gladius_vs_GD0180 | 0 | 1 | 6.90E-04 | 6.90E-04 | 0.08 | ns |
| Gladius_vs_GD0185 | 0 | 1 | 0.10 | 0.10 | 12.22 | sig (♣) |
| Drysdale_vs_GD0014 | 0 | 1 | 0.40 | 0.40 | 46.64 | sig (♣) |
| Drysdale_vs_GD0120 | 0 | 1 | 0.77 | 0.77 | 89.91 | sig (♣) |
| Drysdale_vs_GD0180 | 0 | 1 | 0.29 | 0.29 | 34.59 | sig (♣) |
| Drysdale_vs_GD0185 | 0 | 1 | 0.67 | 0.67 | 78.38 | sig (♣) |
| GD0014_vs_GD0120 | 0 | 1 | 0.07 | 0.07 | 8.21 | ns |
| GD0014_vs_GD0180 | 0 | 1 | 0.01 | 0.01 | 1.05 | ns |
| GD0014_vs_GD0185 | 0 | 1 | 0.04 | 0.04 | 4.78 | ns |
| GD0120_vs_GD0180 | 0 | 1 | 0.13 | 0.13 | 15.13 | sig (♣) |
| GD0120_vs_GD0185 | 0 | 1 | 3.93E-03 | 3.93E-03 | 0.46 | ns |
| GD0180_vs_GD0185 | 0 | 1 | 0.09 | 0.09 | 10.30 | sig (♣) |
| Gladius_vs_Drysdale | 100 | 1 | 0.02 | 0.02 | 2.15 | ns |
| Gladius_vs_GD0014 | 100 | 1 | 0.01 | 0.01 | 1.03 | ns |
| Gladius_vs_GD0120 | 100 | 1 | 0.01 | 0.01 | 1.68 | ns |
| Gladius_vs_GD0180 | 100 | 1 | 1.80E-04 | 1.80E-04 | 0.02 | ns |
| Gladius_vs_GD0185 | 100 | 1 | 0.01 | 0.01 | 1.48 | ns |
| Drysdale_vs_GD0014 | 100 | 1 | 2.38E-03 | 2.38E-03 | 0.28 | ns |
| Drysdale_vs_GD0120 | 100 | 1 | 0.06 | 0.06 | 7.11 | ns |
| Drysdale_vs_GD0180 | 100 | 1 | 0.02 | 0.02 | 2.57 | ns |
| Drysdale_vs_GD0185 | 100 | 1 | 0.06 | 0.06 | 6.73 | ns |
| GD0014_vs_GD0120 | 100 | 1 | 0.05 | 0.05 | 5.33 | ns |
| GD0014_vs_GD0180 | 100 | 1 | 0.01 | 0.01 | 1.35 | ns |
| GD0014_vs_GD0185 | 100 | 1 | 0.04 | 0.04 | 4.97 | ns |
| GD0120_vs_GD0180 | 100 | 1 | 0.01 | 0.01 | 1.32 | ns |
| GD0120_vs_GD0185 | 100 | 1 | 5.00E-05 | 5.00E-05 | 0.01 | ns |
| GD0180_vs_GD0185 | 100 | 1 | 0.01 | 0.01 | 1.14 | ns |
| Error |  | 68 | 0.58 | 0.009 |  |  |

^†^F_(0.05/2, 5, 68)_ = 2.76 (Family error rate critical value)

^‡^F_(0.35/10, 5, 68)_ = 2.56 (Dun’s critical value)

^♣^F_(0.5/15, 1, 33)_ = 10.01 (Family error rate critical value for simple effect contrasts and simple effect contrast between genotypes)

Table S7. Analysis of variance and main effect contrasts for effects of genotype, salt treatment and growth stage on *intercellular CO_2_ concentration* (Ci). 0 and 100: salt treatment at 0 and 100 mM NaCl, respectively.

| **Source** | **df** | **SS** | **MS** | **F** | **Sig** |
| --- | --- | --- | --- | --- | --- |
| Genotype (G) | 5 | 25135.19 | 5027.04 | 8.70 | <0.001 |
| Treatment (T) | 1 | 110595.04 | 110595.04 | 191.36 | <0.001 |
| Growth stage (GS) | 1 | 658.42 | 658.42 | 1.14 | 0.290 |
| G x T | 5 | 4981.08 | 996.22 | 1.72 | 0.141 |
| G x GS | 5 | 1259.29 | 251.86 | 0.44 | 0.822 |
| T x GS | 1 | 194.11 | 194.11 | 0.34 | 0.564 |
| G x T x GS | 5 | 1675.20 | 335.04 | 0.58 | 0.715 |
| ***Main effect contrast between treatments*** | | | | | |
| 0 vs 100 | 1 | 108726.00 | 108726.00 | 188.13 | <0.001 |
| ***Main effect contrasts (Pairwise between genotypes)*** | | | | |  |
| *Pairs* |  |  |  |  |  |
| Gladius_vs_Drysdale | 1 | 4810.00 | 4810.00 | 8.32 | ns |
| Gladius_vs_GD0014 | 1 | 443.00 | 443.00 | 0.77 | ns |
| Gladius_vs_GD0120 | 1 | 3695.00 | 3695.00 | 6.39 | ns |
| Gladius_vs_GD0180 | 1 | 13.00 | 13.00 | 0.02 | ns |
| Gladius_vs_GD0185 | 1 | 4603.00 | 4603.00 | 7.96 | ns |
| Drysdale_vs_GD0014 | 1 | 2486.00 | 2486.00 | 4.30 | ns |
| Drysdale_vs_GD0120 | 1 | 15784.00 | 15784.00 | 27.31 | sig (♣) |
| Drysdale_vs_GD0180 | 1 | 4354.00 | 4354.00 | 7.53 | ns |
| Drysdale_vs_GD0185 | 1 | 17469.00 | 17469.00 | 30.23 | sig (♣) |
| GD0014_vs_GD0120 | 1 | 6698.00 | 6698.00 | 11.59 | sig (♣) |
| GD0014_vs_GD0180 | 1 | 303.00 | 303.00 | 0.52 | ns |
| GD0014_vs_GD0185 | 1 | 7904.00 | 7904.00 | 13.68 | sig (♣) |
| GD0120_vs_GD0180 | 1 | 4151.00 | 4151.00 | 7.18 | ns |
| GD0120_vs_GD0185 | 1 | 50.00 | 50.00 | 0.09 | ns |
| GD0180_vs_GD0185 | 1 | 5111.00 | 5111.00 | 8.84 | sig (♣) |
| Error | 68 | 39300.00 | 577.94 |  |  |

^♣^F_(0.5/15, 1, 33)_ = 10.01 (Family error rate critical value for simple effect contrasts and simple simple effect contrast between genotypes)

Table S8. Analysis of variance, simple interaction, simple effect, main effect contrasts and simple effect contrasts for effects of genotype, salt treatment and growth stage on transpiration rate (E). 0 and 100: salt treatment at 0 and 100 mM NaCl, respectively.

| **Source** |  | **df** | **SS** | **MS** | **F** | **Sig** |
| --- | --- | --- | --- | --- | --- | --- |
| Genotype (G) |  | 5 | 6.10E-05 | 1.22E-05 | 18.87 | <0.001 |
| Treatment (T) |  | 1 | 1.29E-04 | 1.29E-04 | 199.67 | <0.001 |
| Growth stage (GS) |  | 1 | 3.01E-10 | 3.01E-10 | 0.00 | 0.983 |
| G x T |  | 5 | 1.77E-05 | 3.55E-06 | 5.49 | <0.001 |
| G x GS |  | 5 | 2.19E-06 | 4.37E-07 | 0.68 | 0.643 |
| T x GS |  | 1 | 3.16E-08 | 3.16E-08 | 0.05 | 0.826 |
| G x T x GS |  | 5 | 3.81E-06 | 7.62E-07 | 1.18 | 0.329 |
| ***Simple effect (G at each T)*** | | | | | | |
| G at T = 0 |  | 5 | 6.92E-05 | 1.38E-05 | 21.40 | sig († ‡) |
| G at T = 100 |  | 5 | 9.51E-06 | 1.90E-06 | 2.94 | sig († ‡) |
| ***Main effect contrasts*** | | | | | | |
| 0 vs 100 |  | 1 | 1.37E-04 | 1.37E-04 | 211.10 | <0.001 |
| ***Simple effect contrasts (Pairwise between genotypes at each treatment)*** | | | | | |  |
| *Pairs* | *Treatment* |  |  |  |  |  |
| Gladius_vs_Drysdale | 0 | 1 | 1.88E-05 | 1.88E-05 | 29.02 | sig (♣) |
| Gladius_vs_GD0014 | 0 | 1 | 9.56E-07 | 9.56E-07 | 1.48 | ns |
| Gladius_vs_GD0120 | 0 | 1 | 1.18E-05 | 1.18E-05 | 18.23 | sig (♣) |
| Gladius_vs_GD0180 | 0 | 1 | 7.70E-08 | 7.70E-08 | 0.12 | ns |
| Gladius_vs_GD0185 | 0 | 1 | 8.20E-06 | 8.20E-06 | 12.68 | sig (♣) |
| Drysdale_vs_GD0014 | 0 | 1 | 2.74E-05 | 2.74E-05 | 42.41 | sig (♣) |
| Drysdale_vs_GD0120 | 0 | 1 | 5.64E-05 | 5.64E-05 | 87.24 | sig (♣) |
| Drysdale_vs_GD0180 | 0 | 1 | 2.11E-05 | 2.11E-05 | 32.57 | sig (♣) |
| Drysdale_vs_GD0185 | 0 | 1 | 4.88E-05 | 4.88E-05 | 75.40 | sig (♣) |
| GD0014_vs_GD0120 | 0 | 1 | 6.04E-06 | 6.04E-06 | 9.33 | sig (♣) |
| GD0014_vs_GD0180 | 0 | 1 | 4.89E-07 | 4.89E-07 | 0.76 | ns |
| GD0014_vs_GD0185 | 0 | 1 | 3.56E-06 | 3.56E-06 | 5.50 | ns |
| GD0120_vs_GD0180 | 0 | 1 | 9.96E-06 | 9.96E-06 | 15.40 | sig (♣) |
| GD0120_vs_GD0185 | 0 | 1 | 3.26E-07 | 3.26E-07 | 0.50 | ns |
| GD0180_vs_GD0185 | 0 | 1 | 6.69E-06 | 6.69E-06 | 10.33 | sig (♣) |
| Gladius_vs_Drysdale | 100 | 1 | 1.64E-06 | 1.64E-06 | 2.53 | ns |
| Gladius_vs_GD0014 | 100 | 1 | 7.90E-07 | 7.90E-07 | 1.22 | ns |
| Gladius_vs_GD0120 | 100 | 1 | 1.37E-06 | 1.37E-06 | 2.11 | ns |
| Gladius_vs_GD0180 | 100 | 1 | 2.40E-08 | 2.40E-08 | 0.04 | ns |
| Gladius_vs_GD0185 | 100 | 1 | 1.18E-06 | 1.18E-06 | 1.83 | ns |
| Drysdale_vs_GD0014 | 100 | 1 | 2.08E-07 | 2.08E-07 | 0.32 | ns |
| Drysdale_vs_GD0120 | 100 | 1 | 5.58E-06 | 5.58E-06 | 8.62 | ns |
| Drysdale_vs_GD0180 | 100 | 1 | 2.03E-06 | 2.03E-06 | 3.13 | ns |
| Drysdale_vs_GD0185 | 100 | 1 | 5.23E-06 | 5.23E-06 | 8.08 | ns |
| GD0014_vs_GD0120 | 100 | 1 | 6.04E-06 | 6.04E-06 | 9.33 | sig (♣) |
| GD0014_vs_GD0180 | 100 | 1 | 1.09E-06 | 1.09E-06 | 1.68 | ns |
| GD0014_vs_GD0185 | 100 | 1 | 3.91E-06 | 3.91E-06 | 6.04 | ns |
| GD0120_vs_GD0180 | 100 | 1 | 1.03E-06 | 1.03E-06 | 1.59 | ns |
| GD0120_vs_GD0185 | 100 | 1 | 6.00E-09 | 6.00E-09 | 0.01 | ns |
| GD0180_vs_GD0185 | 100 | 1 | 8.71E-07 | 8.71E-07 | 1.35 | ns |
| Error |  | 68 | 4.40E-05 | 6.47E-07 |  |  |

Table S9. Summary results of effect of growth stage, genotype and salt treatment on photosynthetic rate (Pn), Stomatal conductance (Cond), intracellular CO_2_ concentration and transpiration rate (E). 0 and 100: salt treatment at 0 and 100 mM NaCl, respectively; 35 and 42 were 35 and 42 days after sowing. The results are mean ± standard deviation.

| **growth stage** | **genotype** | **treatment** | **Pn** | **Cond** | **Ci** | **E** |
| --- | --- | --- | --- | --- | --- | --- |
| ***Effect of treatment (main effect)*** | | |  |  |  |  |
|  |  | 0 | 21.25 ± 3.16 | 0.45 ± 0.19 | 304.39 ± 24.44 | 0.0042 ± 0.0016 |
|  |  | 100 | 17.18 ± 3.14 | 0.19 ± 0.06 | 235.05 ± 32.08 | 0.0018 ± 0.0006 |
| ***Effect of genotype (main effect)*** | | |  |  |  |  |
|  | Gladius |  | 20.68 ± 3.66 | 0.34 ± 0.17 | 272.65 ± 41.14 | 0.0032 ± 0.0016 |
|  | Drysdale |  | 22.52 ± 2.67 | 0.51 ± 0.3 | 299.14 ± 36.89 | 0.0047 ± 0.0026 |
|  | GD0014 |  | 20.67 ± 2.69 | 0.33 ± 0.12 | 280.1 ± 29.37 | 0.0032 ± 0.0011 |
|  | GD0120 |  | 16.18 ± 3.77 | 0.21 ± 0.1 | 251.16 ± 41.8 | 0.0021 ± 0.0009 |
|  | GD0180 |  | 18.64 ± 2.56 | 0.33 ± 0.18 | 273.94 ± 50.64 | 0.0031 ± 0.0016 |
|  | GD0185 |  | 17.42 ± 3.39 | 0.23 ± 0.1 | 248.67 ± 52.08 | 0.0022 ± 0.001 |
| ***Genotype x salt treatment*** | |  |  |  |  |  |
|  | Gladius | 0 | 23.23 ± 3.54 | 0.48 ± 0.12 | 308.94 ± 16.85 | 0.0046 ± 0.001 |
|  | Drysdale | 0 | 24.81 ± 1.64 | 0.76 ± 0.22 | 331.51 ± 20.89 | 0.0069 ± 0.0019 |
|  | GD0014 | 0 | 21.99 ± 2.87 | 0.42 ± 0.1 | 302 ± 22.29 | 0.0041 ± 0.0009 |
|  | GD0120 | 0 | 19.1 ± 1.85 | 0.29 ± 0.06 | 282.6 ± 16.19 | 0.0028 ± 0.0005 |
|  | GD0180 | 0 | 19.77 ± 2.09 | 0.47 ± 0.15 | 315.86 ± 25.71 | 0.0044 ± 0.0013 |
|  | GD0185 | 0 | 19.47 ± 2.54 | 0.32 ± 0.04 | 292.2 ± 17.37 | 0.0031 ± 0.0004 |
|  | Gladius | 100 | 18.12 ± 1.1 | 0.19 ± 0.02 | 236.36 ± 18.24 | 0.0019 ± 0.0002 |
|  | Drysdale | 100 | 20.22 ± 0.63 | 0.26 ± 0.02 | 266.77 ± 6.49 | 0.0026 ± 0.0002 |
|  | GD0014 | 100 | 19.35 ± 1.82 | 0.24 ± 0.03 | 258.2 ± 15.99 | 0.0023 ± 0.0003 |
|  | GD0120 | 100 | 13.27 ± 2.75 | 0.13 ± 0.05 | 219.73 ± 34.99 | 0.0013 ± 0.0005 |
|  | GD0180 | 100 | 17.51 ± 2.6 | 0.18 ± 0.05 | 232.02 ± 28.59 | 0.0018 ± 0.0005 |
|  | GD0185 | 100 | 15.36 ± 2.91 | 0.13 ± 0.03 | 205.13 ± 34.32 | 0.0013 ± 0.0003 |
| ***Growth stage x genotype*** | |  |  |  |  |  |
| 35 | Gladius |  | 20.62 ± 3.49 | 0.35 ± 0.2 | 270.12 ± 51.24 | 0.0033 ± 0.0018 |
| 35 | Drysdale |  | 23.08 ± 3.04 | 0.49 ± 0.3 | 297.19 ± 34.39 | 0.0046 ± 0.0026 |
| 35 | GD0014 |  | 20.1 ± 1.99 | 0.31 ± 0.12 | 275.75 ± 34.77 | 0.003 ± 0.0011 |
| 35 | GD0120 |  | 15.95 ± 3.54 | 0.2 ± 0.09 | 244.68 ± 42.05 | 0.0019 ± 0.0009 |
| 35 | GD0180 |  | 18.62 ± 3.42 | 0.36 ± 0.22 | 278.64 ± 54.05 | 0.0034 ± 0.002 |
| 35 | GD0185 |  | 18.72 ± 2.49 | 0.23 ± 0.09 | 243.42 ± 52.84 | 0.0022 ± 0.0009 |
| 42 | Gladius |  | 20.73 ± 4.06 | 0.33 ± 0.16 | 275.18 ± 31.41 | 0.0031 ± 0.0014 |
| 42 | Drysdale |  | 21.96 ± 2.38 | 0.53 ± 0.34 | 301.09 ± 42.45 | 0.0049 ± 0.0028 |
| 42 | GD0014 |  | 21.24 ± 3.29 | 0.35 ± 0.12 | 284.45 ± 24.42 | 0.0034 ± 0.0012 |
| 42 | GD0120 |  | 16.41 ± 4.21 | 0.22 ± 0.11 | 257.65 ± 43.36 | 0.0022 ± 0.001 |
| 42 | GD0180 |  | 18.66 ± 1.54 | 0.3 ± 0.15 | 269.25 ± 50.24 | 0.0028 ± 0.0013 |
| 42 | GD0185 |  | 16.12 ± 3.81 | 0.23 ± 0.12 | 253.91 ± 54.38 | 0.0022 ± 0.0011 |
| ***Growth stage x genotype x treatment*** | | |  |  |  |  |
| 35 | Gladius | 0 | 23.18 ± 3.07 | 0.52 ± 0.09 | 315.61 ± 18.97 | 0.0049 ± 0.0008 |
| 35 | Drysdale | 0 | 25.72 ± 1.35 | 0.72 ± 0.27 | 323.83 ± 27.95 | 0.0065 ± 0.0023 |
| 35 | GD0014 | 0 | 20.03 ± 2.21 | 0.39 ± 0.13 | 300.06 ± 31.82 | 0.0038 ± 0.0012 |
| 35 | GD0120 | 0 | 19.01 ± 1.17 | 0.27 ± 0.05 | 277.31 ± 15.84 | 0.0027 ± 0.0005 |
| 35 | GD0180 | 0 | 21.27 ± 1.72 | 0.54 ± 0.14 | 320.81 ± 27.21 | 0.005 ± 0.0012 |
| 35 | GD0185 | 0 | 19.93 ± 1.99 | 0.31 ± 0.04 | 286.55 ± 9.57 | 0.003 ± 0.0004 |
| 35 | Gladius | 100 | 18.06 ± 1.18 | 0.17 ± 0.02 | 224.64 ± 15.76 | 0.0017 ± 0.0002 |
| 35 | Drysdale | 100 | 20.43 ± 0.57 | 0.27 ± 0.01 | 270.56 ± 6.91 | 0.0027 ± 0.0001 |
| 35 | GD0014 | 100 | 20.18 ± 2.09 | 0.23 ± 0.02 | 251.43 ± 15.23 | 0.0023 ± 0.0002 |
| 35 | GD0120 | 100 | 12.9 ± 1.71 | 0.12 ± 0.03 | 212.04 ± 32.17 | 0.0012 ± 0.0003 |
| 35 | GD0180 | 100 | 15.97 ± 2.37 | 0.18 ± 0.08 | 236.47 ± 36.52 | 0.0017 ± 0.0007 |
| 35 | GD0185 | 100 | 17.51 ± 2.56 | 0.15 ± 0.03 | 200.28 ± 38.23 | 0.0015 ± 0.0003 |
| 42 | Gladius | 0 | 23.28 ± 4.44 | 0.45 ± 0.14 | 302.27 ± 13.57 | 0.0042 ± 0.0012 |
| 42 | Drysdale | 0 | 23.9 ± 1.55 | 0.81 ± 0.22 | 339.2 ± 11.52 | 0.0073 ± 0.0017 |
| 42 | GD0014 | 0 | 23.95 ± 2.01 | 0.45 ± 0.06 | 303.94 ± 11.69 | 0.0044 ± 0.0005 |
| 42 | GD0120 | 0 | 19.18 ± 2.57 | 0.31 ± 0.07 | 287.88 ± 16.91 | 0.003 ± 0.0006 |
| 42 | GD0180 | 0 | 18.26 ± 1.13 | 0.4 ± 0.14 | 310.92 ± 27.15 | 0.0038 ± 0.0012 |
| 42 | GD0185 | 0 | 19.02 ± 3.25 | 0.34 ± 0.05 | 297.86 ± 22.95 | 0.0032 ± 0.0004 |
| 42 | Gladius | 100 | 18.19 ± 1.18 | 0.21 ± 0.02 | 248.09 ± 12.7 | 0.002 ± 0.0002 |
| 42 | Drysdale | 100 | 20.02 ± 0.73 | 0.25 ± 0.01 | 262.97 ± 3.8 | 0.0025 ± 0.0001 |
| 42 | GD0014 | 100 | 18.52 ± 1.23 | 0.24 ± 0.05 | 264.96 ± 15.57 | 0.0024 ± 0.0004 |
| 42 | GD0120 | 100 | 13.64 ± 3.79 | 0.14 ± 0.06 | 227.42 ± 40.78 | 0.0014 ± 0.0006 |
| 42 | GD0180 | 100 | 19.05 ± 1.97 | 0.19 ± 0.02 | 227.57 ± 22.83 | 0.0018 ± 0.0001 |
| 42 | GD0185 | 100 | 13.21 ± 0.93 | 0.12 ± 0.02 | 209.97 ± 34.99 | 0.0012 ± 0.0002 |

Table S10. Effect of genotype and treatment on OJIP parameters at 41 days after sowing (DAS). The results were the mean ± standard deviation (n=4). Different letters show significantly between genotype within a column (P < 0.05); no letter or the same letter shows no significant difference. The definitions of parameters were described in FluorPen manual (https://handheld.psi.cz/documents/FP_manual-new%20format-vs1.0-edit-2021.pdf).

| **genotype** | **treatment** | Fv/Fm | Mo | Sm | Ss | N |
| --- | --- | --- | --- | --- | --- | --- |
| ***Effect of treatment (main effect)*** | |  |  |  |  |  |
|  | 0 | 0.83 ± 0.006 | 0.68 ± 0.056^a^ | 428 ± 42 | 0.55 ± 0.02 | 773 ± 72 |
|  | 100 | 0.83 ± 0.006 | 0.65 ± 0.044^b^ | 429 ± 48 | 0.56 ± 0.03 | 766 ± 75 |
| ***Effect of genotype (main effect)*** | |  |  |  |  |  |
| Gladius |  | 0.83 ± 0.005 | 0.65 ± 0.048 | 450 ± 51 | 0.57 ± 0.03^a^ | 782 ± 63 |
| Drysdale |  | 0.83 ± 0.004 | 0.65 ± 0.08 | 436 ± 42 | 0.56 ± 0.03 | 773 ± 72 |
| GD0014 |  | 0.82 ± 0.005 | 0.7 ± 0.041 | 439 ± 58 | 0.54 ± 0.02^b^ | 816 ± 102 |
| GD0120 |  | 0.83 ± 0.006 | 0.63 ± 0.014 | 416 ± 39 | 0.56 ± 0.01 | 738 ± 66 |
| GD0180 |  | 0.83 ± 0.007 | 0.67 ± 0.063 | 427 ± 40 | 0.55 ± 0.03 | 775 ± 63 |
| GD0185 |  | 0.83 ± 0.008 | 0.67 ± 0.029 | 403 ± 28 | 0.55 ± 0.01 | 732 ± 46 |
| ***Genotype x salt treatment*** | |  |  |  |  |  |
| Gladius | 0 | 0.83 ± 0.006 | 0.66 ± 0.022 | 452 ± 40 | 0.58 ± 0.01 | 786 ± 62 |
| Drysdale | 0 | 0.83 ± 0.005 | 0.67 ± 0.107 | 450 ± 40 | 0.56 ± 0.03 | 810 ± 55 |
| GD0014 | 0 | 0.82 ± 0.005 | 0.72 ± 0.047 | 423 ± 45 | 0.54 ± 0.02 | 784 ± 101 |
| GD0120 | 0 | 0.83 ± 0.005 | 0.64 ± 0.009 | 408 ± 51 | 0.57 ± 0.01 | 721 ± 88 |
| GD0180 | 0 | 0.83 ± 0.006 | 0.7 ± 0.06 | 429 ± 46 | 0.54 ± 0.02 | 799 ± 72 |
| GD0185 | 0 | 0.82 ± 0.008 | 0.68 ± 0.026 | 407 ± 29 | 0.55 ± 0.02 | 741 ± 42 |
| Gladius | 100 | 0.83 ± 0.003 | 0.65 ± 0.07 | 449 ± 67 | 0.57 ± 0.04 | 778 ± 73 |
| Drysdale | 100 | 0.83 ± 0.002 | 0.63 ± 0.049 | 421 ± 44 | 0.57 ± 0.02 | 736 ± 73 |
| GD0014 | 100 | 0.82 ± 0.005 | 0.68 ± 0.028 | 455 ± 72 | 0.53 ± 0.02 | 849 ± 104 |
| GD0120 | 100 | 0.83 ± 0.007 | 0.63 ± 0.014 | 423 ± 28 | 0.56 ± 0.01 | 755 ± 43 |
| GD0180 | 100 | 0.83 ± 0.008 | 0.64 ± 0.058 | 426 ± 41 | 0.57 ± 0.03 | 752 ± 51 |
| GD0185 | 100 | 0.83 ± 0.005 | 0.65 ± 0.028 | 400 ± 31 | 0.55 ± 0.01 | 723 ± 55 |
| ***Anova analysis (P value)*** | |  |  |  |  |  |
| Genotype (G) |  | 0.094 | 0.214 | 0.393 | 0.043 | 0.209 |
| Treatment (T) |  | 0.191 | 0.044 | 0.963 | 0.386 | 0.704 |
| G x T |  | 0.33 | 0.923 | 0.847 | 0.559 | 0.408 |

Table S10. (continuing). Effect of genotype and treatment on OJIP parameters at 41 days after sowing (DAS). The results were the mean ± standard deviation (n=4) for genotype x treatment. Different letters show significantly between genotype within a column (P < 0.05); no letter or the same letter shows no significant difference. The definitions of parameters were described described in FluorPen manual (https://handheld.psi.cz/documents/FP_manual-new%20format-vs1.0-edit-2021.pdf).

| **genotype** | **treatment** | Phi_Po | Psi_o | Phi_Eo | Phi_Do | Phi_Pav |
| --- | --- | --- | --- | --- | --- | --- |
| ***Effect of treatment (main effect)*** | |  |  |  |  |  |
|  | 0 | 0.83 ± 0.006 | 0.63 ± 0.019^a^ | 0.52 ± 0.018 | 0.17 ± 0.006 | 939 ± 6 |
|  | 100 | 0.83 ± 0.006 | 0.64 ± 0.013^b^ | 0.53 ± 0.012 | 0.17 ± 0.006 | 937 ± 7 |
| ***Effect of genotype (main effect)*** | |  |  |  |  |  |
| Gladius |  | 0.83 ± 0.005 | 0.63 ± 0.014 | 0.52 ± 0.011 | 0.17 ± 0.005 | 938 ± 7 |
| Drysdale |  | 0.83 ± 0.004 | 0.64 ± 0.028 | 0.53 ± 0.025 | 0.17 ± 0.004 | 939 ± 6 |
| GD0014 |  | 0.82 ± 0.005 | 0.63 ± 0.018 | 0.52 ± 0.016 | 0.18 ± 0.005 | 938 ± 8 |
| GD0120 |  | 0.83 ± 0.006 | 0.64 ± 0.009 | 0.53 ± 0.009 | 0.17 ± 0.006 | 941 ± 6 |
| GD0180 |  | 0.83 ± 0.007 | 0.63 ± 0.019 | 0.52 ± 0.018 | 0.17 ± 0.007 | 935 ± 8 |
| GD0185 |  | 0.83 ± 0.008 | 0.63 ± 0.011 | 0.52 ± 0.013 | 0.17 ± 0.008 | 939 ± 6 |
| ***Genotype x salt treatment*** | |  |  |  |  |  |
| Gladius | 0 | 0.83 ± 0.006 | 0.62 ± 0.014 | 0.52 ± 0.013 | 0.17 ± 0.006 | 937 ± 7 |
| Drysdale | 0 | 0.83 ± 0.005 | 0.63 ± 0.038 | 0.52 ± 0.034 | 0.17 ± 0.005 | 941 ± 5 |
| GD0014 | 0 | 0.82 ± 0.005 | 0.61 ± 0.013 | 0.5 ± 0.014 | 0.18 ± 0.005 | 937 ± 7 |
| GD0120 | 0 | 0.83 ± 0.005 | 0.64 ± 0.005 | 0.53 ± 0.003 | 0.17 ± 0.005 | 942 ± 6 |
| GD0180 | 0 | 0.83 ± 0.006 | 0.63 ± 0.02 | 0.52 ± 0.019 | 0.17 ± 0.006 | 937 ± 9 |
| GD0185 | 0 | 0.82 ± 0.008 | 0.63 ± 0.01 | 0.52 ± 0.011 | 0.18 ± 0.008 | 938 ± 7 |
| Gladius | 100 | 0.83 ± 0.003 | 0.63 ± 0.015 | 0.52 ± 0.011 | 0.17 ± 0.003 | 938 ± 7 |
| Drysdale | 100 | 0.83 ± 0.002 | 0.64 ± 0.018 | 0.54 ± 0.016 | 0.17 ± 0.002 | 937 ± 8 |
| GD0014 | 100 | 0.82 ± 0.005 | 0.64 ± 0.011 | 0.53 ± 0.008 | 0.18 ± 0.005 | 939 ± 10 |
| GD0120 | 100 | 0.83 ± 0.007 | 0.65 ± 0.007 | 0.54 ± 0.009 | 0.17 ± 0.007 | 939 ± 5 |
| GD0180 | 100 | 0.83 ± 0.008 | 0.64 ± 0.017 | 0.53 ± 0.017 | 0.17 ± 0.008 | 932 ± 7 |
| GD0185 | 100 | 0.83 ± 0.005 | 0.64 ± 0.007 | 0.53 ± 0.009 | 0.17 ± 0.005 | 939 ± 5 |
| ***Anova analysis (P value)*** | |  |  |  |  |  |
| Genotype (G) |  | 0.094 | 0.327 | 0.278 | 0.094 | 0.662 |
| Treatment (T) |  | 0.191 | 0.01 | 0.011 | 0.191 | 0.434 |
| G x T |  | 0.33 | 0.914 | 0.891 | 0.33 | 0.878 |

Table S10 (continuing). Effect of genotype and treatment on OJIP parameters at 41 days after sowing (DAS). The results were the mean ± standard deviation (n=4) for genotype x treatment. Different letters show significantly between genotype within a column (P < 0.05); no letter or the same letter shows no significant difference. The definition of parameters were described in FluorPen manual (https://handheld.psi.cz/documents/FP_manual-new%20format-vs1.0-edit-2021.pdf).

| **genotype** | **treatment** | Pi_Abs | ABS/RC | TRo/RC | ETo/RC | DIo/RC |
| --- | --- | --- | --- | --- | --- | --- |
| ***Effect of treatment (main effect)*** | | |  |  |  |  |
|  | 0 | 3.73 ± 0.52^a^ | 2.19 ± 0.11 | 1.81 ± 0.078 | 1.13 ± 0.045 | 0.38 ± 0.03 |
|  | 100 | 4.05 ± 0.46^b^ | 2.16 ± 0.1 | 1.79 ± 0.081 | 1.14 ± 0.047 | 0.37 ± 0.026 |
| ***Effect of genotype (main effect)*** | |  |  |  |  |  |
| Gladius |  | 3.97 ± 0.39 | 2.1 ± 0.1 | 1.74 ± 0.085^a^ | 1.09 ± 0.045^a^ | 0.35 ± 0.019^a^ |
| Drysdale |  | 4.11 ± 0.71 | 2.14 ± 0.11 | 1.78 ± 0.083 | 1.13 ± 0.025 | 0.36 ± 0.028 |
| GD0014 |  | 3.48 ± 0.38 | 2.26 ± 0.1 | 1.86 ± 0.076^b^ | 1.16 ± 0.061^b^ | 0.4 ± 0.026^b^ |
| GD0120 |  | 4.11 ± 0.27 | 2.14 ± 0.04 | 1.78 ± 0.034 | 1.14 ± 0.033 | 0.36 ± 0.017 |
| GD0180 |  | 3.87 ± 0.61 | 2.19 ± 0.12 | 1.82 ± 0.094 | 1.15 ± 0.042 | 0.37 ± 0.033 |
| GD0185 |  | 3.8 ± 0.43 | 2.2 ± 0.08 | 1.82 ± 0.048 | 1.15 ± 0.032 | 0.38 ± 0.029 |
| ***Genotype x salt treatment*** | |  |  |  |  |  |
| Gladius | 0 | 3.99 ± 0.35 | 2.09 ± 0.05 | 1.74 ± 0.035 | 1.08 ± 0.039 | 0.35 ± 0.018 |
| Drysdale | 0 | 3.91 ± 0.87 | 2.18 ± 0.14 | 1.8 ± 0.106 | 1.14 ± 0.035 | 0.37 ± 0.034 |
| GD0014 | 0 | 3.31 ± 0.42 | 2.24 ± 0.1 | 1.85 ± 0.08 | 1.13 ± 0.044 | 0.4 ± 0.027 |
| GD0120 | 0 | 3.99 ± 0.18 | 2.13 ± 0.06 | 1.77 ± 0.041 | 1.13 ± 0.034 | 0.36 ± 0.021 |
| GD0180 | 0 | 3.65 ± 0.58 | 2.25 ± 0.12 | 1.87 ± 0.086 | 1.17 ± 0.049 | 0.39 ± 0.035 |
| GD0185 | 0 | 3.56 ± 0.38 | 2.22 ± 0.08 | 1.83 ± 0.056 | 1.15 ± 0.042 | 0.39 ± 0.031 |
| Gladius | 100 | 3.94 ± 0.47 | 2.11 ± 0.14 | 1.75 ± 0.125 | 1.1 ± 0.056 | 0.36 ± 0.019 |
| Drysdale | 100 | 4.31 ± 0.55 | 2.1 ± 0.07 | 1.75 ± 0.055 | 1.12 ± 0.01 | 0.35 ± 0.016 |
| GD0014 | 100 | 3.65 ± 0.27 | 2.28 ± 0.11 | 1.88 ± 0.081 | 1.2 ± 0.064 | 0.4 ± 0.029 |
| GD0120 | 100 | 4.24 ± 0.3 | 2.15 ± 0.02 | 1.79 ± 0.028 | 1.16 ± 0.023 | 0.36 ± 0.014 |
| GD0180 | 100 | 4.09 ± 0.64 | 2.13 ± 0.11 | 1.77 ± 0.081 | 1.13 ± 0.027 | 0.36 ± 0.032 |
| GD0185 | 100 | 4.05 ± 0.35 | 2.18 ± 0.07 | 1.81 ± 0.047 | 1.16 ± 0.022 | 0.37 ± 0.023 |
| ***Anova analysis (P value)*** | |  |  |  |  |  |
| Genotype (G) |  | 0.115 | 0.031 | 0.044 | 0.015 | 0.023 |
| Treatment (T) |  | 0.03 | 0.316 | 0.383 | 0.311 | 0.201 |
| G x T |  | 0.899 | 0.556 | 0.509 | 0.165 | 0.564 |

Table S11. Effect of genotype and treatment on leaf mineral concentration at 45 days after sowing (DAS). The results were the mean ± standard deviation (n=4) for genotype x treatment.

| **genotype** | **treatment** | B (mg/kg) | Ca (g/kg) | Cu (mg/kg) | Fe (mg/kg) | K (g/kg) | Mg (g/kg) |
| --- | --- | --- | --- | --- | --- | --- | --- |
| ***Effect of treatment (main effect)*** | | |  |  |  |  |  |
|  | 0 | 26.5 ± 9.9 | 2.84 ± 1.1 | 14.3 ± 11.7 | 77.7 ± 12.1 | 47.3 ± 5.2 | 1.38 ± 0.32 |
|  | 100 | 19.3 ± 7 | 4.38 ± 1.12 | 12.8 ± 6.2 | 82.9 ± 10.6 | 43.1 ± 3.4 | 1.81 ± 0.27 |
| ***Effect of genotype (main effect)*** | | |  |  |  |  |  |
| Gladius |  | 17.8 ± 6.3 | 3.01 ± 1.03 | 15.2 ± 5.2 | 81.2 ± 14 | 47.4 ± 3.6 | 1.41 ± 0.24 |
| Drysdale |  | 29.7 ± 7.7 | 4.06 ± 1.13 | 18.2 ± 8.6 | 84.2 ± 7.7 | 39.7 ± 5.8 | 1.72 ± 0.2 |
| GD0014 |  | 22.9 ± 10.6 | 3.72 ± 2.2 | 20.2 ± 18.4 | 81.9 ± 13.1 | 46.9 ± 5.1 | 1.66 ± 0.49 |
| GD0120 |  | 19.6 ± 9.4 | 3.22 ± 1.24 | 8.9 ± 1.4 | 72.2 ± 13.9 | 44 ± 2.8 | 1.41 ± 0.37 |
| GD0180 |  | 28.7 ± 9.8 | 4.17 ± 0.52 | 9.8 ± 0.3 | 84.7 ± 6.2 | 46.6 ± 3 | 1.86 ± 0.16 |
| GD0185 |  | 21.6 ± 7.7 | 3.56 ± 1.29 | 9.6 ± 0.8 | 78.8 ± 9.3 | 45.5 ± 5.5 | 1.55 ± 0.43 |
| ***Genotype x salt treatment*** | |  | 19.2 ± 9 | 2.26 ± 0.9 | 12.2 ± 4.8 | 73.2 ± 6.3 | 50.5 ± 1.3 |
| Gladius | 0 | 30 ± 7.5 | 3.25 ± 0.64 | 16 ± 3 | 88.7 ± 2.3 | 42.1 ± 8.1 | 1.63 ± 0.23 |
| Drysdale | 0 | 24.5 ± 12.7 | 2.7 ± 1.7 | 30.7 ± 22.2 | 81.2 ± 9.5 | 49.8 ± 4.9 | 1.4 ± 0.35 |
| GD0014 | 0 | 25.5 ± 10.5 | 2.55 ± 1.25 | 8 ± 1.2 | 64.8 ± 15.4 | 44.2 ± 3 | 1.15 ± 0.3 |
| GD0120 | 0 | 35 ± 8.3 | 3.9 ± 0.56 | 9.9 ± 0.2 | 87.8 ± 5.9 | 48.2 ± 2.6 | 1.77 ± 0.17 |
| GD0180 | 0 | 25.8 ± 8.8 | 2.48 ± 0.76 | 9.2 ± 1 | 73.5 ± 10.8 | 47.4 ± 6.9 | 1.18 ± 0.2 |
| GD0185 | 0 |  |  |  |  |  |  |
| Gladius | 100 | 16.2 ± 2.5 | 3.76 ± 0.44 | 18.2 ± 3.9 | 89.2 ± 15.8 | 44.3 ± 1.6 | 1.6 ± 0.12 |
| Drysdale | 100 | 29.3 ± 9.5 | 4.87 ± 0.93 | 20.3 ± 12.7 | 79.7 ± 9.1 | 37.4 ± 0.9 | 1.8 ± 0.17 |
| GD0014 | 100 | 21.2 ± 9.6 | 4.73 ± 2.38 | 9.8 ± 0.9 | 82.5 ± 17.5 | 43.9 ± 3.7 | 1.93 ± 0.51 |
| GD0120 | 100 | 13.8 ± 1.9 | 3.89 ± 0.92 | 9.8 ± 1.1 | 79.8 ± 7.9 | 43.9 ± 3 | 1.68 ± 0.22 |
| GD0180 | 100 | 20.3 ± 1.2 | 4.52 ± 0.14 | 9.7 ± 0.5 | 80.7 ± 4.5 | 44.4 ± 2 | 1.97 ± 0.06 |
| GD0185 | 100 | 17.5 ± 4 | 4.64 ± 0.44 | 10 ± 0 | 84 ± 3.7 | 43.5 ± 3.7 | 1.93 ± 0.17 |
| ***Anova analysis (P value)*** | |  |  |  |  |  |  |
| Genotype (G) |  | 0.049 | 0.330 | 0.023 | 0.242 | 0.015 | 0.011 |
| Treatment (T) |  | 0.006 | <0.001 | 0.478 | 0.099 | 0.001 | <0.001 |
| G x T |  | 0.540 | 0.816 | 0.018 | 0.115 | 0.739 | 0.259 |

Table S11 (continuing). Effect of genotype and treatment on leaf mineral concentration at 45 days after sowing (DAS). The results were the mean ± standard deviation (n=4) for genotype x treatment.

| **genotype** | **treatment** | Mn (mg/kg) | Na (g/kg) | P (g/kg) | S (g/kg) | Zn (mg/kg) | K/Na (mM/mM) |
| --- | --- | --- | --- | --- | --- | --- | --- |
| ***Effect of treatment (main effect)*** | | |  |  |  |  |  |
|  | 0 | 55.3 ± 12.3 | 0.22 ± 0.2 | 6.51 ± 0.74 | 4.63 ± 0.65 | 41 ± 8.7 | 239.2 ± 162.4 |
|  | 100 | 73.5 ± 14.7 | 2.09 ± 0.95 | 6.04 ± 0.73 | 4.05 ± 0.4 | 52.6 ± 9.6 | 16.8 ± 12.4 |
| ***Effect of genotype (main effect)*** | | |  |  |  |  |  |
| Gladius |  | 58.6 ± 10.1 | 0.7 ± 0.67 | 6.32 ± 0.35 | 4.74 ± 0.73 | 44.5 ± 8.4 | 185 ± 185 |
| Drysdale |  | 76.5 ± 18.1 | 0.3 ± 0.28 | 6.03 ± 0.94 | 4.48 ± 0.62 | 41.8 ± 7.8 | 247.9 ± 237.7 |
| GD0014 |  | 67 ± 22.1 | 1.48 ± 1.49 | 6.38 ± 0.62 | 4.49 ± 0.42 | 48.9 ± 11.2 | 103.3 ± 101.7 |
| GD0120 |  | 59.5 ± 19 | 1.01 ± 1.18 | 6.21 ± 0.87 | 3.79 ± 0.4 | 42.9 ± 16.1 | 211 ± 198.3 |
| GD0180 |  | 63.9 ± 2.9 | 1.3 ± 0.97 | 6.37 ± 1.05 | 4.66 ± 0.5 | 52.1 ± 4.1 | 36 ± 24.2 |
| GD0185 |  | 62.6 ± 16.3 | 1.7 ± 1.43 | 6.3 ± 0.87 | 4.01 ± 0.44 | 49.4 ± 11.7 | 41.3 ± 36.3 |
| ***Genotype x salt treatment*** | | |  | 50 ± 2.8 | 0.09 ± 0.03 | 6.45 ± 0.24 | 5.35 ± 0.45 |
| Gladius | 0 | 69 ± 8.7 | 0.06 ± 0.03 | 5.9 ± 0.78 | 4.97 ± 0.47 | 44.7 ± 9.3 | 453.1 ± 121.5 |
| Drysdale | 0 | 55.2 ± 15.6 | 0.15 ± 0.01 | 6.5 ± 0.78 | 4.45 ± 0.39 | 40.8 ± 8.2 | 196.9 ± 28.1 |
| GD0014 | 0 | 48 ± 16 | 0.08 ± 0.02 | 6.4 ± 0.42 | 4.05 ± 0.42 | 32 ± 5.5 | 360.8 ± 93.9 |
| GD0120 | 0 | 63 ± 3.5 | 0.54 ± 0.14 | 7.18 ± 0.34 | 5.03 ± 0.22 | 52.5 ± 4.5 | 54.5 ± 10.6 |
| GD0180 | 0 | 50 ± 11.5 | 0.39 ± 0.09 | 6.47 ± 1.28 | 4.03 ± 0.64 | 39.8 ± 7.8 | 73.9 ± 15.3 |
| GD0185 | 0 |  |  |  |  |  |  |
| Gladius | 100 | 67.2 ± 5.8 | 1.3 ± 0.28 | 6.2 ± 0.43 | 4.12 ± 0.21 | 51.8 ± 4.7 | 20.6 ± 3.6 |
| Drysdale | 100 | 84 ± 23.9 | 0.54 ± 0.15 | 6.17 ± 1.25 | 4 ± 0.2 | 39 ± 6.6 | 42.6 ± 12 |
| GD0014 | 100 | 78.8 ± 22.9 | 2.8 ± 0.7 | 6.25 ± 0.49 | 4.53 ± 0.51 | 57 ± 7 | 9.8 ± 3 |
| GD0120 | 100 | 71 ± 15.2 | 2.27 ± 0.21 | 6.03 ± 1.22 | 3.52 ± 0.1 | 53.8 ± 16 | 11.2 ± 1.2 |
| GD0180 | 100 | 65 ± 2 | 2.32 ± 0.21 | 5.3 ± 0.3 | 4.17 ± 0.21 | 51.7 ± 4.5 | 11.3 ± 1.5 |
| GD0185 | 100 | 75.2 ± 7.7 | 3.01 ± 0.44 | 6.12 ± 0.26 | 4 ± 0.18 | 59 ± 3.5 | 8.7 ± 1.8 |
| ***Anova analysis (P value)*** | |  |  |  |  |  |  |
| Genotype (G) | | 0.185 | <0.001 | 0.961 | <0.001 | 0.087 | <0.001 |
| Treatment (T) | | <0.001 | <0.001 | 0.042 | <0.001 | <0.001 | <0.001 |
| G x T | | 0.585 | <0.001 | 0.186 | 0.008 | 0.007 | <0.001 |
